# Efferocytosis-Related Transcriptional Dysregulation in Hidradenitis Suppurativa Lesional Macrophages: A Multi-Cohort Single-Cell and Bulk Transcriptomic Study

**DOI:** 10.3390/biomedicines14102322

**Published:** 2026-10-13

**Authors:** Mahir Dığış, Kısmet Kaya, Betül Demir

**Affiliations:** Department of Dermatology and Venereology, Faculty of Medicine, Fırat University, 23119 Elazığ, Türkiye; k_kaya@firat.edu.tr (K.K.);

**Keywords:** hidradenitis suppurativa, efferocytosis, macrophages, single-cell RNA sequencing, transcriptomics, inflammation resolution, gene set analysis, adalimumab

## Abstract

**Background/Objectives:** Hidradenitis suppurativa (HS) is a chronic inflammatory skin disease in which inflammation progresses to tunnels and fibrosis rather than resolving. Efferocytosis, the macrophage clearance of apoptotic cells, is a central mechanism of inflammation resolution, but its transcriptional status in HS is uncharacterized. We investigated whether an externally derived 167-gene efferocytosis-related panel (ER167) is transcriptionally perturbed in HS. **Methods:** Public Gene Expression Omnibus datasets were assigned non-overlapping roles. The primary endpoint was a donor-level, mixed-direction gene-set test of ER167 in monocyte/macrophage pseudobulk from a discovery single-cell RNA-sequencing (scRNA-seq) cohort, benchmarked against 10,000 expression- and detection-matched random panels and compared with established efferocytosis and inflammation gene sets. Analyses extended to independent scRNA-seq replication, three bulk-tissue cohorts, and 12-week adalimumab and brodalumab data. **Results:** In discovery macrophages, 141 of 167 ER167 genes were detected, and 62 were differentially expressed (36 increased, 26 decreased; mixed-direction *p* = 8.56 × 10^−7^). The observed statistic exceeded all 10,000 matched random panels (empirical *p* = 1/10,001). This was not specific to ER167: canonical efferocytosis gene sets (*p* = 0.0038 and 0.0054) and the Hallmark inflammatory-response set (*p* = 1/10,001) also exceeded their matched nulls, whereas interferon-response sets did not. The ER167 signal replicated in an independent scRNA-seq cohort (empirical *p* = 0.0296) and was present at whole-tissue level in three bulk cohorts (*p* = 8.56 × 10^−8^, 9.75 × 10^−11^, 2.39 × 10^−9^). No ER167 change was detectable at week 12 of biologic treatment (*p* = 0.378 and 0.754). **Conclusions:** HS lesional macrophages show a reproducible, mixed-direction perturbation of efferocytosis-related genes that accompanies an equally strong general inflammatory-response signal; no change was detectable after 12 weeks of biologic therapy. Transcript abundance does not measure efferocytic function; functional studies are required.

## 1. Introduction

Hidradenitis suppurativa (HS) is a chronic inflammatory skin disease characterized by persistent and recurrent inflammation that may progress to abscess formation, epithelialized tunnels, tissue destruction, and fibrosis rather than undergoing effective resolution [1,2]. Increasing transcriptomic and immunologic evidence implicates the myeloid compartment as a major component of this persistent inflammatory state. Single-cell RNA sequencing has demonstrated a shift of lesional monocytes and macrophages toward inflammatory transcriptional programs, including enhanced phagocytic and antimicrobial pathways and prominent STAT1/interferon-associated signaling, together with reduced expression of markers associated with regulatory macrophage states [3]. In parallel, clinically non-lesional HS skin shows relative depletion of regulatory myeloid populations, suggesting that impaired immunoregulatory balance may precede fully developed lesions [1]. Neutrophil extracellular traps (NETs) are also abundant in HS lesions and are associated with immune dysregulation and disease severity, further indicating a tissue environment in which inflammatory cell death and the handling of cellular debris may be relevant to disease persistence [4].

Efferocytosis is the coordinated recognition, engulfment, and processing of apoptotic cells by phagocytes, particularly macrophages, and represents a central mechanism of inflammation resolution and tissue homeostasis [5,6]. Efficient efferocytosis not only removes dying cells before membrane integrity is lost but also actively reprograms macrophages toward a pro-resolving state, including induction of mediators such as transforming growth factor-β and interleukin-10 and suppression of pro-inflammatory signaling [5,7]. Conversely, failure to clear apoptotic cells can permit their progression to secondary necrosis, with subsequent release of intracellular damage-associated molecular patterns and autoantigens that may amplify innate and adaptive immune responses [6,7]. Efferocytosis should therefore be considered not merely as a disposal mechanism, but as an integrated immunoregulatory process linking cellular clearance to termination of inflammation.

Several features of HS provide a biological rationale for investigating this process, although direct evidence remains limited. Altered keratinocyte function has been reported in HS without reduced keratinocyte viability or increased apoptosis in vitro [8]; whether the handling of dying cells is altered in lesional tissue has not been examined. Moreover, cytotoxic CD2-expressing lymphoid populations with perforin and granzyme expression have been identified adjacent to hyperplastic follicular epithelium and tunnels containing apoptotic cells, indicating that substantial cellular injury may occur within the lesional microenvironment [9]. Together with inflammatory macrophage polarization, loss of regulatory myeloid subsets, and neutrophil extracellular trap (NET) accumulation, these observations provide indirect support for altered dead-cell clearance or resolution biology in HS [1,3,4,9]. However, defective efferocytosis has not been demonstrated as a functional abnormality in HS: a recent review of impaired macrophage efferocytosis in immune-mediated inflammatory diseases does not cover HS [10], and, to our knowledge, direct measurements of apoptotic-cell clearance capacity in HS tissue have not been reported.

Importantly, the efferocytic response is transcriptionally complex and cannot be reduced to a single uniformly increasing or decreasing pathway. Recognition of apoptotic cells, internalization, intracellular processing, metabolic adaptation, and downstream resolution signaling are controlled by partly separable molecular programs [11]. Single-cell analyses have demonstrated heterogeneous and even opposing transcriptional responses among macrophage subsets exposed to apoptotic cells, indicating that aggregate expression scores may obscure biologically relevant state-specific responses [12]. Experimental disruption of chloride sensing can similarly replace the expected anti-inflammatory transcriptional response with inflammatory and oxidative-stress programs without necessarily preventing corpse degradation, demonstrating that engulfment and downstream resolution signaling can become uncoupled [13]. More broadly, comparable efferocytic events can generate distinct transcriptional responses depending on macrophage origin and cellular cargo [14], whereas macrophages carrying a high apoptotic-corpse burden can be compromised in subsequent clearance [15]. Thus, efferocytosis-related gene expression is informative regarding molecular programming but should not be regarded as a direct surrogate for functional clearance capacity.

The interpretation of such transcriptomic data is further constrained by study design. Single-cell studies of human skin usually include few donors, and differential-expression analyses that treat individual cells rather than donors as replicates are biased and prone to false discoveries [16]; inference therefore has to be made at the donor level, where a small number of donors limits statistical power and leaves results sensitive to individual donors. In bulk tissue, a gene-set signal reflects both expression within cells and the cellular composition of the sample. Composition can be estimated by deconvolution with a single-cell reference, but the estimates depend on the method and become substantially less accurate when cell types present in the tissue are missing from the reference [17]. Finally, a gene list rich in myeloid and inflammatory genes may be perturbed in any inflamed tissue, so that its specificity can be judged only against established efferocytosis gene sets and general inflammatory gene sets tested under the same conditions.

Against this background, we investigated whether an externally derived 167-gene efferocytosis-related panel (ER167), applied without modification, exhibits coordinated transcriptional perturbation in HS. The study was designed around donor-level macrophage analysis in a discovery single-cell RNA-sequencing cohort, with leave-one-donor-out sensitivity analysis and comparison with canonical efferocytosis and general inflammatory gene sets, followed by independent single-cell replication, validation across bulk-tissue cohorts with adjustment for estimated cell composition, assessment of macrophage-state composition and within-state transcriptional changes, and evaluation of treatment-associated expression patterns. Because efferocytosis comprises transcriptionally separable components and may therefore exhibit simultaneous increases and decreases across its molecular machinery, the primary hypothesis concerned mixed-direction perturbation of the ER167 program rather than a uniform increase or decrease. Accordingly, the analysis was intended to identify efferocytosis-related transcriptional dysregulation in HS while explicitly avoiding inference that transcriptomic changes alone demonstrate impaired, enhanced, or restored efferocytic function.

## 2. Materials and Methods

### 2.1. Study Design and Analytical Framework

This transcriptomic study evaluated whether an externally derived efferocytosis-related gene panel is altered in hidradenitis suppurativa (HS) lesional skin and macrophages. The workflow was organized into discovery, independent single-cell replication, bulk-tissue validation, compositional analysis, and longitudinal treatment assessment. Publicly available Gene Expression Omnibus (GEO) datasets were assigned non-overlapping analytical roles. The primary endpoint was a donor-level, mixed-direction gene-set test of the 167-gene efferocytosis-related panel (ER167) in macrophages from the discovery single-cell RNA-sequencing (scRNA-seq) cohort. Transcript abundance was treated as a molecular readout; no transcriptomic result was interpreted as a direct measurement of efferocytic uptake or clearance capacity.

### 2.2. Externally Derived ER167 Efferocytosis-Related Gene Panel

The efferocytosis-related gene panel was taken without modification from the published supplementary efferocytosis-related gene list of Sun et al., which contains 167 unique gene symbols [18]. Although the main Methods text of the source article refers to 157 efferocytosis-related genes, its accompanying supplementary gene list contains 167 unique entries; because the present study adopted the panel directly from this supplementary gene list, all 167 listed genes were retained without addition or exclusion [18]. Gene symbols were subsequently harmonized to current HUGO Gene Nomenclature Committee (HGNC) nomenclature with the MyGene.info gene-annotation service, using symbol, alias, and previous-symbol mappings while retaining the originally reported symbols for traceability. Two historical symbols were updated: *HIST1H1C* to *H1-2* and *FAM49A* to *CYRIA*. These one-to-one nomenclature updates did not alter panel membership or panel size, and all 167 genes mapped uniquely to Ensembl and Entrez identifiers. Genes were manually assigned by the authors to five descriptive functional categories: recognition/tethering (*n* = 35), engulfment/processing (*n* = 30), post-engulfment/resolution (*n* = 41), inflammatory/myeloid context (*n* = 45), and other or uncategorized (*n* = 16). These categories are descriptive labels and are not derived from an ontology: a Gene Ontology annotation corresponding to the label was present for 9 of 35, 4 of 30, 7 of 41 and 14 of 45 genes in the four named categories, and 17 of the 167 genes belong to Gene Ontology apoptotic cell clearance (GO:0043277) or KEGG efferocytosis (hsa04148) (Table S13). The complete panel, original and current symbols, stable identifiers, and category annotations are provided in Table S1.

### 2.3. Dataset Identification, Eligibility Audit, and Analytical Roles

Seventeen GEO series comprising 717 sample-level records were cataloged and evaluated for modality, raw-count availability, sample-to-donor mapping, disease and tissue structure, and suitability for donor-level inference (Table S2). For scRNA-seq cohorts, formal macrophage analysis additionally required at least 30 monocyte/macrophage cells per donor. The primary discovery cohort was GSE249793 [19]. GSE212721 [20] was designated the formal independent scRNA-seq replication cohort, whereas GSE220116 [21] was retained as a conditional descriptive replication because only one eligible healthy-control donor remained after cell-count gating. Bulk validation used paired lesional versus non-lesional GSE249027 [22], paired lesional versus perilesional GSE151243 [23] (the comparator tissue is described as perilesional in the GEO record and as non-lesional in the source publication), and external HS-lesional versus healthy-skin GSE154773 [24]. GSE213761 [25] and GSE189266 [26] were used to examine 12-week adalimumab and brodalumab treatment-associated changes, respectively. Whole-skin scRNA-seq data from GSE274880 [9] (six HS lesional and two normal skin samples) served only as the reference for bulk deconvolution. Eligibility decisions and ER167 mapping coverage are summarized in Table S3.

### 2.4. Discovery scRNA-Seq Preprocessing and Quality Control

Ten skin libraries from GSE249793 (five HS lesional and five healthy control) were processed; matched blood libraries were excluded. According to the GEO record, these libraries consist of CD3-negative cells sort-purified from enzymatically digested skin; the record does not report donor age, sex, anatomical site, disease stage or treatment. Ambient RNA contamination was estimated separately for each library with decontX using filtered count matrices. Decontaminated counts were rounded to integers for preprocessing and annotation, while original pre-decontX counts were retained for donor-level differential-expression modeling. Cells were retained when mitochondrial read proportion was <10%, at least 200 genes were detected, and total counts were at least 500. Mitochondrial thresholds of 15% and 20% were evaluated as sensitivity analyses. Doublets were identified per donor with scDblFinder and removed. Normalization used scran pooling size factors, and the top 2000 highly variable genes, estimated with donor as a blocking factor, were used for a 30-component principal-component analysis. fastMNN batch correction was applied only to clustering and visualization and was never used as input for differential expression. A shared-nearest-neighbor graph (k = 20) and Louvain community detection produced 16 clusters across 33,078 singlet cells; a uniform manifold approximation and projection (UMAP) representation was generated from the corrected embedding. Per-donor quality-control metrics and mitochondrial-threshold sensitivity are summarized in Table S4 (Figures S1–S3).

### 2.5. Lineage Annotation and Monocyte/Macrophage Subclustering

Major cell lineages were assigned by canonical marker scoring. The monocyte/macrophage compartment was then isolated and re-clustered, and states were named conservatively according to marker profiles, including Mac_C1QC_MRC1, Mac_MMP9_SPP1, Mac_HLADR_APC, Mono_FCN1_CCL3, Mono_FCN1_infl, and MonoMac_hypoxic_lipid. M1/M2 labels were not used (Figure S4). A LAMP3-positive/FLT3-positive migratory dendritic-cell population, a B-cell-contaminated cluster, and a heat-shock/stress cluster were excluded from the monocyte/macrophage compartment. This procedure retained 9642 monocyte/macrophage cells. One HS donor (HS419) contained 14 eligible monocyte/macrophage cells and failed the ≥30-cell gate; the donor was excluded from the primary pseudobulk analysis but retained for sensitivity analysis, leaving four HS and five healthy-control donors.

### 2.6. Donor-Level Pseudobulk Differential Expression

For each eligible donor, raw pre-decontX counts were summed across monocyte/macrophage cells to create one pseudobulk profile. Raw rather than decontX-adjusted counts were used for differential expression because negative binomial count models assume unmodified integer counts, whereas decontX returns model-based estimates whose gene-level uncertainty is not propagated into the test; ambient-RNA correction was therefore used only for clustering and annotation, and the effect of substituting decontX-adjusted counts was examined as a sensitivity analysis (Section 2.8). Lowly expressed genes were filtered with edgeR filterByExpr, library sizes were normalized by the trimmed mean of M-values method, and dispersions were estimated before robust quasi-likelihood fitting. The HS-versus-control contrast was tested with glmQLFTest. Gene-level results included log2 fold-change, approximate 95% confidence intervals (log2 fold-change ± 1.96 × standard error, with the standard error derived from the quasi-likelihood F statistic), nominal *p*-values, and Benjamini-Hochberg false-discovery rates (FDRs; Table S5). The donor, rather than the individual cell, was the unit of inference.

### 2.7. Primary ER167 Gene-Set Test and Matched-Null Analysis

The primary endpoint was a self-contained, mixed-direction ER167 test using the Fry and rotation gene set testing (ROAST) procedures on voom-transformed donor pseudobulk data. Fry and ROAST are self-contained tests: they ask whether the genes of the set are differentially expressed between groups, without reference to genes outside the set. A mixed-direction statistic was selected because biologically relevant remodeling may contain simultaneous increases and decreases and therefore may not produce a significant net directional score. The directional test was nevertheless reported as a complementary result. When voom observation weights are supplied, limma computes the residual effects used by Fry from a separate weighted QR decomposition for each gene, and the default gene-wise standardization uses the largest squared residual effect; as a consequence, the mixed-direction *p*-value depends on the order in which the samples are listed and on the computing platform, although the fitted model is the same. All Fry *p*-values in the main text were therefore computed with an order-invariant specification (voom log2 counts per million with the observation weights removed and standardize = “none”), which gave identical *p*-values across random sample orderings; the exceptions are the replication cohorts GSE212721 and GSE220116, for which the order-invariant value could not be computed from the archived objects and the weighted test in the original sample order is reported. The values obtained with the default weighted test in the original sample order, and their median and 2.5th–97.5th percentiles over 1000 random sample orderings (200 for sensitivity and comparator analyses) are given in Table S11 and Figure S7, together with a second order-invariant specification in which each gene is first divided by its residual standard deviation. The matched-null statistic does not depend on sample order. The matched-null analysis addressed a different, competitive question: whether ER167 genes are more strongly perturbed than other genes of comparable expression in the same data. The eligible gene universe comprised all genes retained after expression filtering in the respective pseudobulk or bulk dataset (7155 genes in the discovery analysis). Each gene was assigned to one of eight equal-sized rank bins of mean expression (edgeR average log2 counts per million) and to one of eight equal-sized rank bins of detection rate, defined as the proportion of donor-level pseudobulk profiles (or bulk samples) with a non-zero count, giving 64 joint bins; ties were broken by gene order. Each random set was drawn without replacement, excluding ER167 genes, so that it contained the same number of genes as ER167 in every joint bin, and 10,000 sets were drawn with a fixed random seed. In the discovery analysis, ER167 genes occupied 44 of the 64 joint bins, each of which contained at least 67 other genes. Because 6776 of the 7155 genes (94.7%) were detected in all nine donor profiles, matching was determined mainly by mean expression. For each set, the statistic was the mean squared moderated t value from a limma model fitted to the voom-transformed data (lmFit followed by eBayes with an intensity-dependent trend); the empirical *p* value was calculated as (1 + number of random statistics at least as large as the observed statistic)/10,001. The same approach was applied to each ER167 functional category. Per-cell UCell scores were summarized by donor as a complementary expression-score measure and were not used as the primary inferential test (Tables S5 and S6).

### 2.8. Panel-Specificity and Parameter-Sensitivity Analyses

To examine whether the ER167 result was specific to this panel, three analyses were performed in the discovery pseudobulk data, each using the Fry test and an expression- and detection-matched null distribution drawn separately for the gene set concerned. First, established gene sets were tested: Gene Ontology apoptotic cell clearance (GO:0043277), phagocytosis (GO:0006909), phagocytosis, engulfment (GO:0006911), phagosome maturation (GO:0090382) and macrophage activation (GO:0042116); Reactome Fcγ receptor-dependent phagocytosis; KEGG lysosome and KEGG efferocytosis (hsa04148); and the Hallmark interferon-α response, interferon-γ response, inflammatory response and TNF-α signalling via NF-κB gene sets. Two comparator panels typed manually by the authors from canonical marker genes (general inflammation and myeloid activation; 38 genes each, listed in Table S6) were also tested, and empirical *p*-values were adjusted by the Benjamini-Hochberg procedure across the 14 gene sets. Second, ER167 was retested after exclusion of the manually assigned inflammatory/myeloid-context and uncategorized genes, and after exclusion of each category in turn. Third, ER167 was retested after removal of the genes shared with the established inflammatory, interferon, macrophage-activation and phagocytosis, phagosome or lysosome gene sets, and after restriction to the genes shared with the two efferocytosis gene sets. The established gene sets and the reduced ER167 sets were also tested in the three bulk cohorts (Table S12; Figure S10). Analytical robustness was evaluated by including HS419 under a ≥10-cell rule, substituting decontX-corrected counts for raw counts, and replacing the mean-squared-t matched-null statistic with the mean absolute t statistic. Minimum counts of ≥20 and ≥50 monocyte/macrophage cells per donor had also been specified, but they selected the same nine donors as the primary ≥30-cell gate (the smallest included donor contributed 100 cells) and therefore did not provide independent tests. The effect of the alternative mitochondrial thresholds on the number of cells retained was assessed separately (Table S4; Figure S3); the ER167 test was not repeated at these thresholds.

Donor-level structure was examined by multidimensional scaling and principal-component analysis of the donor pseudobulk profiles. The influence of individual donors was examined by repeating the complete analysis (expression filtering, normalization, dispersion estimation, gene-level testing, Fry test and matched null) after removal of each donor in turn; in addition, the deviation of the ER167 expression of each donor from the mean of the control donors among the other eight was correlated with the ER167 log2 fold changes estimated from those eight donors (leave-one-out projection). Donor metadata was taken from the GEO sample records; apart from the tissue descriptor, which coincides with the disease group, the only variables that varied between donors were the number of monocyte/macrophage cells and the pseudobulk library size, each of which was added to the model as a sensitivity check. The effect sizes detectable with the discovery design (four versus five donors) and with a three-versus-three within-state design (three HS and three control donors of the Mono_FCN1_infl state, the minimum number of donors per group required for a within-state test) were estimated by simulation. Negative binomial counts were generated from the fitted discovery pseudobulk (tagwise dispersions taken as true values; genes simulated independently), a fraction π of the ER167 genes (0.10, 0.25 or 0.50) was shifted by ±δ log2 units (δ from 0.5 to 2) in random directions, and the rejection rate of the order-invariant Fry test at *p* < 0.05 was recorded over 200 replicates per condition; the rejection rate at δ = 0 gave the type I error. Gene-level power at FDR < 0.05 was estimated in the same way over 100 replicates (Tables S15 and S17; Figures S8, S9 and S12).

### 2.9. Macrophage-State Abundance and Within-State Analysis

Differences in monocyte/macrophage-state proportions between HS and control donors were tested with propeller in the speckle package using an arcsine-square-root transformation and FDR correction. To evaluate whether the aggregate ER167 result could be explained entirely by shifts in cell-state composition, donor-level pseudobulk profiles were generated within individual states. Mixed-direction ER167 tests were repeated in states represented by at least 10 cells in at least three donors per group. These within-state tests were treated as exploratory: they indicate whether a transcriptional component remains detectable after analysis is restricted to broadly defined cell states, but they cannot exclude residual heterogeneity within a state and do not demonstrate cell-autonomous regulation. To examine the dependence on the cell-count threshold, the tests were repeated with minimum counts of 20, 30 and 50 cells per donor and state, with state-specific matched-null distributions, with Benjamini-Hochberg and Holm adjustment across the testable states, and after removal of each donor in turn (Tables S7 and S16; Figure S11).

### 2.10. Independent scRNA-Seq Replication and Gene-Level Concordance

Replication datasets were processed independently and were never merged with discovery data. According to its GEO record, GSE212721 consists of CD45-positive cells sorted from cryopreserved skin digests; the HS samples were excised surgical tissue and the control samples were skin from mammoplasty. For GSE212721, unfiltered droplet matrices were subjected to emptyDrops cell calling at FDR < 0.001 before ambient-RNA correction and the common scRNA-seq workflow. Donor-level pseudobulk ER167 testing and the 10,000-set matched-null procedure were then repeated without panel modification. Gene-level replication was assessed among ER167 genes shared by discovery and replication using Pearson correlation of log2 fold changes, overall sign concordance with a binomial test against 50%, and sign concordance among genes significant in discovery. For GSE220116, HS epidermal, dermal, and total-skin fractions were merged at the donor level and psoriasis samples were excluded; because the final analysis contained five HS donors but only one control donor, results were considered descriptive and no claim of formal replication was made (Table S8; Figure S5).

### 2.11. Bulk-Transcriptomic Validation and Tissue Deconvolution

Bulk RNA-seq cohorts were analyzed with limma-voom. Because bulk profiles represent whole tissue, these analyses were treated as whole-tissue validation and not as macrophage-specific replication. GSE249027 (15 patients, lesional versus non-lesional) and GSE151243 (20 patients, lesional versus perilesional) used within-patient blocking; the analyzed subset of GSE154773 compared 22 HS lesional samples with 10 healthy controls. ER167 was evaluated with Fry and the same 10,000-set matched-null procedure. To examine the contribution of cellular composition, a single-cell reference was derived from GSE274880 (eight libraries; 21,378 cells assigned to eight broad classes by cluster-level marker scoring). The reference was dominated by lymphocytes (T cells 53.5%, B/plasma cells 12.5%, natural killer cells 11.0%) and contained few monocytes/macrophages (2.5%). The class labeled keratinocyte (221 cells; 1.0%) was not keratinocyte-like: its most specific genes included plasmacytoid dendritic-cell genes (*LILRA4*, *CLEC4C* and *PTCRA*); *PTPRC* was detected in 85% of its cells; and, although *KRT5* was detected in 63% of its cells, *KRT14* and *KRT1* were detected in fewer than 1%. The monocyte/macrophage class probably also contained Langerhans or other dendritic cells, because *CD207* and *CD1A* were detected almost exclusively in this class (in 2.2% and 4.5% of its cells). The class labels, which had been assigned by cluster-level marker scoring, were left unchanged. The reference therefore contains essentially no keratinocytes and does not represent the composition of whole skin (Table S14). Six estimates of monocyte/macrophage content were compared: (M1) non-negative least squares (NNLS) deconvolution on the 2000 genes with the greatest cross-class variation; (M2) the same procedure after exclusion of all ER167 genes from the signature; (M3) NNLS restricted to class marker genes (150 per class), with each gene scaled by its standard deviation across classes; (M4) MuSiC and (M5) BisqueRNA, both using 300 marker genes per class; and (M6) a marker-gene score, calculated as the mean z-standardized log2 counts per million of *CD14*, *CSF1R*, *AIF1*, *ITGAM*, *FCGR1A* and *CD86* (*FCGR1A* did not pass expression filtering in GSE249027). ER167 genes were excluded from the genes used by M2–M6. Three further candidate markers (*MS4A6A*, *MAFB* and *LILRB4*) were not most highly expressed in the monocyte/macrophage class of the reference and were excluded before any bulk analysis. M1–M5 were evaluated on pseudobulk mixtures of known composition, generated either by leaving out one reference library at a time and drawing 20 bootstrap samples of its cells (scheme A), or by splitting the libraries into two folds of four and deconvolving the 80 bootstrap mixtures of the held-out fold together (scheme B; needed for BisqueRNA, which calibrates on between-sample variation). For each bulk sample, the ER167 score was calculated as the mean z-standardized logCPM across detected ER167 genes and correlated with each estimate using Spearman correlation. In the paired cohorts, the ER167 disease model was repeated with (i) the estimated monocyte/macrophage fraction and (ii) all estimated immune-cell classes as covariates; attenuation was calculated as the proportional reduction in the mean squared moderated t statistic of ER167 (Tables S9, S10 and S14).

### 2.12. Longitudinal Treatment and Disease-Treatment Vector Analyses

Treatment-associated ER167 changes were evaluated between lesional baseline and week 12. GSE213761 included 14 patients with paired lesional samples before and after adalimumab; a subject blocking factor was used, and the paired lesional-non-lesional disease gap was also tracked where available. Because donor linkage was unavailable for GSE189266, brodalumab lesional samples were analyzed cross-sectionally by time point (nine baseline and nine week-12 samples). Fry provided mixed-direction and directional gene-set tests. For each cohort, the change in the ER167 score (mean z-standardized logCPM across detected ER167 genes) was summarized by its mean, 95% confidence interval and standardized effect size (Hedges g), and the smallest change detectable with 80% power at a two-sided α of 0.05 was calculated from the observed standard deviation. Responder status in GSE213761 was taken from the GEO sample field “responder” (seven responders and seven non-responders among the 14 paired patients). The GEO record does not define the criterion; the source publication assessed response with the Hidradenitis Suppurativa Clinical Response and the International Hidradenitis Suppurativa Severity Score System [25]. The comparison between responders and non-responders was exploratory (Table S18). A second analysis compared ER167 gene-level disease log2 fold-change vectors from GSE154773 and GSE249027 with treatment-associated vectors for each drug. Reversal would be expected to produce a negative correlation. Correlations were quantified with Spearman coefficients. Because the genes of the panel are not independent, conventional gene-level *p*-values were not used, and each correlation was instead compared with two reference distributions. First, the sample labels were permuted 10,000 times (exchange of the baseline and week-12 labels within patients for adalimumab; permutation of the time-point labels among the 18 lesional samples for brodalumab); in each permutation the treatment model was refitted and the correlation with the unchanged disease vector was recalculated, and the two-sided empirical *p* value was calculated as (1 + the number of permutations with an absolute correlation at least as large as that observed)/10,001; a one-sided *p* value for a negative correlation was calculated in the same way. Second, the ER167 correlation was compared with the correlations of 10,000 random gene sets of the same size, matched for decile of mean expression (Tables S10 and S18; Figure S6).

### 2.13. Software, Multiplicity Control, and Reproducibility

All statistical analyses were performed in R 4.5.3 with Bioconductor 3.22 packages, and a fixed random seed (set.seed(1)) was set before every stochastic step. Series and sample metadata were retrieved programmatically from GEO (the sample records used for Table S15 with GEOquery v2.78.0), and count matrices were obtained from the supplementary files of each GEO series. ER167 gene symbols were harmonized to current HGNC symbols and mapped to Ensembl and Entrez identifiers with the MyGene.info gene-annotation service (queried in August 2026). Single-cell data were handled within the SingleCellExperiment v1.32.0 framework with Matrix v1.7-5; droplet-level cell calling, ambient-RNA correction, doublet detection, normalization, and batch handling used DropletUtils v1.30.0, celda/decontX v1.26.0, scDblFinder v1.24.0, scran v1.38.0, scater v1.38.0, and batchelor v1.26.0, and Louvain community detection used igraph v2.3.3. Donor-level pseudobulk differential expression used edgeR v4.8.2 (filterByExpr, trimmed mean of M-values normalization, and quasi-likelihood F-tests); bulk differential expression, the mixed-direction and directional gene-set tests (fry and roast), and the moderated t statistics of the matched-null analysis used limma v3.66.0 (voom, lmFit, eBayes) with statmod v1.5.2; complementary expression scoring used UCell v2.14.0, and cell-state abundance testing used propeller in speckle v1.10.0. Established gene sets were obtained from MSigDB 2026.1.Hs through msigdbr v26.1.1 and from KEGG through KEGGREST v1.50.0 (accessed 3 October 2026), and Gene Ontology annotations of the panel genes from org.Hs.eg.db v3.22.0. The comparator general-inflammation and myeloid-activation panels were curated manually by the authors, and their gene lists are given in Table S6. The NNLS deconvolution M1 was first run with SciPy v1.17.1 in Python 3.11 and reproduced with nnls v1.6 in R; the other composition estimates used nnls v1.6, MuSiC v1.0.0 and BisqueRNA v1.0.5 in R; and figures were produced with matplotlib v3.11.2 (Figures S1–S4, which date from the original analysis, with v3.11.0). Gene-level multiplicity was controlled by the Benjamini-Hochberg procedure. Gene-set *p*-values for the primary endpoint and for each replication, validation, and treatment cohort are reported without adjustment because each addresses a single hypothesis; category-level and within-state *p*-values are reported as nominal values together with Benjamini-Hochberg-adjusted values across the categories or states tested; the empirical *p*-values of the 14 gene sets compared in the specificity analysis were adjusted in the same way. Unless otherwise specified, tests were two-sided, and matched-null empirical *p*-values were calculated using 10,000 random sets with a minimum attainable value of 1/10,001. The order-sensitivity, specificity and composition analyses were run on a second computer (R 4.5.3 for Windows) with the same versions of edgeR, limma and statmod; 19 of 20 key quantities of the first run were reproduced within 5%, the exception being the weighted Fry *p*-value of GSE151243 (2.59 × 10^−14^ against 2.10 × 10^−14^ in the first run), which depends on sample order and platform (Section 2.7). The ER167 panel, cohort metadata, gene-level results, sensitivity analyses, and figure source tables are provided in the Supplementary Materials. The analysis scripts of all stages, including the analyses added during peer review, the configuration file with thresholds and random seeds, the panel file, the GEO sample metadata, the gene-set definitions and the environment files are provided as a compressed archive (Supplementary File S1). The scripts for pseudobulk differential expression, gene-set testing, state abundance, bulk validation, deconvolution and treatment analyses were re-run from the layout of this archive and reproduced the archived results, with the same exception; the panel-harmonization, preprocessing, annotation and replication scripts, the construction of the deconvolution reference and the supplementary-analysis scripts of the original analysis require source files, the raw GEO data or objects derived from them and were not re-run; the status of every script, including those whose outputs could not be compared with an archived file, is given in File S1 (VERIFICATION.md). The analyses were carried out in Claude Science (Anthropic, San Francisco, CA, USA), an analysis environment based on a generative artificial intelligence model, in which the analysis code was generated and executed under the direction of the authors; the authors checked the code and the results, and the code and a verification record of the scripts are included in File S1.

## 3. Results

### 3.1. An Externally Derived ER167 Panel and Role-Defined Multi-Cohort Design

**Figure 1:**
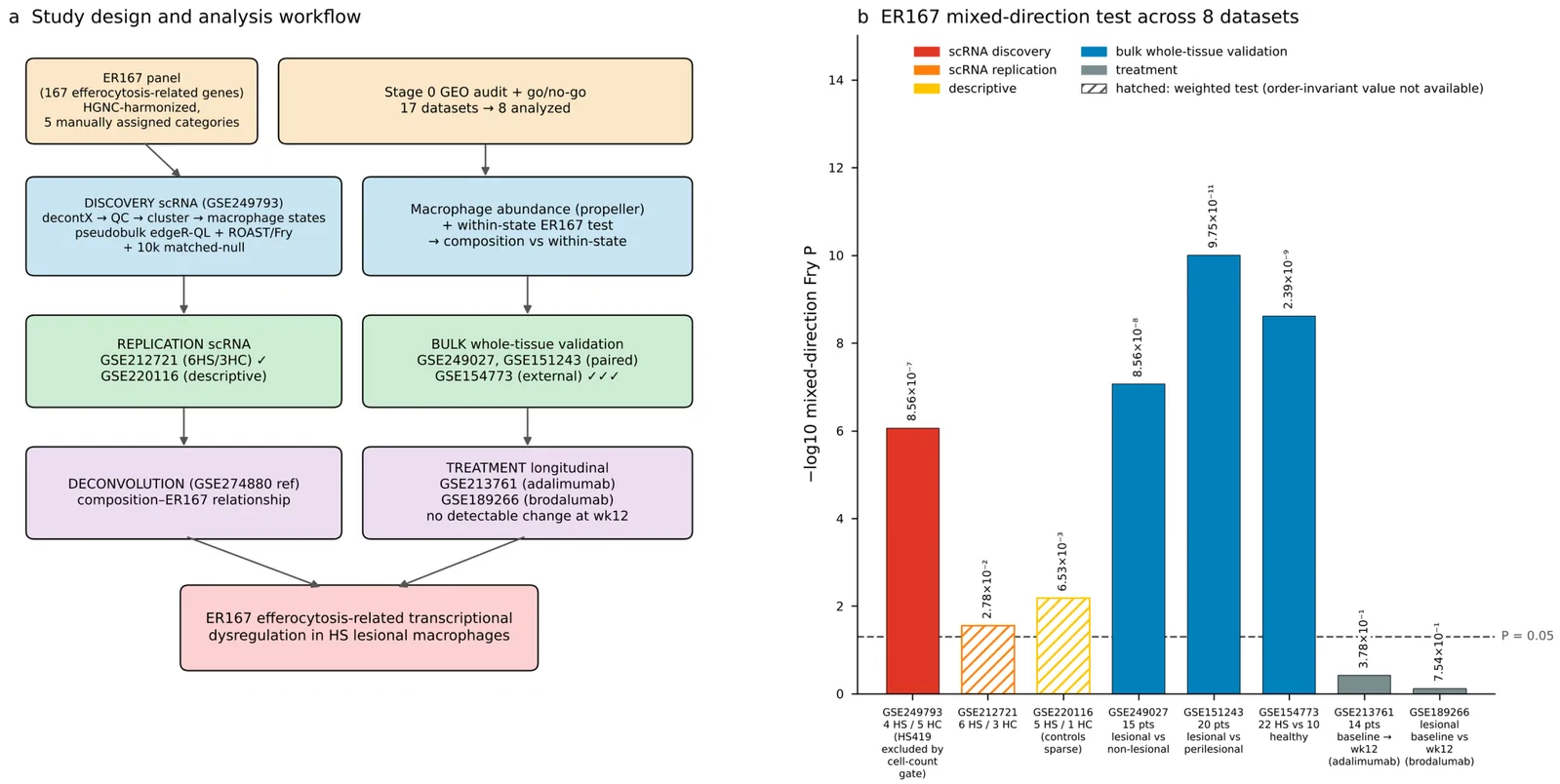
ER167 study design and cross-cohort summary. Panel (**a**) shows the role-defined workflow from the ER167 panel and Stage-0 audit through discovery, replication, bulk validation, deconvolution, and treatment analyses. Panel (**b**) presents −log_10_ mixed-direction Fry *p*-values. Solid bars show the order-invariant test; for GSE212721 and GSE220116, for which the order-invariant value could not be computed from the archived objects, hatched bars show the weighted test in the original sample order. The dashed line marks *p* = 0.05. The two treatment contrasts were not significant. Check marks indicate statistically significant ER167 results in the formal replication and bulk-validation cohorts.

The ER167 panel contained 167 efferocytosis-associated genes, all of which were uniquely mapped after nomenclature harmonization. The Stage-0 audit evaluated 17 GEO datasets and assigned eight datasets to formal discovery, replication, validation, or treatment roles, with GSE274880 used solely as a deconvolution reference (Table 1; Figure 1; Tables S1–S3). This structure separated hypothesis testing from replication, and panel membership was not altered at any stage of the analysis.

### 3.2. Discovery scRNA-Seq Resolved a 9642-Cell Monocyte/Macrophage Compartment

**Figure 2:**
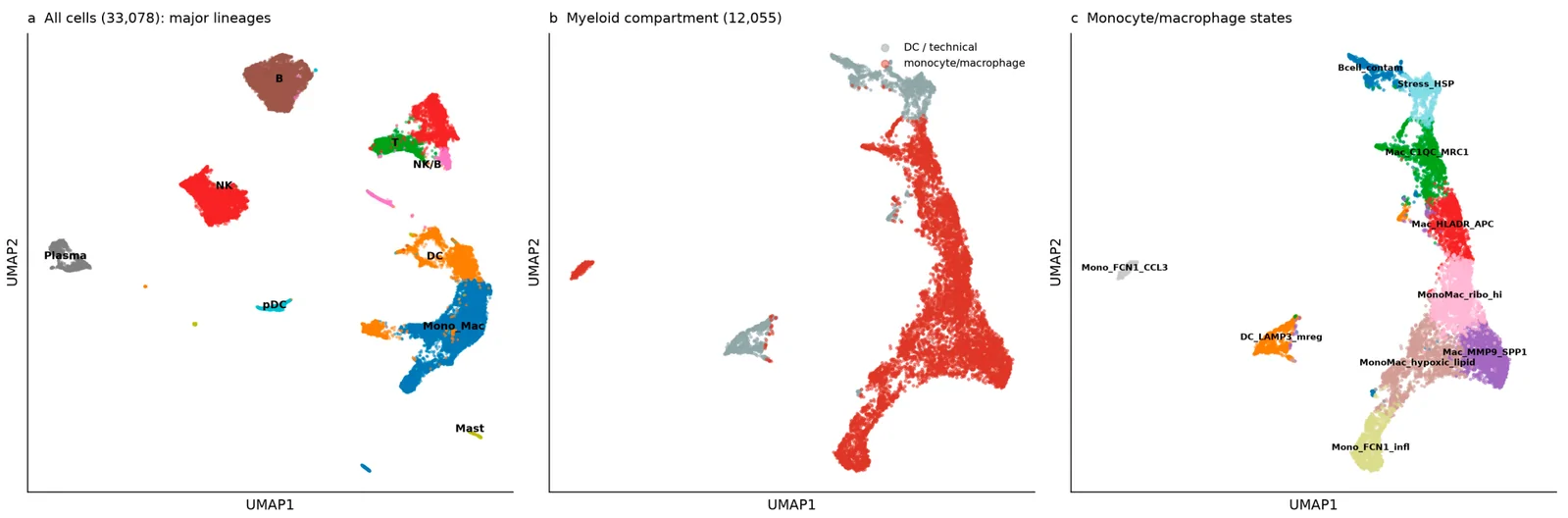
Discovery scRNA-seq atlas and monocyte/macrophage compartment in GSE249793. Panel (**a**) shows 33,078 singlet cells across major lineages, panel (**b**) separates monocyte/macrophage cells from dendritic/technical populations, and panel (**c**) displays marker-defined monocyte/macrophage states used for downstream donor-level analyses.

After quality control and doublet removal, the discovery cohort contained 33,078 singlet cells spanning nine major lineages. Re-clustering identified 9642 monocyte/macrophage cells in marker-defined resident, antigen-presenting, inflammatory, lipid-associated, and matrix-remodeling states, while migratory dendritic, B-cell-contaminated, and stress clusters were separated and excluded (Figure 2 and Figures S1–S4). HS419 failed the donor-level macrophage gate with 14 cells, leaving four HS and five healthy-control donors for the primary pseudobulk comparison.

### 3.3. ER167 Genes Showed a Mixed-Direction Perturbation in HS Macrophages

Among 7155 genes tested transcriptome-wide in discovery macrophage pseudobulk, 1996 were differentially expressed at FDR < 0.05. Of 167 ER167 genes, 141 were detected, and 62 met FDR < 0.05; 36 were increased, and 26 were decreased in HS. Representative increases included *SPP1*, *AQP9*, *TLR4*, and *IL1B*, whereas *VSIG4*, *MRC1*, *MARCO*, and CD36 were decreased (Table 2; Table S5, which lists all detected ER167 genes). The primary Fry mixed-direction test was significant (*p* = 8.56 × 10^−7^), while the net directional test was not (*p* = 0.479). ROAST supported the mixed pattern (*p* = 2.0 × 10^−4^) and estimated an active proportion of approximately 69%. With the default weighted test in the original sample order, the mixed-direction *p*-value was 5.96 × 10^−9^ (median over 1000 sample orderings 1.60 × 10^−8^; 2.5th–97.5th percentiles 9.06 × 10^−9^ to 3.23 × 10^−8^; Table S11). Because 28% of all tested genes were differentially expressed, a self-contained test is expected to be significant for most gene sets in these data; the competitive matched-null comparison (Section 3.4 and Section 3.5) is therefore the more informative result. Thus, HS macrophages did not show a uniform increase or decrease across ER167; rather, different components of the program changed in opposite directions.

### 3.4. The ER167 Signal Exceeded Matched Random Panels and Differed Between Functional Categories

**Figure 3:**
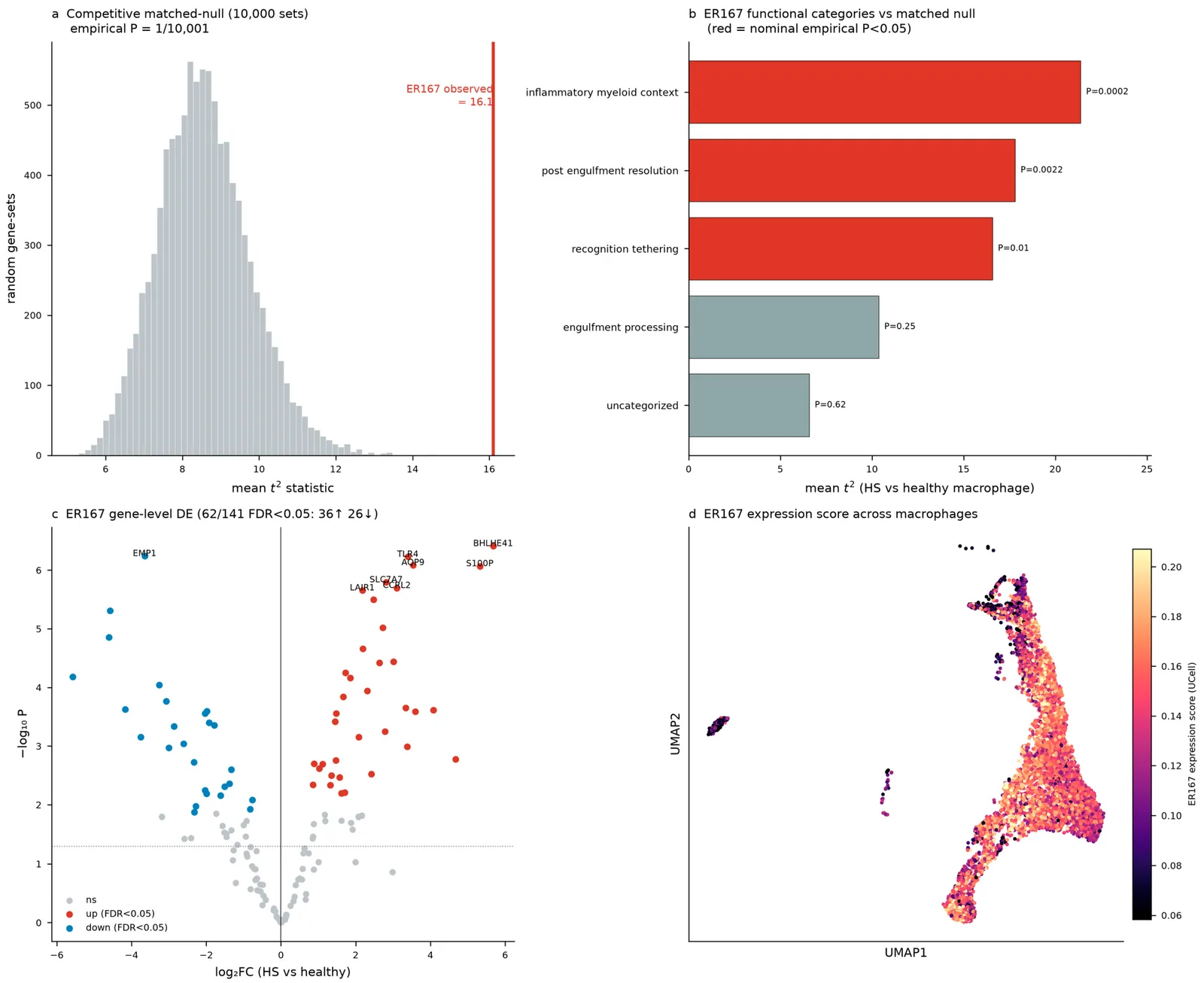
Primary ER167 analysis in discovery macrophages. Panel (**a**) compares the observed mean t^2^ statistic with 10,000 expression- and detection-matched random gene sets. Panel (**b**) reports functional-category matched-null results. Panel (**c**) shows gene-level ER167 differential expression, and panel (**d**) maps the complementary UCell expression score across the macrophage embedding. The mixed-direction matched-null result, rather than the aggregate UCell score, was the primary endpoint.

The observed ER167 mean-squared-t statistic was 16.10 and exceeded all 10,000 expression- and detection-matched random gene sets, corresponding to an empirical *p* = 1/10,001 (null distribution: mean 8.58, standard deviation 1.20, maximum 14.57) (Figure 3a; Table S6). Category-resolved testing localized the strongest signals to inflammatory/myeloid context (39 detected genes; nominal empirical *p* = 2/10,001; Benjamini-Hochberg-adjusted *p* across the five categories = 0.0010), post-engulfment/resolution (29 genes; nominal *p* = 0.0022; adjusted *p* = 0.0055), and recognition/tethering (33 genes; nominal *p* = 0.010; adjusted *p* = 0.017). Engulfment/processing was not significant relative to its matched null (29 genes; nominal *p* = 0.247; adjusted *p* = 0.31), nor was the uncategorized group (11 genes; nominal *p* = 0.62; adjusted *p* = 0.62) (Figure 3b). Because the categories were assigned manually and only a minority of the genes in each category carry a corresponding Gene Ontology annotation (Section 2.2; Table S13), these differences describe the panel and should not be read as evidence about particular stages of efferocytosis. The complementary donor-level UCell score did not differ between HS and control (*p* = 0.73), which was consistent with cancellation in a net score when increased and decreased genes coexist.

### 3.5. Specificity and Sensitivity Analyses: The Signal Was Not Specific to ER167

The matched-null result was not specific to ER167 (Table S12; Figure S10). In the discovery macrophage data, the Hallmark inflammatory-response gene set also exceeded all 10,000 of its matched random sets (140 detected genes; empirical *p* = 1/10,001; Benjamini-Hochberg-adjusted *p* across the 14 gene sets = 0.0014). The two canonical efferocytosis gene sets exceeded their matched nulls as well: Gene Ontology apoptotic cell clearance (23 detected genes; *p* = 0.0038; adjusted *p* = 0.0252) and KEGG efferocytosis (91 genes; *p* = 0.0054; adjusted *p* = 0.0252). Gene Ontology phagocytosis (*p* = 0.0307) and Hallmark TNF-α signaling via NF-κB (*p* = 0.0241) were nominally significant but not after adjustment (adjusted *p* = 0.086 and 0.0843), and Gene Ontology macrophage activation was not significant (*p* = 0.0554). The interferon-α and interferon-γ response sets (*p* = 0.46 and 0.62), Fcγ receptor-dependent phagocytosis (*p* = 0.66), phagocytosis engulfment (*p* = 0.45), phagosome maturation (*p* = 0.34) and lysosome (*p* = 0.21) did not exceed their matched nulls. Of the two manually curated comparator panels, general inflammation did not exceed its null (*p* = 0.946) and myeloid activation was borderline (*p* = 0.053). In contrast to the matched-null comparison, the self-contained Fry test was significant for 13 of the 14 gene sets and did not discriminate between them (Table S12).

ER167 continued to exceed its matched null after removal of genes that it shares with other gene sets: after exclusion of the manually assigned inflammatory/myeloid-context and uncategorized genes (91 detected genes; empirical *p* = 4/10,001); after removal of the genes belonging to the Hallmark inflammatory-response or TNF-α gene sets (108 genes; *p* = 0.0016); and after removal of the genes belonging to any of the inflammatory, interferon, macrophage-activation, phagocytosis, phagosome or lysosome gene sets (76 genes; *p* = 0.0082). The 17 ER167 genes shared with the two canonical efferocytosis gene sets (15 detected) did not exceed their matched null on their own (*p* = 0.153). Removal of the genes shared with the two manual comparator panels left 115 detected genes and a Fry *p*-value of 8.70 × 10^−8^. In the three bulk cohorts, 11, 14 and 8 of the 14 gene sets exceeded their matched nulls at nominal *p* < 0.05 (GSE249027, GSE151243 and GSE154773, respectively), so that whole-tissue data provided no evidence of specificity. With HS419 included at ≥10 cells (Fry *p* = 3.20 × 10^−7^; empirical *p* = 2/10,001), with decontX-adjusted counts (Fry *p* = 2.88 × 10^−5^; empirical *p* = 3/10,001), and with the mean absolute t statistic (empirical *p* = 1/10,001), the result was unchanged; the ≥20- and ≥50-cell gates selected the same nine donors as the primary gate and gave identical values (Table S6).

Donor-level analyses supported the stability of the primary result (Table S15; Figures S8 and S9). In principal-component analysis of the nine donor profiles, the first component (38.1% of the variance) separated all HS from all control donors (exact Wilcoxon *p* = 0.016, the smallest value attainable with four versus five donors), and the third component (13.5%) correlated with library size and cell number (Spearman ρ = 0.72 and 0.73; nominal *p* = 0.037 and 0.031, not significant after adjustment). When each donor was removed in turn, the Fry mixed *p* ranged from 9.24 × 10^−7^ to 2.04 × 10^−5^, the matched-null empirical *p* remained 1/10,001 in all nine analyses, the number of ER167 genes at FDR < 0.05 ranged from 48 to 71, and the gene-level log2 fold changes correlated with those of the full analysis at r = 0.944–0.997. In the leave-one-out projection, the ER167 profile of each HS donor correlated with the log2 fold changes estimated from the other eight donors (Pearson r = 0.74–0.89), whereas those of the control donors did so weakly or not at all (r = −0.23 to 0.43). Two control donors contributed 3066 and 3118 of the 9628 monocyte/macrophage cells of the nine donors analyzed (64%); removing either did not change the result. Adding cell number or library size as a covariate left ER167 significant (Fry *p* = 2.34 × 10^−6^ and 8.54 × 10^−7^). The between-donor biological coefficient of variation was 0.42.

In simulations of the discovery design, the power of the Fry test was 0.79 when 25% of the panel genes changed by 1 log2 unit, 0.80 when 10% changed by 1.5 log2 units, and 0.08–0.46 for changes of 0.5 log2 units. For individual genes, power at FDR < 0.05 was 0.6% for a change of 1 log2 unit, 55% for 1.5 and 82% for 2 log2 units, so that the absence of a significant change in an individual gene is uninformative for effects below about 1.5 log2 units. In the three-versus-three within-state design, power was 0.46 when 25% of the genes changed by 1 log2 unit and reached 0.87 when they changed by 1.5 log2 units. Changes of 0.5 log2 units had power below 0.5 in both designs. Under the null hypothesis, the rejection rate of the order-invariant Fry test in the discovery design was 7.5% (Monte Carlo standard error 1.9%) and that of the default weighted test 12.5%; the weighted test was therefore anti-conservative in this design. These simulations treat genes as independent and are therefore optimistic (Table S17; Figure S12).

### 3.6. Cell-State Remodeling and Within-State ER167 Perturbation

**Figure 4:**
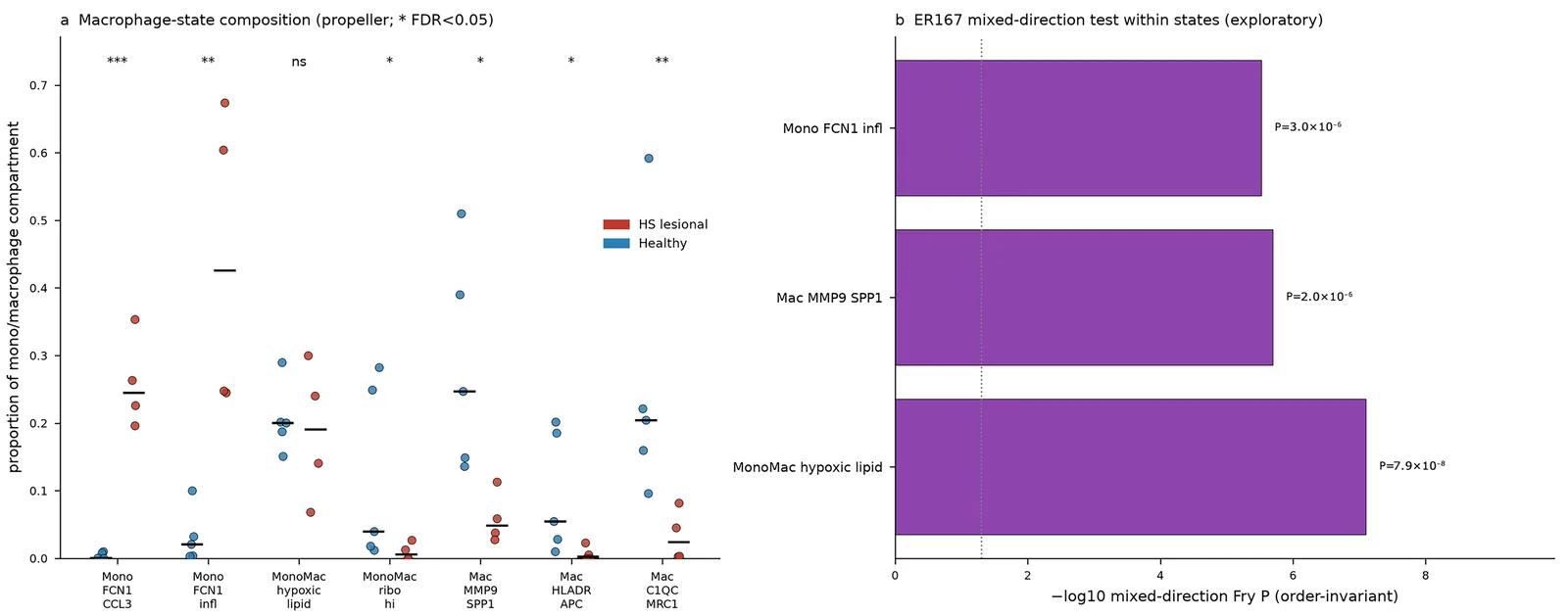
Macrophage-state remodeling and within-state ER167 analysis in GSE249793. Panel (**a**) shows donor-level state proportions tested with propeller. Panel (**b**) shows the mixed-direction ER167 tests (order-invariant Fry *p*; at least 10 cells per donor) within Mono_FCN1_infl, Mac_MMP9_SPP1, and MonoMac_hypoxic_lipid, the three states that could be tested; results at higher cell-count thresholds are shown in Figure S11. These exploratory results are compatible with a transcriptional component that remains detectable after analysis is restricted to broadly defined states. In panel (**a**), points represent individual donors and black horizontal bars indicate group medians. Asterisks indicate Benjamini–Hochberg-adjusted significance levels: * FDR < 0.05, ** FDR < 0.01, and *** FDR < 0.001; ns, not significant (FDR ≥ 0.05).

HS lesions showed expansion of Mono_FCN1_CCL3 and Mono_FCN1_infl states (FDR = 2.34 × 10^−4^ and 0.0014) and depletion of Mac_C1QC_MRC1, Mac_HLADR_APC, Mac_MMP9_SPP1, and MonoMac_ribo_hi states (FDR = 0.0084, 0.0109, 0.0168, and 0.0224); the proportion of MonoMac_hypoxic_lipid did not differ (FDR = 0.70) (Figure 4a; Table S7). When ER167 testing was restricted to individual states, three of the seven states met the donor and cell-count requirements (five control and four HS donors each). The other four could not be tested because one group had fewer than three donors with at least 10 cells (Mono_FCN1_CCL3: no control donor; MonoMac_ribo_hi and Mac_HLADR_APC: no HS donor; Mac_C1QC_MRC1: one HS donor). Mixed-direction perturbation was detectable in all three testable states: Mac_MMP9_SPP1 (61 detected ER167 genes; nominal *p* = 2.01 × 10^−6^; Benjamini-Hochberg-adjusted *p* across the three states = 2.99 × 10^−6^), MonoMac_hypoxic_lipid (86 genes; nominal *p* = 7.93 × 10^−8^; adjusted *p* = 2.38 × 10^−7^), and Mono_FCN1_infl (52 genes; nominal *p* = 2.99 × 10^−6^; adjusted *p* = 2.99 × 10^−6^) (Figure 4b). Each of the three also exceeded its own matched null (empirical *p* = 0.003, 0.0022 and 5/10,001; Benjamini-Hochberg-adjusted *p* = 0.003, 0.003 and 0.0015). The results depended on the cell-count threshold. At ≥20, ≥30 and ≥50 cells only MonoMac_hypoxic_lipid remained testable, and it remained significant (Fry *p* = 7.93 × 10^−8^, 2.95 × 10^−6^ and 7.83 × 10^−6^; matched-null empirical *p* = 0.0022, 0.0007 and 0.0034); Mac_MMP9_SPP1 and Mono_FCN1_infl could be tested only at the lowest threshold (smallest donor contribution 13 and 10 cells). When each donor was removed in turn, Mac_MMP9_SPP1 and MonoMac_hypoxic_lipid remained significant in all nine analyses, whereas Mono_FCN1_infl did not in two (maximum *p* = 0.26); the net direction within MonoMac_hypoxic_lipid changed sign between analyses (Table S16; Figure S11). The within-state result is therefore stable for MonoMac_hypoxic_lipid, rests on few cells for Mac_MMP9_SPP1, and is not robust for Mono_FCN1_infl. These exploratory findings are compatible with contributions from both altered state abundance and a within-state transcriptional component.

### 3.7. Independent scRNA-Seq Data Replicated the ER167 Pattern

**Figure 5:**
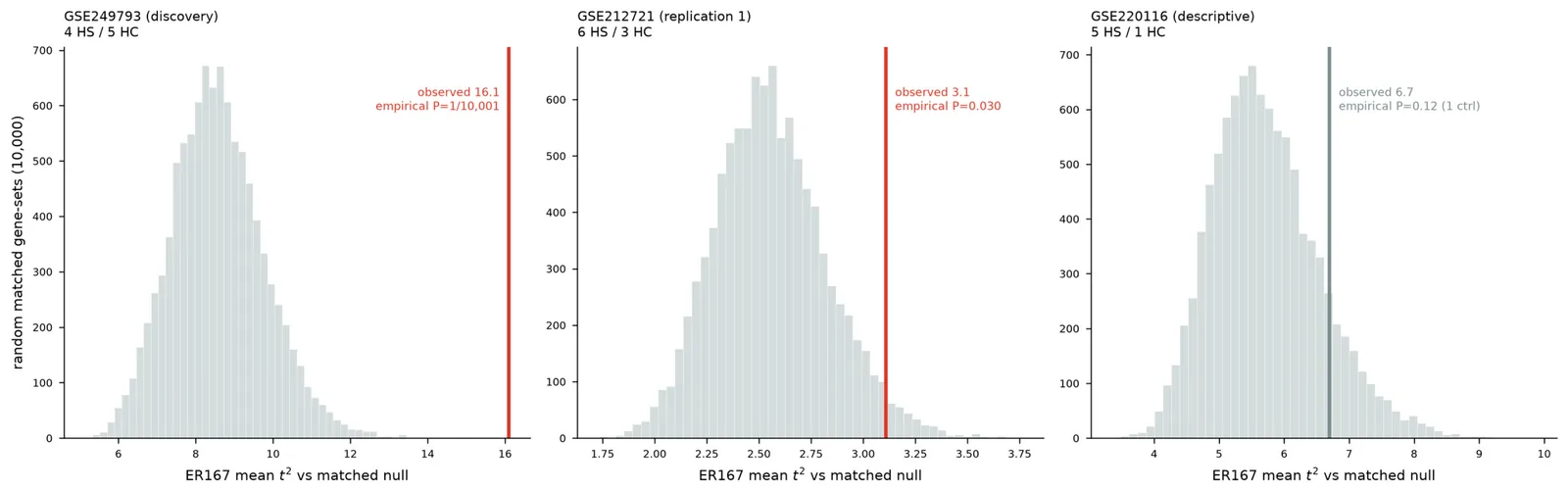
Matched-null ER167 results across scRNA-seq cohorts. GSE249793 is the discovery cohort, and GSE212721 is the formal independent replication. GSE220116 is displayed for completeness but remains descriptive because only one eligible healthy-control donor was available and its observed statistic did not exceed the matched-null distribution.

In GSE212721, analyzed independently with six HS and three healthy-control donors, ER167 remained significant by the mixed-direction Fry test (158 detected genes; weighted test in the original sample order, *p* = 0.0278) and by the matched-null comparison (empirical *p* = 0.0296); the directional test was not significant. Among 140 ER167 genes shared with discovery, log2 fold changes correlated across cohorts (Pearson r = 0.42, *p* = 1.8 × 10^−7^). Sign concordance was 64% overall (binomial *p* = 9 × 10^−4^) and rose to 79% among genes significant in discovery; within this subset, effect sizes correlated at r = 0.56 (Figure 5 and Figure S5; Table S8). GSE220116 produced a nominal mixed-direction Fry *p* = 0.0065 (weighted test) but did not exceed its matched null (empirical *p* = 0.122). Because this comparison contained five HS donors and only one eligible control, it was considered descriptive and design-confounded rather than a formal replication.

### 3.8. Whole-Tissue Validation of ER167 Perturbation in Three Bulk Cohorts

**Figure 6:**
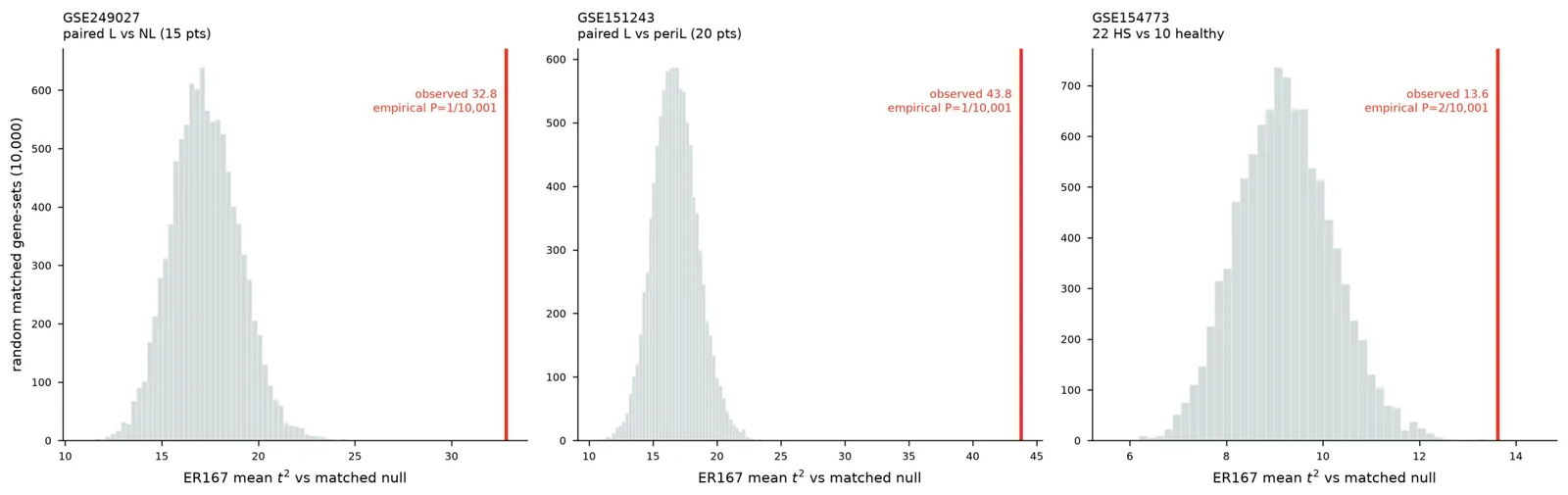
Bulk-tissue validation of ER167 perturbation across three cohorts. The histograms show mean t^2^ statistics from 10,000 expression- and detection-matched random gene sets for GSE249027 (paired lesional versus non-lesional), GSE151243 (paired lesional versus perilesional), and GSE154773 (HS lesional versus healthy skin). Red vertical lines indicate the observed ER167 statistic, which lay at the extreme tail of the matched-null distribution in all three cohorts.

ER167 was significant in all three bulk-tissue comparisons: GSE249027 paired lesional versus non-lesional (162 detected genes; Fry mixed *p* = 8.56 × 10^−8^), GSE151243 paired lesional versus perilesional (164 genes; *p* = 9.75 × 10^−11^), and GSE154773 HS lesional versus healthy skin (164 genes; *p* = 2.39 × 10^−9^). The observed statistic exceeded all 10,000 matched random gene sets in both paired cohorts and all but one in the external cohort (empirical *p* = 1/10,001 and 2/10,001, respectively) (Figure 6; Table S9). Unlike the mixed-direction macrophage result, all bulk comparisons showed a net upward direction, consistent with whole-tissue data capturing both transcriptional changes and increased representation of inflammatory myeloid populations. Most of the established immune and phagocytosis gene sets also exceeded their matched nulls in these cohorts (Section 3.5); the bulk results therefore show that ER167 genes take part in the broad transcriptional shift of lesional tissue and do not indicate specificity.

### 3.9. Bulk Composition Estimates Were Method-Dependent; Adjustment Attenuated but Did Not Abolish the ER167 Signal

The NNLS estimates obtained with the 2000-gene signature (M1) were not reliable. Eighty of the 2000 signature genes were ER167 genes; the 20 most highly expressed signature genes accounted for 96% and 91% of the bulk sum of squares in GSE249027 and GSE151243; the relative residual of the fit averaged 0.81 in lesional and 0.98 in non-lesional samples of GSE249027 (0.76 in lesional and 0.93 in perilesional samples of GSE151243); and the estimated monocyte/macrophage fraction was exactly zero in all 15 non-lesional samples of GSE249027 (Table S14H). When the ER167 genes were removed from the signature (M2), the mean lesional estimate fell from 6.1% to 1.1% in GSE249027 and from 13.9% to 5.9% in GSE151243. In reference mixtures of known composition (true monocyte/macrophage fraction 0.3–7.3%), estimated and true fractions correlated with r = 0.58 for M1, 0.44 for M2, 0.92 for M3 and 0.80 for MuSiC under scheme A, and with r = 0.50, 0.47, 0.91, 0.82 and 0.59 for M1, M2, M3, MuSiC and BisqueRNA under scheme B. Root-mean-square errors were 1.5–5.0 percentage points and were lowest for MuSiC under both schemes (Table S14).

**Figure 7:**
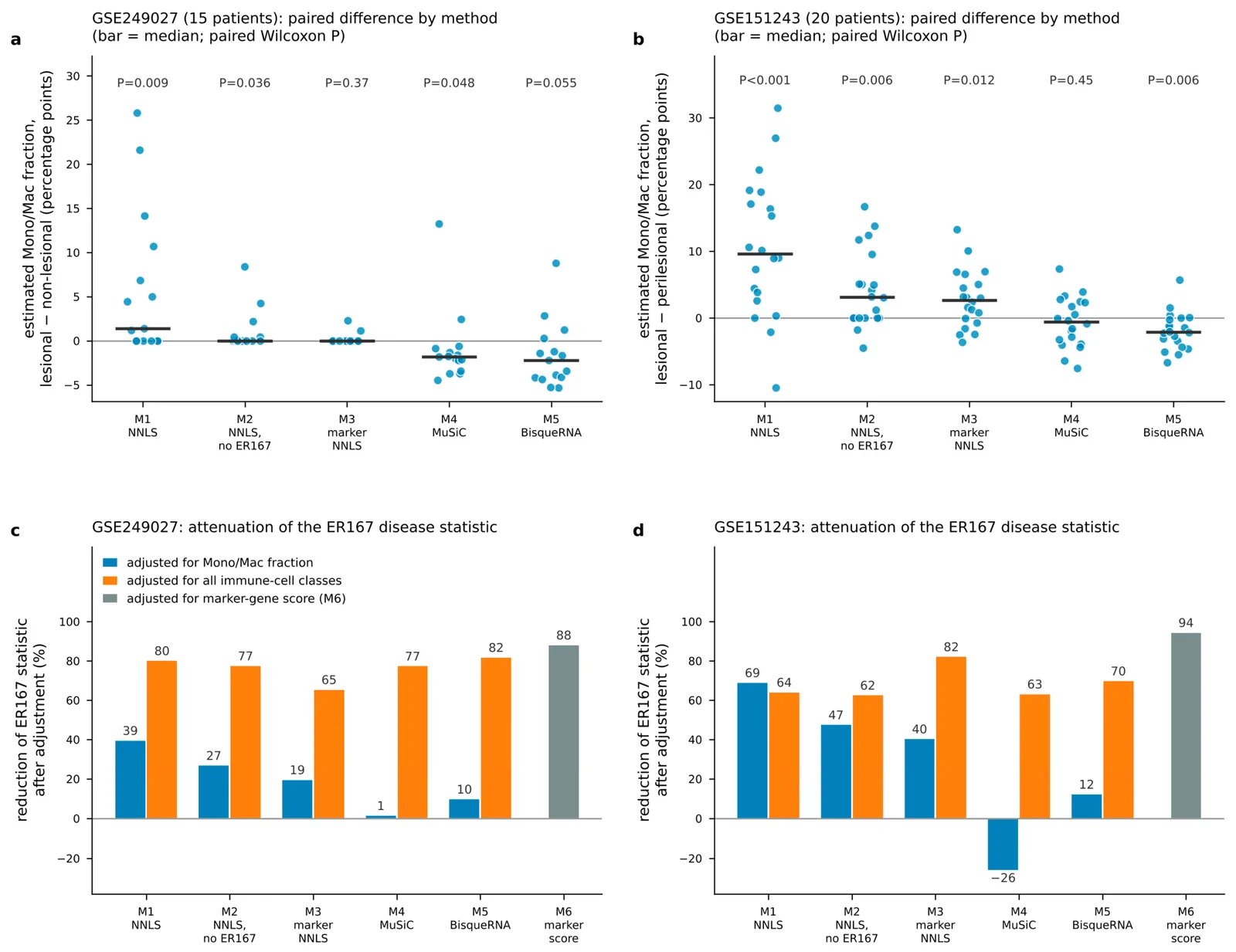
Composition estimates in the paired bulk cohorts and their effect on the ER167 result. Panels (**a**,**b**) show, for each patient, the difference in estimated monocyte/macrophage fraction between lesional and comparator tissue (percentage points) for five deconvolution approaches (M1, non-negative least squares with the 2000-gene signature; M2, the same without ER167 genes; M3, marker-gene non-negative least squares; M4, MuSiC; M5, BisqueRNA) in GSE249027 and GSE151243; horizontal bars are medians. The direction of the difference depends on the method. Panels (**c**,**d**) show the reduction of the ER167 disease statistic (mean squared moderated t) after adjustment for the estimated monocyte/macrophage fraction, for all estimated immune-cell classes, or for a marker-gene score (M6). ER167 remained significant in every adjusted model.

The six estimates did not agree on the direction of the lesional difference (Figure 7a,b; Table S14). NNLS-based fractions were higher in lesional tissue, except for M3 in GSE249027, where the estimate was zero in 28 of the 30 samples (paired Wilcoxon *p* = 0.0092 and 9.0 × 10^−4^ for M1, 0.036 and 0.0057 for M2, and 0.37 and 0.012 for M3, in GSE249027 and GSE151243, respectively), and the marker-gene score (M6) was higher in lesional tissue in both cohorts (*p* = 8.5 × 10^−4^ and 1.9 × 10^−6^). In contrast, the MuSiC and BisqueRNA fractions were lower in lesional tissue or unchanged (MuSiC: median paired difference −1.8 and −0.6 percentage points, *p* = 0.048 and 0.45; BisqueRNA: −2.2 and −2.1 percentage points, *p* = 0.055 and 0.0056). Between-method Spearman correlations of the sample-level estimates ranged from −0.20 to 0.87. With this reference, the monocyte/macrophage content of lesional tissue could therefore not be estimated reliably, and the M1 fractions (mean 6.1% in lesional versus 0.0% in non-lesional tissue in GSE249027, and 13.9% versus 3.3% in GSE151243) should not be interpreted quantitatively.

The association between the ER167 bulk score and the composition estimate was likewise method-dependent (Spearman ρ across all samples from −0.33 to 0.92 in GSE249027 and from −0.16 to 0.95 in GSE151243). Adjustment of the ER167 disease model for the estimated monocyte/macrophage fraction reduced the ER167 statistic by 1% to 39% in GSE249027; in GSE151243 it reduced the statistic by 12% to 69% with four methods and increased it by 26% with MuSiC. Adjustment for all estimated immune-cell classes reduced it by 65–82% and 62–82%, and adjustment for the marker-gene score reduced it by 88% and 94%. ER167 remained significant in every adjusted model (Fry mixed *p* from 2.08 × 10^−7^ to 0.0128 in GSE249027 and from 3.19 × 10^−12^ to 0.0376 in GSE151243; Figure 7c,d; Table S14). These results suggest that most of the bulk ER167 signal reflects the immune-cell content of lesional tissue; a smaller component remained after adjustment, but its size cannot be determined with the available composition estimates.

### 3.10. No ER167 Change Was Detectable at Week 12 of Biologic Treatment

**Figure 8:**
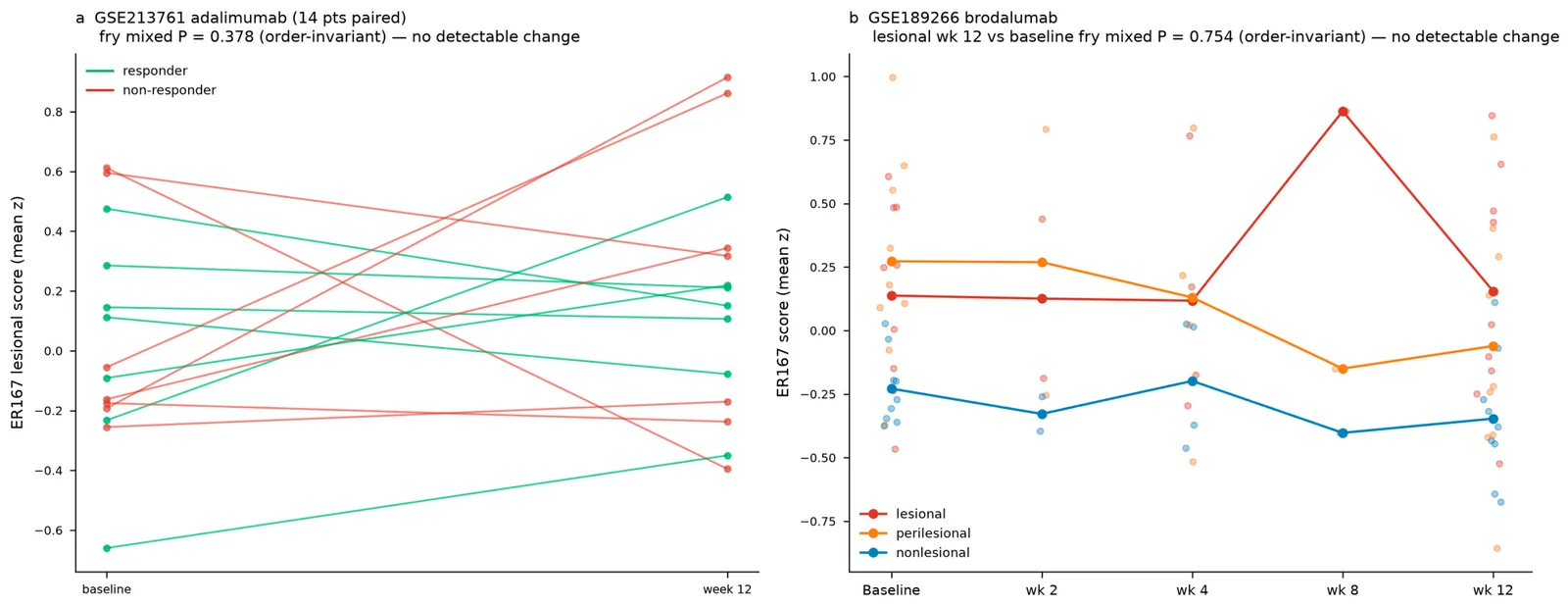
ER167 expression scores during 12 weeks of biologic treatment. Panel (**a**) shows paired lesional baseline and week-12 values for 14 adalimumab-treated patients, stratified descriptively by response status. Panel (**b**) shows brodalumab time-point distributions; because donor linkage was unavailable, the baseline-versus-week-12 contrast was analyzed cross-sectionally. No statistically detectable ER167 change was observed in either cohort at week 12.

In 14 patients with paired lesional samples before and after adalimumab, ER167 did not change significantly at week 12 (Fry mixed *p* = 0.378). The mean change in the lesional ER167 score was 0.14 (95% confidence interval −0.18 to 0.47; Hedges g = 0.24, −0.26 to 0.74), and the smallest change detectable with 80% power was 0.45 score units. The change did not differ between the seven responders and the seven non-responders (difference −0.08; 95% confidence interval −0.78 to 0.63; exploratory). In the 13 patients with all four samples, the lesional–non-lesional difference in ER167 score was 0.08 (−0.28 to 0.45) at baseline and 0.34 (0.09 to 0.60) at week 12. Because the mean score did not separate lesional from non-lesional skin at baseline, it has little capacity to show a contraction of the disease gap. The cross-sectional brodalumab comparison likewise showed no significant ER167 change between nine baseline and nine week-12 lesional samples (*p* = 0.754; difference in mean score 0.02, 95% confidence interval −0.40 to 0.44; Hedges g = 0.04, −0.84 to 0.92; smallest detectable difference 0.59) (Figure 8 and Figure S13; Tables S10 and S18). If treatment reversed the disease-associated program, treatment and disease gene-level vectors would be expected to correlate negatively. The observed correlations were positive for both disease references and both drugs (Spearman ρ = 0.22–0.52), but none differed from the distribution obtained when the sample labels were permuted (two-sided permutation *p* = 0.11–0.70; one-sided *p* for a negative correlation 0.65–0.95). The permutation distributions were wide (central 95% from about −0.59 to 0.59, and from −0.79 to 0.79 for brodalumab against GSE249027), which shows that correlations of this size arise by chance among the correlated genes of the panel. The ER167 correlations were larger than those of expression-matched random gene sets (empirical *p* = 1/10,001 in two comparisons and 0.028 and 0.029 in the other two). These correlations are therefore descriptive: they give no indication of reversal, and they do not establish a positive association (Figure S6; Tables S10 and S18). The available cohorts therefore showed no detectable change in ER167 by week 12. Given the small sample sizes and the cross-sectional brodalumab comparison, this is an absence of detectable change and not evidence that ER167 is unaffected by treatment; smaller, responder-specific, or later changes are not excluded.

### 3.11. Integrated Interpretation

Across discovery macrophages, formal independent scRNA-seq replication, and three bulk validation cohorts, the ER167 panel showed reproducible transcriptional perturbation in HS. The perturbation was not specific to this panel: in discovery macrophages it was shared by two canonical efferocytosis gene sets and by the Hallmark inflammatory-response gene set, and in bulk tissue by most of the immune gene sets examined. Within-state and composition-adjusted analyses were compatible with both a compositional component and a transcriptional component that remained detectable after adjustment for estimated immune-cell composition, although the composition estimates were method-dependent and neither analysis can conclusively separate regulation within macrophages from residual composition effects. These transcriptomic findings do not, without a functional clearance assay, establish impaired, enhanced, or restored efferocytosis.

## 4. Discussion

In this multi-cohort transcriptomic study, we identified a reproducible perturbation of an externally derived efferocytosis-related gene panel in HS lesional macrophages. The principal finding was not a uniform increase or decrease in ER167 expression, but a marked mixed-direction alteration in which inflammatory/myeloid components and selected recognition pathways were increased while several scavenger, recognition, and homeostatic macrophage-associated genes were reduced. This signal exceeded expression- and detection-matched random gene sets and persisted after removal of the panel genes that belong to established inflammatory, interferon, macrophage-activation and phagocytosis gene sets. It was not, however, specific to the panel: two canonical efferocytosis gene sets behaved in the same way, and so did the Hallmark inflammatory-response gene set. Importantly, the finding was independently reproduced in a second scRNA-seq cohort and supported at the whole-tissue level in three bulk-tissue datasets. Together, these observations indicate that efferocytosis-related genes are consistently perturbed in HS lesional macrophages, as part of, and not clearly separable from, the inflammatory remodeling of these cells.

The mixed-direction nature of the ER167 signal is biologically relevant because efferocytosis is not a single transcriptionally uniform process. Recognition of dying cells, receptor engagement, internalization, intracellular processing, metabolic adaptation, and downstream resolution signaling can be regulated independently [7,11]. Single-cell experiments have demonstrated heterogeneous transcriptional responses to apoptotic-cell exposure among macrophage subsets, with homeostatic pathways induced in some populations but suppressed in others [12]. Experimental disruption of chloride sensing can likewise replace the expected anti-inflammatory response with inflammatory and oxidative-stress programs without necessarily preventing corpse degradation, demonstrating that clearance and downstream transcriptional resolution can become uncoupled [13]. These observations provide a mechanistic framework for our finding that the ER167 mixed-direction test was strongly positive despite the absence of a significant net directional effect or a difference in the aggregate donor-level UCell score. Rather than representing contradictory results, these findings suggest that an aggregate unidirectional score may obscure coordinated changes occurring in opposite directions across distinct components of the efferocytosis-related program.

The individual-gene pattern further supports this interpretation. Among the 141 ER167 genes detectable in discovery macrophages, 62 were differentially expressed, with 36 increased and 26 decreased. Representative increases included *SPP1*, *AQP9*, *TLR4*, and *IL1B*, whereas *VSIG4*, *MRC1*, *MARCO*, and *CD36* were decreased. Previous single-cell studies in HS have demonstrated prominent inflammatory macrophage programs, including enhanced phagocytic and antimicrobial functions and STAT1/interferon-associated signaling [3]. *TLR4* and IL-1β are consistent with an inflammatory myeloid context, whereas *MRC1* and *CD36* are commonly associated with alternatively activated or homeostatic macrophage programs [27]. *VSIG4* is an immunoregulatory macrophage receptor. In tumor-associated macrophages, *VSIG4* has been reported to promote, not repress, *SPP1* transcription [28]; the inverse pattern observed here (lower *VSIG4*, higher *SPP1*) is therefore more likely to reflect altered macrophage-state composition than direct regulation of one gene by the other. However, these findings should not be reduced to a binary M1/M2 classification, because in vivo macrophage phenotypes represent multidimensional states that are incompletely captured by classical polarization terminology [27]. This was the rationale for retaining marker-defined macrophage-state labels rather than applying M1/M2 designations in the present study.

Macrophage-state abundance analysis provided a second level of interpretation. HS lesions showed expansion of Mono_FCN1_CCL3 and Mono_FCN1_infl populations together with depletion of Mac_C1QC_MRC1, Mac_HLADR_APC, and Mac_MMP9_SPP1 states. A mass-cytometry study of whole blood has also reported a distinct peripheral immune profile and upregulated CD38 expression in patients with HS [29]. In skin, reduced CD163-expressing regulatory macrophage populations have been described even in clinically non-lesional HS skin, suggesting that altered myeloid homeostasis may not be restricted to fully developed lesions [1]. Our data extend these observations by suggesting that altered cell-state abundance alone may not fully account for the ER167 signal. Mixed-direction ER167 perturbation remained detectable after analysis was restricted to the three macrophage states that could be tested, although only the MonoMac_hypoxic_lipid result was stable across cell-count thresholds and after removal of individual donors; four of the seven states could not be tested at all, because they were nearly absent from one of the groups. Thus, the overall disease-associated pattern appears to contain both a compositional component, reflecting altered representation of macrophage states, and a within-state transcriptional component. Because the states were broadly defined and each within-state test rested on nine donors, residual heterogeneity within a state cannot be excluded, and these analyses should be regarded as exploratory.

This distinction is also supported by the bulk-transcriptomic analyses. ER167 was strongly perturbed in paired lesional versus non-lesional tissue, paired lesional versus perilesional tissue, and an external lesional-versus-healthy cohort. In contrast to the mixed-direction macrophage-specific result, bulk comparisons showed a net upward direction. Cellular composition is the most likely explanation for much of this discrepancy. A marker-gene score of monocyte/macrophage content was higher in lesional tissue in both paired cohorts, and adjustment for all estimated immune-cell classes reduced the ER167 disease statistic by 62–82%. The cell-fraction estimates themselves were not reliable: the single-cell reference contained essentially no keratinocytes and few monocytes/macrophages, and the deconvolution approaches disagreed on whether the monocyte/macrophage fraction was higher or lower in lesional tissue. The adjusted gene-set effects remained significant in every model. Bulk-tissue ER167 elevation should therefore not be interpreted as increased pathway activity within individual macrophages. It probably reflects mainly the immune-cell content of lesional tissue, together with a residual component whose size could not be determined. This interpretation is consistent with experimental evidence that macrophage subpopulations can display divergent efferocytosis-associated transcriptional responses that become obscured when measured at aggregate tissue level [12].

The replication structure strengthens the inference that the observed ER167 perturbation was not specific to a single cohort or preprocessing strategy. The formal independent scRNA-seq replication cohort reproduced the mixed-direction gene-set signal, and ER167 gene-level effect sizes were positively correlated with discovery, with substantially higher directional concordance among genes that were significant in the discovery cohort. By contrast, GSE220116 was intentionally treated as descriptive because only one eligible healthy-control donor remained after macrophage-count filtering. This distinction is important given the susceptibility of small-donor scRNA-seq comparisons to unstable donor-level inference. The concordant results across independent single-cell and bulk modalities therefore provide stronger evidence for reproducible efferocytosis-related transcriptional remodeling than any individual cohort alone.

The specificity analyses qualify the interpretation of the primary result. In discovery macrophages, the Hallmark inflammatory-response gene set was as extreme relative to its matched null as ER167, so that an efferocytosis-related panel cannot be distinguished from general inflammation on this criterion. HS lesions are characterized by extensive innate immune activation, interferon signaling, neutrophilic inflammation, and inflammatory cytokine production [2,3,4], and a gene set rich in myeloid and inflammatory genes would be expected to be perturbed in such tissue. Three observations nevertheless indicate that the ER167 result is not explained by its inflammatory genes alone. First, ER167 continued to exceed its matched null after removal of the genes annotated to inflammatory, interferon, macrophage-activation, phagocytosis, phagosome and lysosome gene sets. Second, the Gene Ontology apoptotic-cell-clearance and KEGG efferocytosis gene sets, which contain the canonical recognition and engulfment machinery that is largely absent from ER167, also exceeded their matched nulls. Third, the result was not a property of every immune gene set: the interferon-response, Fcγ-receptor phagocytosis, phagosome-maturation and lysosome gene sets did not exceed their matched nulls in macrophages. In bulk tissue, by contrast, most gene sets exceeded their matched nulls, and no specificity can be claimed at whole-tissue level. These analyses do not establish efferocytosis-specific dysfunction; they indicate that efferocytosis-related genes, whether defined by the adopted panel or by curated pathway databases, were among the more strongly perturbed of the gene sets examined in HS lesional macrophages.

The longitudinal analyses yielded a different result. No statistically detectable change in ER167 was observed at week 12 of adalimumab or brodalumab treatment, and the correlations between treatment-associated and disease-associated ER167 gene-level vectors gave no indication of reversal, although they could not be distinguished from chance when sample labels were permuted. These observations should not be interpreted as evidence that the therapies were biologically ineffective, nor as evidence that ER167 is unaffected by treatment, because both treatment cohorts were small and the brodalumab comparison was cross-sectional. Molecular studies of HS have repeatedly demonstrated that clinical improvement can occur without complete normalization of the lesional transcriptome. Adalimumab has been reported to strongly attenuate selected B-cell-associated signals while leaving several other inflammatory pathways relatively preserved [1]. Similarly, IL-17RA blockade with brodalumab reduced inflammatory, keratinocyte, and neutrophil-associated pathways, but transcriptomic normalization was incomplete and less pronounced in established lesional than in perilesional tissue [26]. Guselkumab treatment has likewise shown clinical responses without a consistent correspondence between clinical improvement and molecular normalization [30]. More recently, the magnitude of neutrophil-associated transcriptional suppression during IL-17A/IL-17F blockade has been reported to vary according to clinical response [31]. Thus, the absence of a detectable ER167 change at 12 weeks is compatible with the broader observation that suppression of a therapeutically targeted inflammatory axis does not necessarily restore all disease-associated transcriptional programs. Twelve weeks may also be too early to observe remodeling of the myeloid compartment of established lesions. The absence of a detectable change therefore has at least three explanations that these data cannot separate: limited statistical power (changes smaller than about 0.45 score units in the paired adalimumab cohort and 0.59 in the brodalumab cohort would probably not have been detected), a change that occurs later than week 12, or a tissue state that does not respond to cytokine blockade. Because the treatment cohorts were bulk tissue, in which the ER167 signal was largely attributable to immune-cell content in the disease comparisons, a persisting signal would indicate persisting infiltration and could not be read as persistent efferocytic dysfunction. Whether a treatment-refractory component exists, and whether it would respond to interventions that target resolution pathways, is a hypothesis that requires larger paired cohorts sampled beyond week 12, with cell-resolved and functional readouts.

Several aspects of the study design strengthen these findings. The efferocytosis-related panel was taken from an independent publication on a different disease and applied without modification, so that panel membership could not be influenced by HS-associated results. The 167-gene panel was adopted directly from the published supplementary efferocytosis-related gene (EFRG) list and subsequently harmonized to current HGNC nomenclature without altering panel membership [18]. Discovery inference was conducted at the donor rather than cell level, independent datasets were not merged to generate a common inferential statistic, and replication and validation cohorts were analyzed in separate, non-overlapping roles. The primary result also remained stable after inclusion of the low-cell-count donor, with raw versus decontaminated counts, and with an alternative matched-null statistic. Because a discovery cohort of four HS and five control donors is vulnerable to the influence of single donors, the complete analysis was also repeated after removal of each donor in turn; the mixed-direction result and its matched-null significance were retained in all nine re-analyses, and gene-level log2 fold changes correlated with those of the full analysis at r = 0.944–0.997. This leave-one-donor-out analysis assesses the internal stability of the estimate and does not replace external replication, which was provided by the independent scRNA-seq cohort. Collectively, these features reduce the likelihood that the primary observation reflects pseudoreplication, dependence on one donor, a single quality-control decision, or post-hoc gene selection.

The study also has important limitations. First, ER167 was taken from the supplementary gene list of a melanoma transcriptomic study and was not developed for HS biology [18]. An identical list had been published earlier as a supplementary table of an endometrial cancer study [32]; both publications describe the list as compiled from published literature without describing the selection procedure, so the origin of the list and the reason for the inclusion of individual genes cannot be traced. Only 17 of its 167 genes belong to the Gene Ontology apoptotic-cell-clearance or KEGG efferocytosis gene sets; its functional categories were assigned manually and are poorly supported by Gene Ontology annotation, and it contains many myeloid and inflammatory genes. The panel should therefore be regarded as an efferocytosis-associated macrophage gene list and not as a validated measure of the efferocytosis pathway. The analyses with canonical gene sets mitigate, but cannot eliminate, this limitation, and the primary result was not specific to the panel. In addition, the analysis plan was not registered in a public registry before the analyses were performed. Second, the discovery and replication scRNA-seq cohorts contained relatively small numbers of donors, and one candidate replication dataset was unsuitable for formal inference because only one control donor remained after cell-count gating. In pseudobulk analysis, the variance of well-expressed genes is determined mainly by the variability between donors (biological coefficient of variation 0.42 in the discovery data), which nine donors estimate imprecisely. In simulations, the discovery design reached about 80% power only when at least a quarter of the panel genes changed by 1 log2 unit or at least a tenth changed by 1.5 log2 units; a three-versus-three within-state comparison had lower power; changes of 0.5 log2 units had power below 0.5 in both designs; and individual genes with changes below about 1.5 log2 units would usually not have reached significance. Two control donors contributed 64% of the monocyte/macrophage cells, although removal of any single donor did not change the result. Donor age, sex, anatomical site, disease stage and treatment are not reported in the GEO record of the discovery cohort and could not be examined as confounders; in the replication cohort, the HS samples were surgical excision tissue whereas the control samples were mammoplasty skin, so that disease status is confounded with anatomical site and tissue source. Third, bulk composition was estimated and not measured. The single-cell reference was dominated by lymphocytes, contained essentially no keratinocytes and few monocytes/macrophages, and carried imperfect class labels, and the deconvolution methods disagreed on the direction of the lesional difference in monocyte/macrophage fraction; attenuation after adjustment should therefore be interpreted as evidence of a compositional contribution, and neither as a quantification of that contribution nor as proof of a macrophage-independent residual effect. Fourth, both treatment cohorts were small, and the brodalumab baseline-to-week-12 analysis was cross-sectional because reliable donor linkage was unavailable, which limits longitudinal inference. Finally, and most importantly, transcript abundance does not directly measure uptake, engulfment kinetics, phagolysosomal processing, or continual efferocytic capacity. Gene expression and functional clearance can correspond under some experimental conditions but can also become dissociated because of macrophage heterogeneity, cellular cargo, or saturation of clearance capacity [12,14,15]. Accordingly, the present data support efferocytosis-related transcriptional dysregulation, but not a direct demonstration of defective efferocytosis. In addition, donor sex was not consistently reported in the metadata of the analyzed public datasets and was therefore not used as a covariate; sex- and gender-disaggregated analyses could not be performed, and the findings should not be assumed to apply equally across sexes.

This distinction defines the most relevant next step. Functional studies should directly quantify apoptotic-cell recognition and uptake in HS-derived macrophages, ideally combined with assessment of sequential efferocytosis, phagolysosomal processing, and resolution-associated mediator production. Integration with spatial transcriptomic or imaging approaches could further determine whether the ER167 states identified here localize preferentially around tunnels, neutrophil-rich areas, apoptotic keratinocytes, or other regions of active tissue injury.

## 5. Conclusions

In conclusion, our findings identify a reproducible mixed-direction perturbation of efferocytosis-related genes in HS lesional macrophages. The perturbation was not specific to the adopted panel: it was also seen for canonical efferocytosis gene sets and was matched by the general inflammatory-response gene set. The signal is characterized by coordinated inflammatory and homeostatic alterations, is compatible with contributions from both macrophage-state redistribution and within-state transcriptional change, replicates in an independent scRNA-seq cohort, and is supported at whole-tissue level in three bulk-tissue datasets. Adjustment for estimated immune-cell composition attenuated most of the bulk signal, which suggests a large compositional contribution. No ER167 change was detectable at week 12 of adalimumab or brodalumab treatment. This observation rests on a single post-treatment time point in small cohorts, and the brodalumab comparison was cross-sectional; it shows the absence of a detectable change at that time point, not refractoriness of the efferocytosis-related program to these therapies. Whether any component of the signal persists beyond week 12 requires larger, longitudinally sampled cohorts with cell-resolved readouts. These findings position efferocytosis-related molecular programming as a previously insufficiently characterized dimension of HS myeloid biology, while functional studies remain necessary to determine its consequences for apoptotic-cell clearance and inflammation resolution.

## Figures and Tables

**Table 1:** Analyzed cohorts, inferential roles, and principal ER167 gene-set results.

Dataset	Role and Design	Sample Structure	Fry Mixed *p*	Matched-Null *p*	Interpretation
GSE249793	Discovery scRNA; HS vs. HC macrophage	4 HS/5 HC	8.56 × 10^−7^	1/10,001	Primary endpoint met
GSE212721	Formal scRNA replication	6 HS/3 HC	0.0278	0.0296	Independent replication
GSE220116	Descriptive scRNA replication	5 HS/1 HC	0.0065	0.122	Descriptive only
GSE249027	Paired bulk; lesional vs. non-lesional	15 paired patients	8.56 × 10^−8^	1/10,001	Whole-tissue validation
GSE151243	Paired bulk; lesional vs. perilesional	20 paired patients	9.75 × 10^−11^	1/10,001	Whole-tissue validation
GSE154773	External bulk; HS vs. healthy	22 HS/10 HC	2.39 × 10^−9^	2/10,001	Whole-tissue validation
GSE213761	Adalimumab; baseline vs. week 12	14 paired patients	0.378	—	No detectable change
GSE189266	Brodalumab; baseline vs. week 12	9 vs. 9 samples	0.754	—	No detectable change

ER167, 167-gene efferocytosis-related panel; HC, healthy control; HS, hidradenitis suppurativa; scRNA, single-cell RNA sequencing. GSE220116 contained one eligible control and was not treated as a formal replication. Fry mixed *p*-values are from the order-invariant test (Section 2.7), except for GSE212721 and GSE220116, for which the weighted test in the original sample order is shown. Matched-null *p*-values are empirical values from 10,000 matched random gene sets (minimum attainable value 1/10,001). Bulk cohorts provide whole-tissue validation and not macrophage-specific replication.

**Table 2:** Representative ER167 genes differentially expressed in discovery macrophage pseudobulk (GSE249793).

Gene	ER167 Category	log_2_ Fold-Change	FDR	Direction in HS
*SPP1*	Inflammatory/myeloid context	4.67	0.0139	Increased
*AQP9*	Inflammatory/myeloid context	3.54	2.24 × 10^−4^	Increased
*TLR4*	Recognition/tethering	3.40	2.02 × 10^−4^	Increased
*IL1B*	Inflammatory/myeloid context	2.08	0.00815	Increased
*VSIG4*	Recognition/tethering	−5.57	0.00185	Decreased
*MRC1*	Recognition/tethering	−3.07	0.00334	Decreased
*MARCO*	Recognition/tethering	−2.87	0.00625	Decreased
*CD36*	Recognition/tethering	−2.61	0.00957	Decreased

ER167, 167-gene efferocytosis-related panel; FDR, false discovery rate; HS, hidradenitis suppurativa. Positive log_2_ fold-change indicates higher expression in HS lesional macrophages. FDR was controlled by the Benjamini-Hochberg method.

## Data Availability

The data presented in this study are publicly available in the Gene Expression Omnibus (GEO) at https://www.ncbi.nlm.nih.gov/geo/ (accessed on 20 August 2026), under accession numbers GSE249793, GSE212721, GSE220116, GSE274880, GSE249027, GSE151243, GSE154773, GSE213761, and GSE189266. All derived results supporting the conclusions of this article are included in the article and its Supplementary Materials. The analysis scripts, configuration, metadata and environment files are provided in Supplementary File S1.

## Supplementary Materials

The following supporting information can be downloaded at: https://www.mdpi.com/article/10.3390/biomedicines14102322/s1, Figure S1: Per-cell discovery scRNA-seq quality-control distributions; Figure S2: Estimated ambient-RNA contamination by discovery donor; Figure S3: Mitochondrial-read threshold sensitivity; Figure S4: Marker profiles supporting myeloid-state annotation; Figure S5: Gene-level concordance of ER167 effects between discovery and replication; Figure S6: Disease-treatment ER167 vector correlations and their permutation distributions; Figure S7: Dependence of the weighted Fry *p* value on sample order, and the order-invariant values reported in the manuscript; Figure S8: Donor-level structure of the discovery monocyte/macrophage pseudobulk profiles; Figure S9: Donor-level expression of the detected ER167 genes in discovery; Figure S10: ER167 and established gene sets in the discovery matched-null comparison; Figure S11: Within-state ER167 tests at increasing minimum numbers of cells per donor; Figure S12: Simulated power of the discovery design; Figure S13: Lesional ER167 score before and after 12 weeks of adalimumab; Table S1: ER167 panel, HGNC harmonization, stable identifiers, and manually assigned functional categories; Table S2: Stage-0 GEO dataset inventory and cohort-level metadata summary; Table S3A: Go/no-go assessment for single-cell discovery, replication, and reference cohorts; Table S3B: ER167 annotation, symbol mapping, and detection coverage across candidate cohorts; Table S4: Per-donor discovery scRNA-seq quality-control metrics and mitochondrial-threshold sensitivity; Table S5A: Discovery macrophage pseudobulk differential-expression results for detected ER167 genes; Table S5B: Per-donor complementary ER167 UCell expression-score summaries; Table S6A: Primary ER167 gene-set and competitive matched-null results; Table S6B: Functional-category-resolved matched-null results; Table S6C: Primary endpoint sensitivity to donor gates, count source, and matched-null statistic; Table S6D: Specificity of ER167 relative to general-inflammation and myeloid-activation panels; Table S7A: Donor-level monocyte/macrophage-state abundance analysis; Table S7B: Within-state mixed-direction ER167 analyses; Table S8A: Independent scRNA-seq replication and descriptive-cohort summary; Table S8B: Gene-level ER167 concordance between discovery and GSE212721; Table S8C: ER167 gene-level effects in discovery and formal scRNA-seq replication; Table S9A: Bulk-transcriptomic ER167 validation across paired and external cohorts; Table S9B: ER167 gene-level effects across the three bulk validation cohorts; Table S10A: Associations between ER167 bulk scores and estimated monocyte/macrophage fractions; Table S10B: Attenuation of the ER167 disease statistic after composition adjustment; Table S10C: Twelve-week adalimumab and brodalumab ER167 gene-set results; Table S10D: Adalimumab longitudinal sample metadata and ER167 scores (GSE213761); Table S10E: Brodalumab sample metadata and ER167 scores by time point (GSE189266); Table S10F: Disease-treatment ER167 vector correlations across both disease references and both biologics; Table S11: Order dependence of the weighted Fry test and order-invariant values for every reported Fry result; Table S12A: ER167 and established gene sets: Fry and matched-null results in the discovery and bulk cohorts; Table S12B: ER167 after exclusion of manually assigned functional categories; Table S12C: ER167 after removal of genes shared with established gene sets; Table S12D: Definitions and sources of the gene sets compared with ER167; Table S13A: Gene Ontology and KEGG annotation of the 167 panel genes; Table S13B: Agreement between the manually assigned categories and ontology annotation; Table S13C: Number of genes of each manual category annotated to each ontology term; Table S14A: Composition of the single-cell reference used for deconvolution (GSE274880); Table S14B: Most specific genes and canonical markers of the reference classes; Table S14C: Validation of the composition estimators on pseudobulk mixtures of known composition; Table S14D: Estimated monocyte/macrophage content by method and tissue in the paired bulk cohorts; Table S14E: Spearman correlation between the bulk ER167 score and the estimated monocyte/macrophage content; Table S14F: Attenuation of the ER167 disease statistic after composition adjustment, by method; Table S14G: Agreement between the six estimates of monocyte/macrophage content (Spearman correlation across samples); Table S14H: Diagnostics of the non-negative least squares fit with the 2000-gene signature (method M1); Table S15A: Donor-level metadata available in GEO for the discovery and replication single-cell cohorts; Table S15B: Leave-one-donor-out analysis of the primary ER167 endpoint; Table S15C: Association of the first four principal components with group, library size and cell number; Table S15D: Covariates available for the discovery cohort and covariate-adjusted ER167 result; Table S15E: Tagwise negative-binomial dispersion in the discovery pseudobulk analysis; Table S15F: Leave-one-out projection of the ER167 profile of each discovery donor; Table S16A: Monocyte/macrophage cells per donor and state in the discovery cohort; Table S16B: Within-state ER167 tests by minimum number of cells per donor; Table S16C: Leave-one-donor-out within-state ER167 tests (minimum of 10 cells per donor); Table S17A: Simulated rejection rate of the mixed-direction Fry test for a gene set of the size of ER167; Table S17B: Simulated gene-level power of the discovery design (4 HS vs 5 HC; edgeR quasi-likelihood test; FDR < 0.05); Table S18A: Effect sizes for the change in ER167 score after 12 weeks of adalimumab (GSE213761); Table S18B: Change in lesional ER167 score by responder status (GSE213761; exploratory); Table S18C: Effect size for the difference in lesional ER167 score after 12 weeks of brodalumab (GSE189266); Table S18D: Disease-treatment ER167 vector correlations evaluated against permutation of sample labels and against random gene sets; File S1: Analysis code archive (scripts of the original and revision analyses, configuration file with thresholds and random seeds, panel file, GEO sample metadata, gene-set definitions, result tables of the revision analyses, deconvolution signature matrix, environment files and verification record). The publications cited in the Supplementary Materials are included in the main reference list [18,25,32].

## Abbreviations

The following abbreviations are used in this manuscript:

Abbreviation	Definition
EFRG	efferocytosis-related gene
ER167	167-gene efferocytosis-related panel
FDR	false discovery rate
GEO	Gene Expression Omnibus
HC	healthy control
HGNC	HUGO Gene Nomenclature Committee
HS	hidradenitis suppurativa
NET	neutrophil extracellular trap
ROAST	rotation gene set testing
scRNA-seq	single-cell RNA sequencing
UMAP	uniform manifold approximation and projection
