# Supplementary material for: Efferocytosis-Related Transcriptional Dysregulation in Hidradenitis Suppurativa Lesional Macrophages: A Multi-Cohort Single-Cell and Bulk Transcriptomic Study
Source: Biomedicines. 2026 Oct 13;14(10):2322. doi: 10.3390/biomedicines14102322 (PMC13701511; doi:10.3390/biomedicines14102322)
Supplement: Supplementary file 1 [file biomedicines-14-02322-s001.zip › HS_ER167_supplementary.pdf]

Supplementary Material

Efferocytosis-related transcriptional dysregulation in hidradenitis suppurativa lesional macrophages: a multi-cohort single-cell and bulk transcriptomic study

Compilation scope and interpretation

This document compiles thirteen Supplementary Figures (S1-S13) and Supplementary Tables S1-S18. Numbering and terminology follow the main text. ER167 denotes an externally derived efferocytosis-associated gene panel applied without modification; these transcriptomic results do not directly measure efferocytic function.

The tables retain all cohort-level summaries, all ER167 gene-level rows, all inferential statistics cited in the manuscript, and all longitudinal sample-level results. The analysis code is provided separately as Supplementary File S1.

Main-text cross-reference map

Main-text topic	Supporting supplementary items
ER167 panel and Stage-0 cohort audit	Tables S1-S3
Discovery scRNA-seq quality control and state annotation	Figures S1, S2, S3, and S4; Tables S4-S5
Primary endpoint, functional categories, specificity, and sensitivity	Figures S7-S10 and S12; Tables S6, S11-S13, S15, and S17
Macrophage-state abundance and within-state transcriptional component	Figure S11; Tables S7A-S7B and S16
Independent scRNA-seq replication and gene-level concordance	Figure S5; Tables S8A-S8C
Bulk validation, deconvolution, and attenuation after composition adjustment	Tables S9A-S10B and S14
Treatment-associated change and disease-treatment vector analysis	Figures S6 and S13; Tables S10C-F and S18

Supplementary Figures

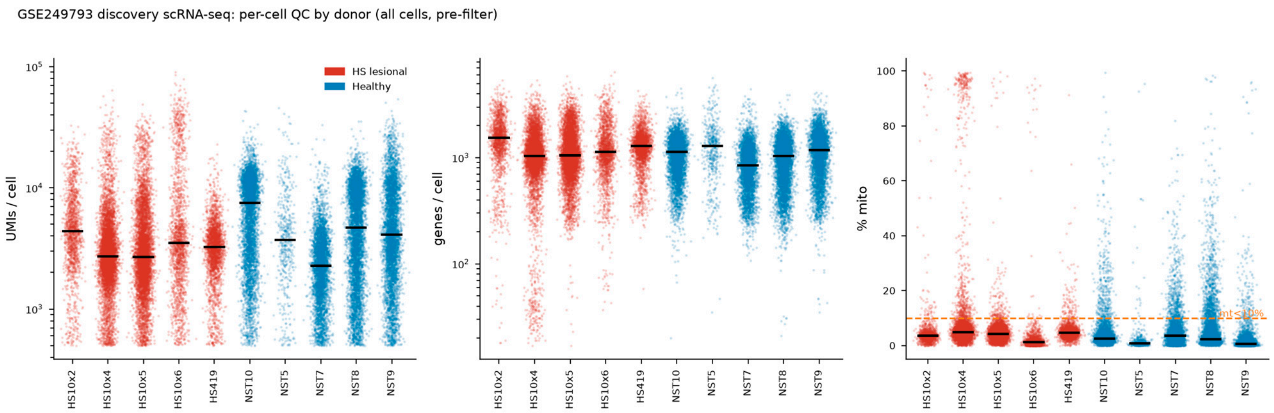

Figure S1. Per-cell discovery scRNA-seq quality-control distributions. Pre-filter unique molecular identifier counts, detected-gene counts, and mitochondrial-read percentages are shown by donor for HS lesional and healthy-control skin. Black bars denote donor medians; the dashed line in the mitochondrial panel marks the 10% threshold used in the primary analysis.
Main-text linkage: Discovery scRNA-seq preprocessing and quality control; Table S4; Results atlas description.

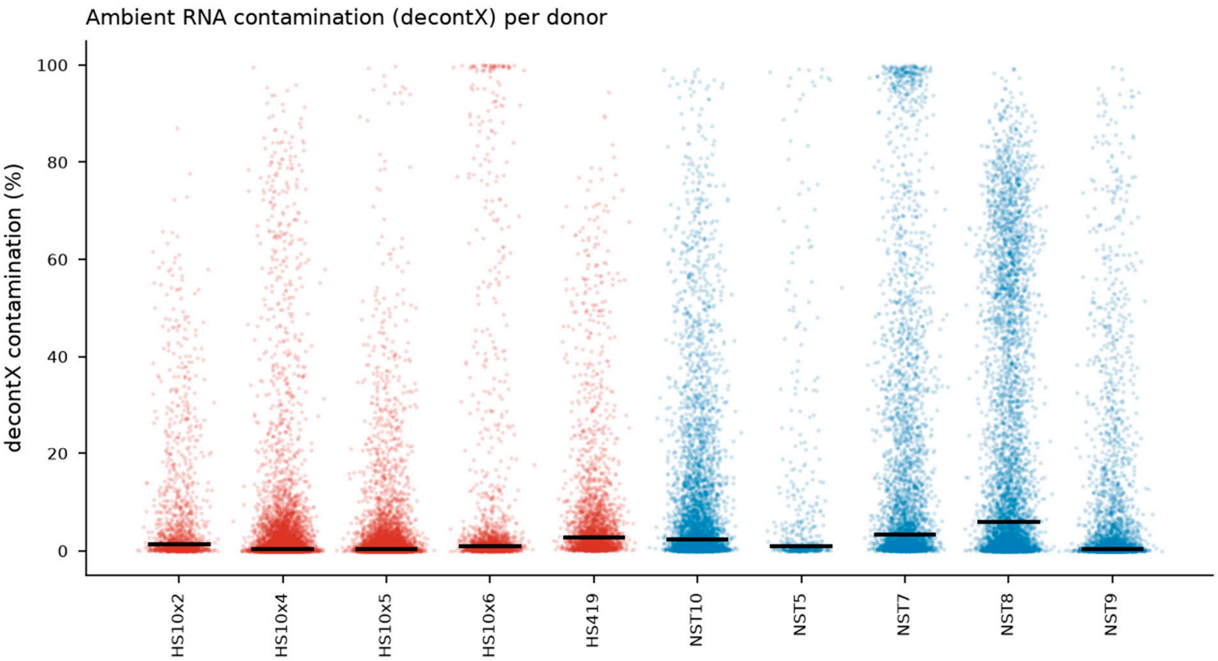

Figure S2. Estimated ambient-RNA contamination by discovery donor. Per-cell decontX contamination estimates are displayed before correction. Black bars denote donor medians. Ambient correction was used for preprocessing and annotation, whereas raw pre-decontX counts were retained for the primary donor-level differential-expression model.

Main-text linkage: Discovery preprocessing and the raw-versus-decontX sensitivity analysis; Table S4 and Table S6C.

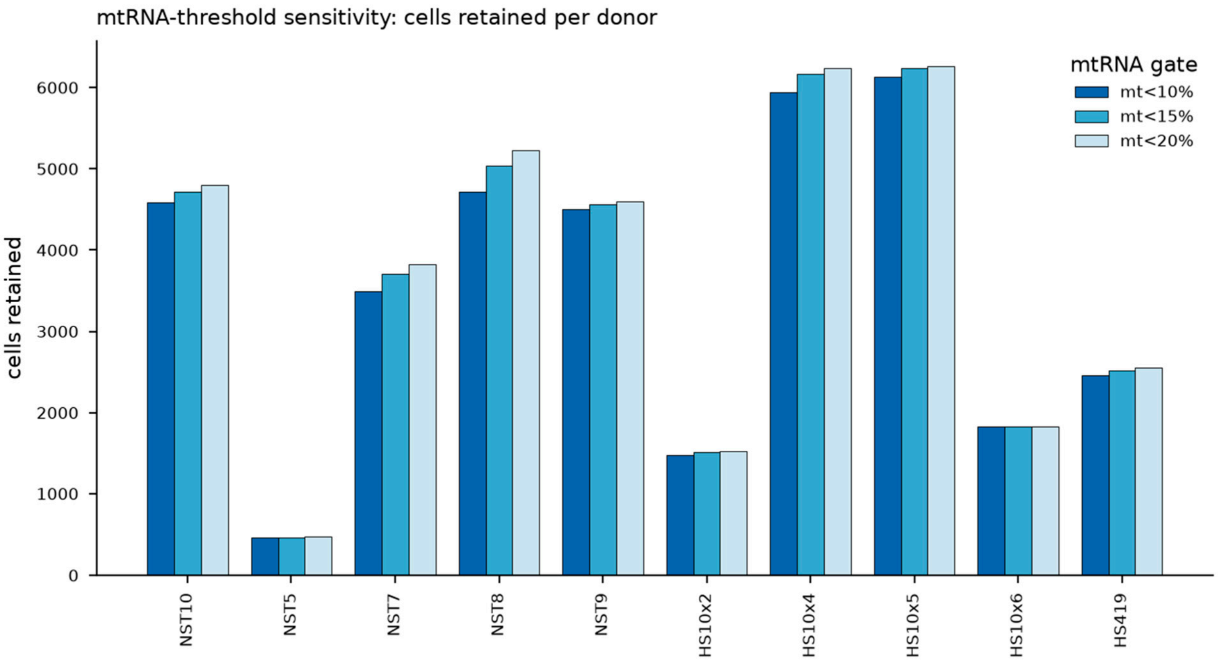

Figure S3. Mitochondrial-read threshold sensitivity. Cells retained per donor are shown under mitochondrial-read thresholds of <10%, <15%, and <20%. The 10% threshold was used for the primary analysis; the alternative gates quantify the effect of less stringent cell-level filtering.

Main-text linkage: Discovery QC sensitivity; Table S4.

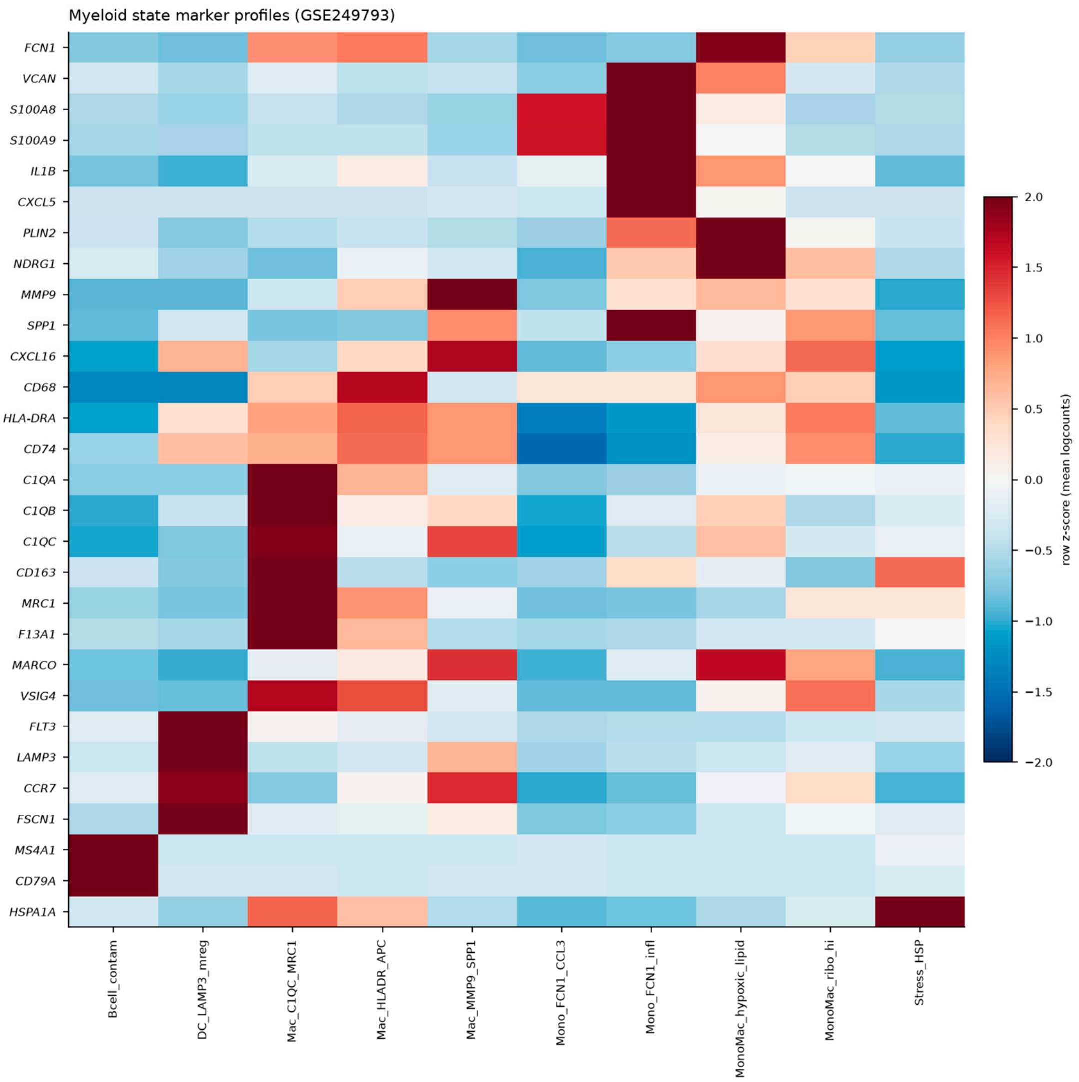

Figure S4. Marker profiles supporting myeloid-state annotation. Rows show z-standardized mean log-expression of canonical markers across re-clustered myeloid states. The profiles support conservative marker-based state labels and the separation of migratory dendritic, B-cell-contaminated, and heat-shock/stress populations from the monocyte/macrophage compartment; no M1/M2 labels were applied.

Main-text linkage: Lineage annotation, macrophage subclustering, and the discovery atlas in Figure 2.

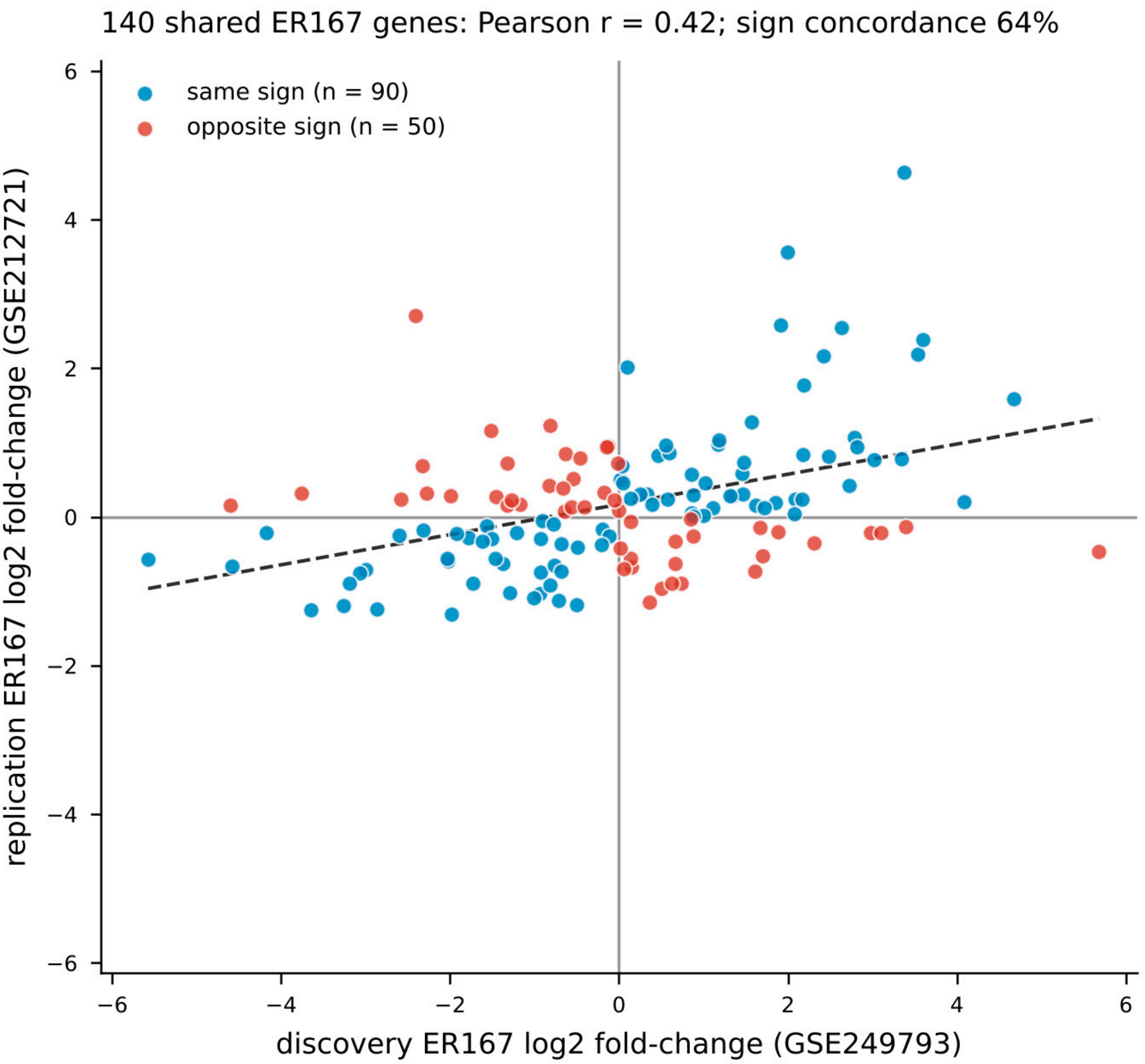

Figure S5. Gene-level concordance of ER167 effects between discovery and replication. ER167 log2 fold-changes (HS versus control) in the GSE212721 replication pseudobulk are plotted against the discovery estimates (GSE249793) for the 140 ER167 genes detected in both cohorts. Blue points have the same sign in both cohorts (n=90) and red points opposite signs (n=50); the dashed line is the least-squares fit (Pearson r=0.42; sign concordance 64%).

Main-text linkage: Independent replication Results; Tables S8A-S8C.

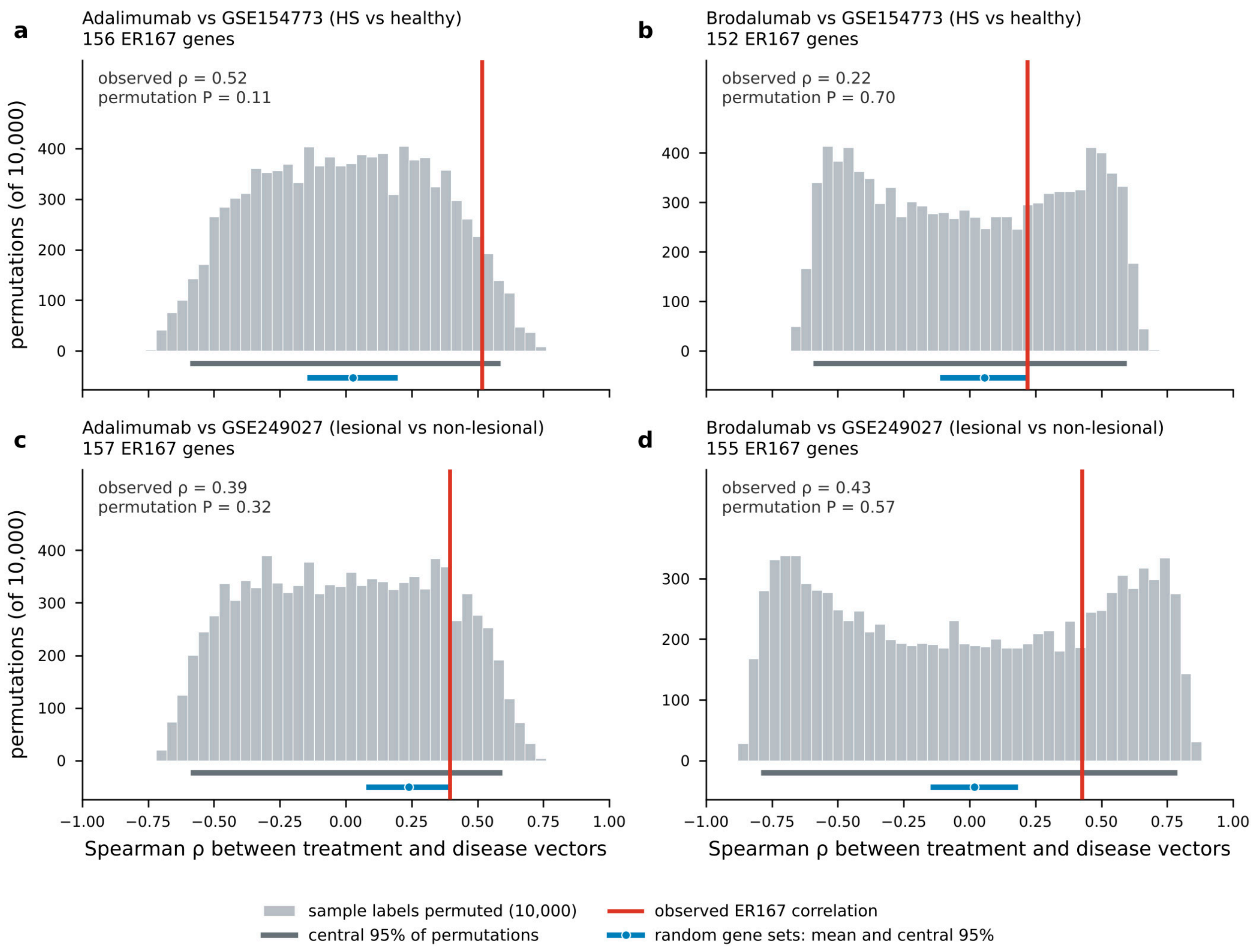

Figure S6. Disease-treatment ER167 vector correlations and their permutation distributions. Each panel shows, for one drug and one disease reference, the Spearman correlation between the treatment-associated and the disease-associated ER167 gene-level log2 fold-changes (red line) against the distribution of the same correlation over 10,000 permutations of the sample labels (grey histogram; dark grey bar, central 95%). For adalimumab the baseline and week-12 labels were exchanged within patients; for brodalumab the time-point labels were permuted among the 18 lesional samples; the treatment model was refitted in each permutation and the disease vector was kept fixed. The blue bar shows the mean and central 95% of the correlations of 10,000 random gene sets of the same size matched for decile of mean expression. Reversal of the disease-associated pattern would give a negative correlation. The observed correlations were positive but lay within the permutation distributions in all four comparisons (two-sided permutation P=0.11–0.70), so they are descriptive. Conventional gene-level P values are not shown, because the genes of the panel are not independent.

Main-text linkage: Methods 2.12; treatment Results; Tables S10F and S18D.

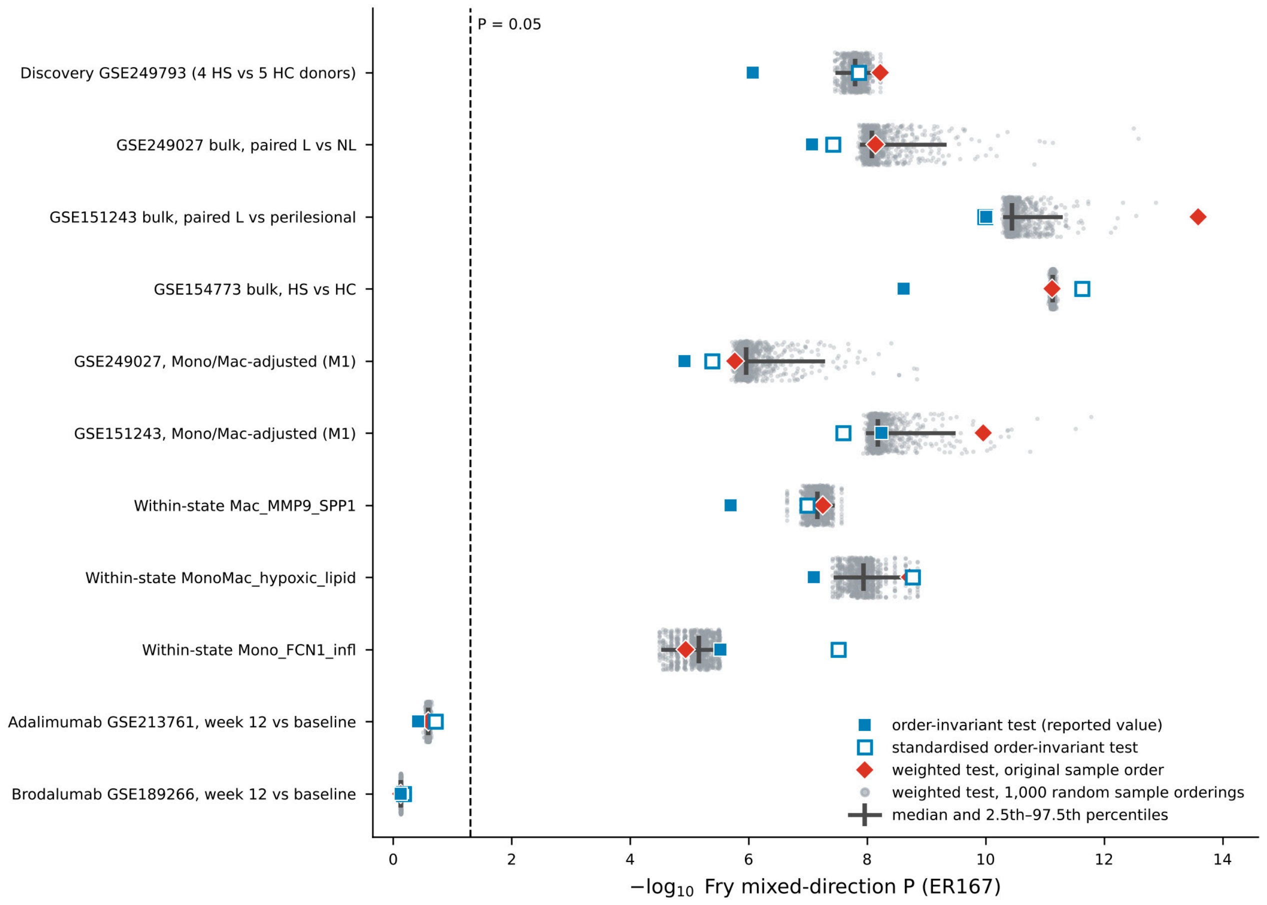

Figure S7. Dependence of the weighted Fry P value on sample order, and the order-invariant values reported in the manuscript. For each ER167 test, grey points show the mixed-direction P values of the default weighted Fry test over 1000 random orderings of the samples (vertical tick and bar: median and 2.5th–97.5th percentiles), and red diamonds show the same test in the original sample order. Filled blue squares show the order-invariant test reported in the main text (voom log2 counts per million, observation weights removed, standardize="none"), and open blue squares the standardised order-invariant test, in which each gene is first divided by its residual standard deviation. The dashed line marks P=0.05. In all eleven analyses, every specification and every sample ordering gave the same conclusion at the 0.05 level. The composition-adjusted rows use the monocyte/macrophage estimate M1. L, lesional; NL, non-lesional; HC, healthy control.

Main-text linkage: Methods 2.7; all Fry results; Table S11.

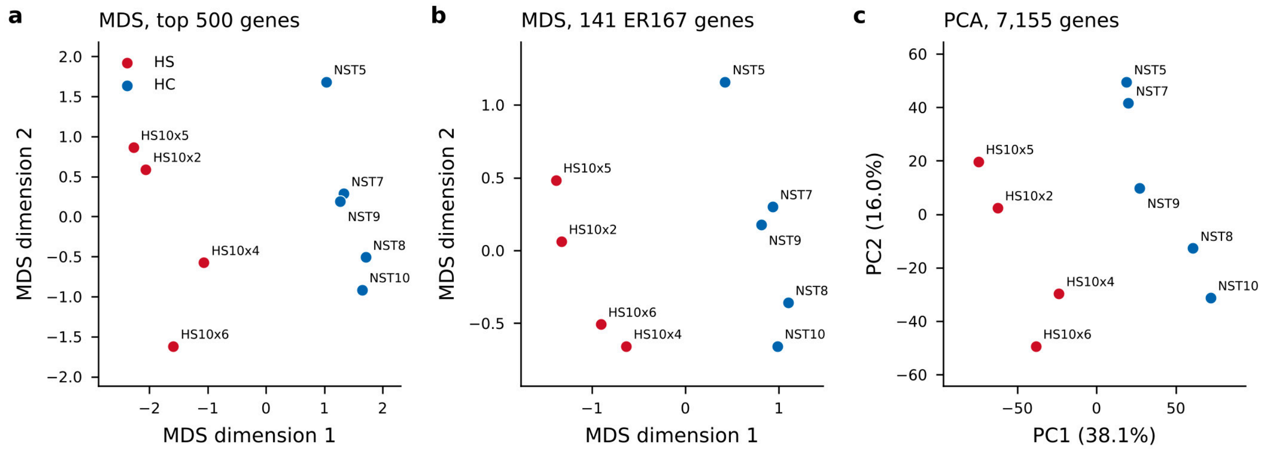

Figure S8. Donor-level structure of the discovery monocyte/macrophage pseudobulk profiles. (a) Multidimensional scaling (limma plotMDS; leading log2 fold-change over the 500 most variable genes of each pair of donors) of the nine donors of the primary analysis. (b) The same analysis restricted to the 141 detected ER167 genes. (c) Principal component analysis of the log2 counts per million of the 7155 tested genes (centred and scaled). HS donors are shown in red and healthy controls in blue. The first principal component (38.1% of the variance) separated the two groups (exact Wilcoxon P=0.0159, the smallest value attainable with four and five donors); the third component (13.5%) correlated with library size and cell number (Table S15C).

Main-text linkage: Methods 2.8; donor-level Results; Table S15.

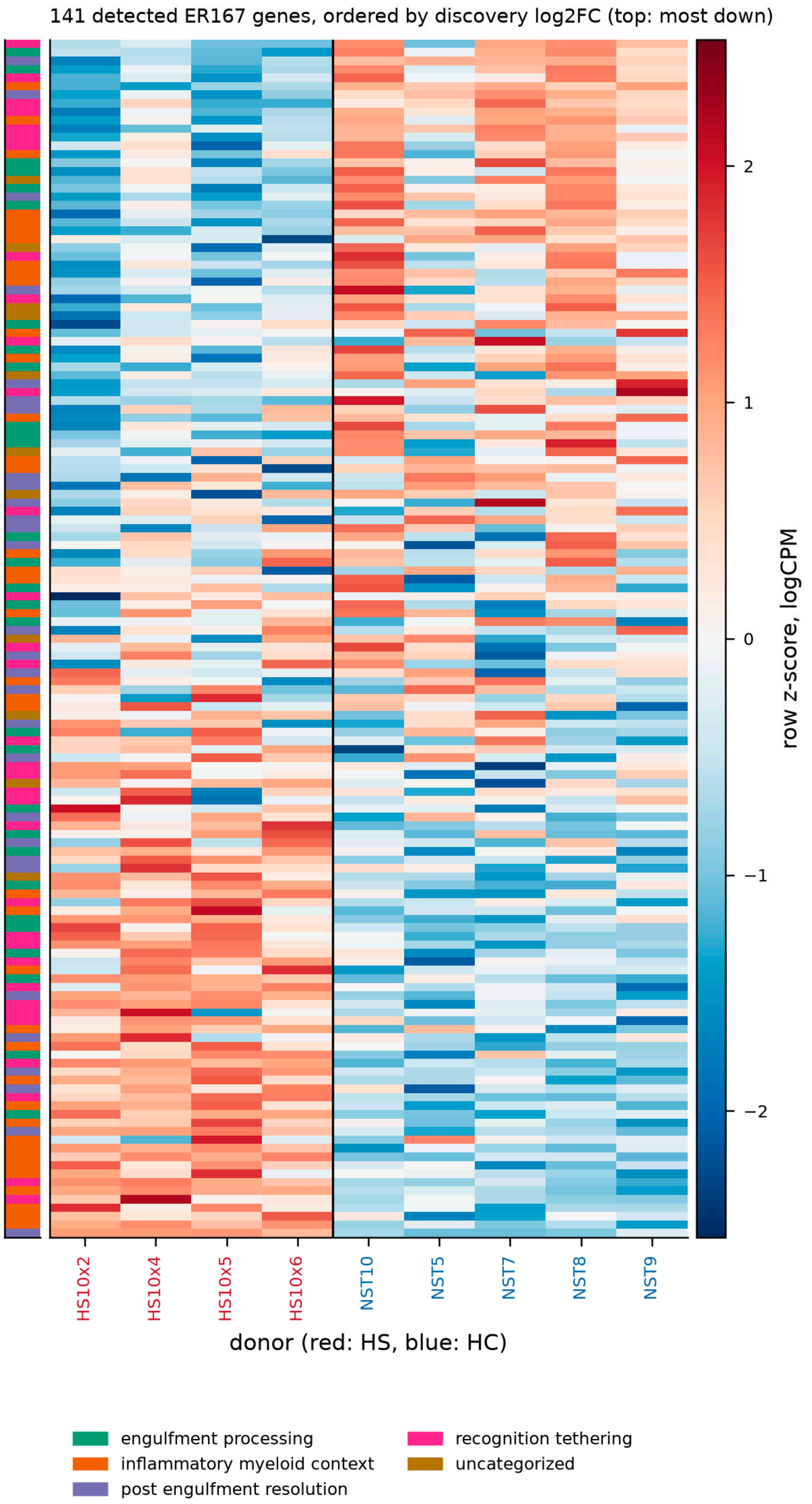

Figure S9. Donor-level expression of the detected ER167 genes in discovery. Row-standardised log2 counts per million of the 141 detected ER167 genes in the monocyte/macrophage pseudobulk profiles of the nine donors of the primary analysis (four HS, five healthy controls). Genes are ordered by the discovery log2 fold-change, with the most strongly decreased genes at the top; the bar on the left shows the manually assigned functional category of each gene.

Main-text linkage: Methods 2.8; donor-level Results; Tables S5 and S15.

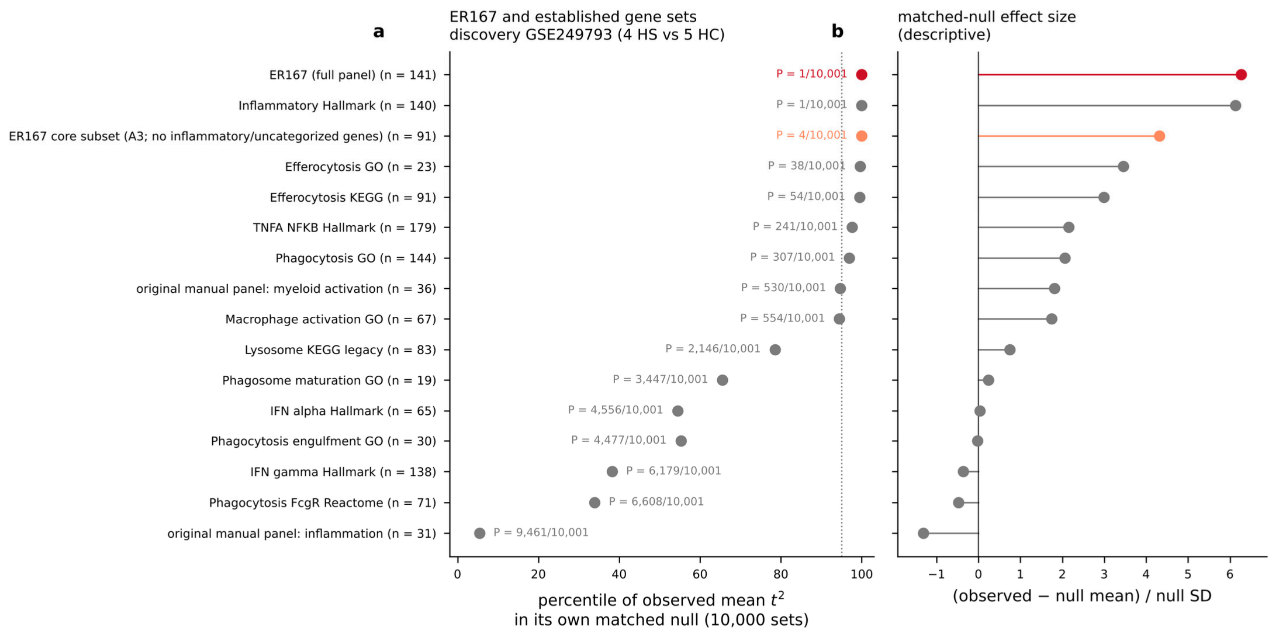

Figure S10. ER167 and established gene sets in the discovery matched-null comparison. (a) Percentile of the observed mean squared moderated t statistic of each gene set within its own null distribution of 10,000 random gene sets matched for size, expression level and detection; labels give the empirical P value as a count out of 10,001, and n is the number of detected genes. The dotted line marks the 95th percentile. (b) Distance of the observed statistic from the mean of its null distribution in units of the null standard deviation (descriptive). ER167 is shown in red and ER167 without the inflammatory/myeloid-context and uncategorized genes (set A3) in orange. Efferocytosis GO, Gene Ontology apoptotic cell clearance (GO:0043277); Efferocytosis KEGG, hsa04148; the two manual panels are the comparator panels of the original analysis (Table S6D). Gene-set definitions and results in the bulk cohorts are given in Table S12.

Main-text linkage: Methods 2.8; specificity Results; Tables S6D and S12.

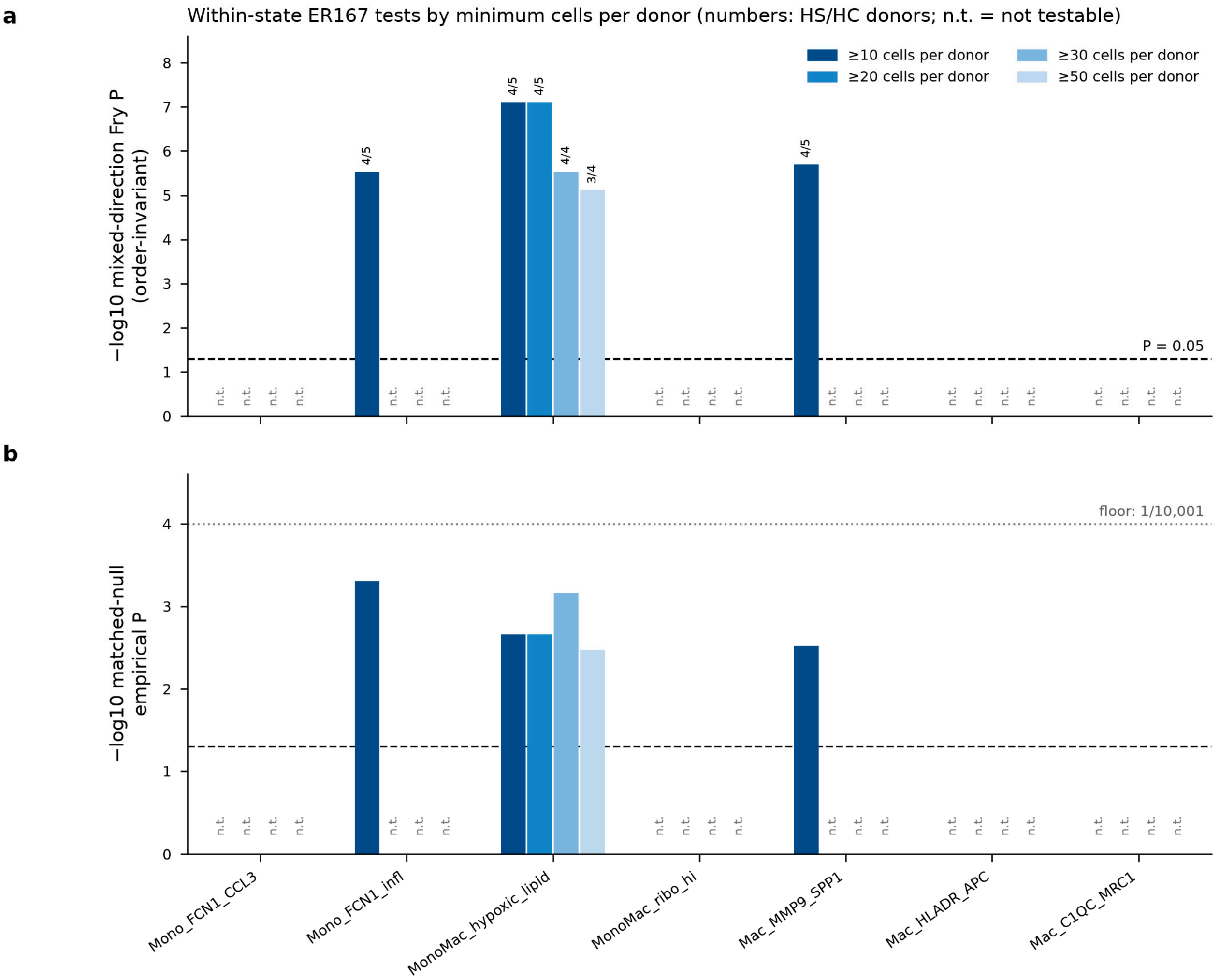

Figure S11. Within-state ER167 tests at increasing minimum numbers of cells per donor. (a) Order-invariant mixed-direction Fry P value and (b) matched-null empirical P value (10,000 matched sets; minimum attainable value 1/10,001) of ER167 within each of the seven monocyte/macrophage states, with donors required to contribute at least 10, 20, 30 or 50 cells of the state. Numbers above the bars give the numbers of HS and control donors retained; n.t., not testable (fewer than three donors in at least one group). The dashed lines mark P=0.05. Only MonoMac_hypoxic_lipid remained testable above the 10-cell threshold.

Main-text linkage: Methods 2.9; within-state Results; Tables S7B and S16.

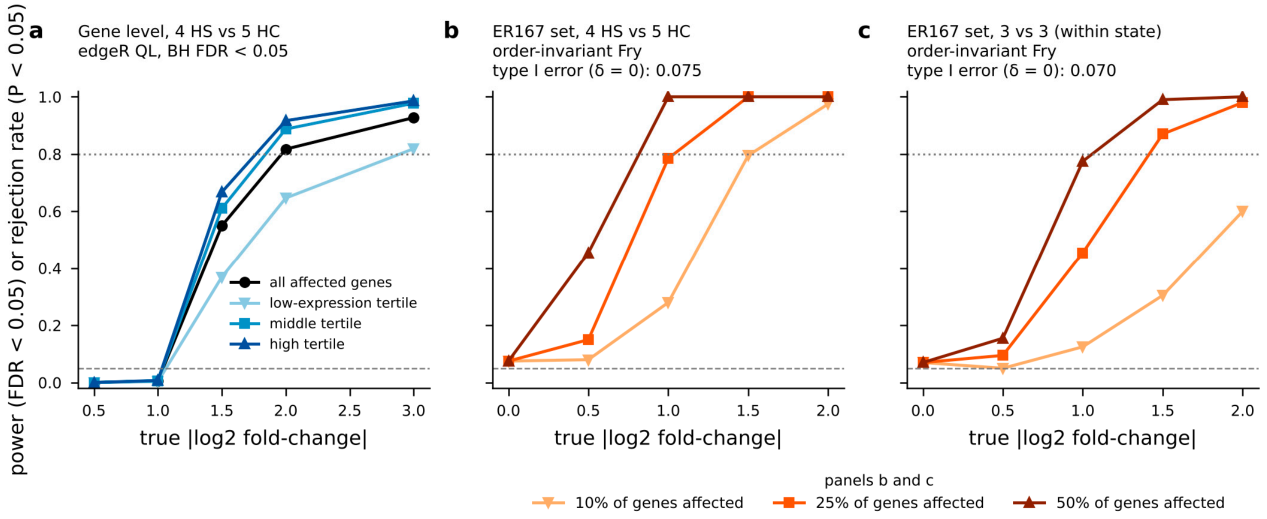

Figure S12. Simulated power of the discovery design. (a) Gene-level power of the edgeR quasi-likelihood test at a Benjamini-Hochberg FDR<0.05 with four HS and five control donors, as a function of the true absolute log2 fold-change, for all affected genes and by tertile of expression. (b) Rejection rate at P<0.05 of the order-invariant mixed-direction Fry test for a gene set of the size of ER167 in the same design, when 10%, 25% or 50% of the genes in the set are affected. (c) The same for a three-versus-three design (three HS and three control donors of the Mono_FCN1_infl state, the minimum number of donors per group required for a within-state test; the within-state tests of the article used four HS and five control donors). The value at a fold-change of zero is the type I error. Dotted lines mark 80% and dashed lines 5%. Counts were simulated from negative-binomial distributions with the observed library sizes, normalization factors, baseline abundances and tagwise dispersions (200 replicates per condition for gene sets, 100 for genes); genes were simulated independently, so the estimates are optimistic.

Main-text linkage: Methods 2.8; power Results; Table S17.

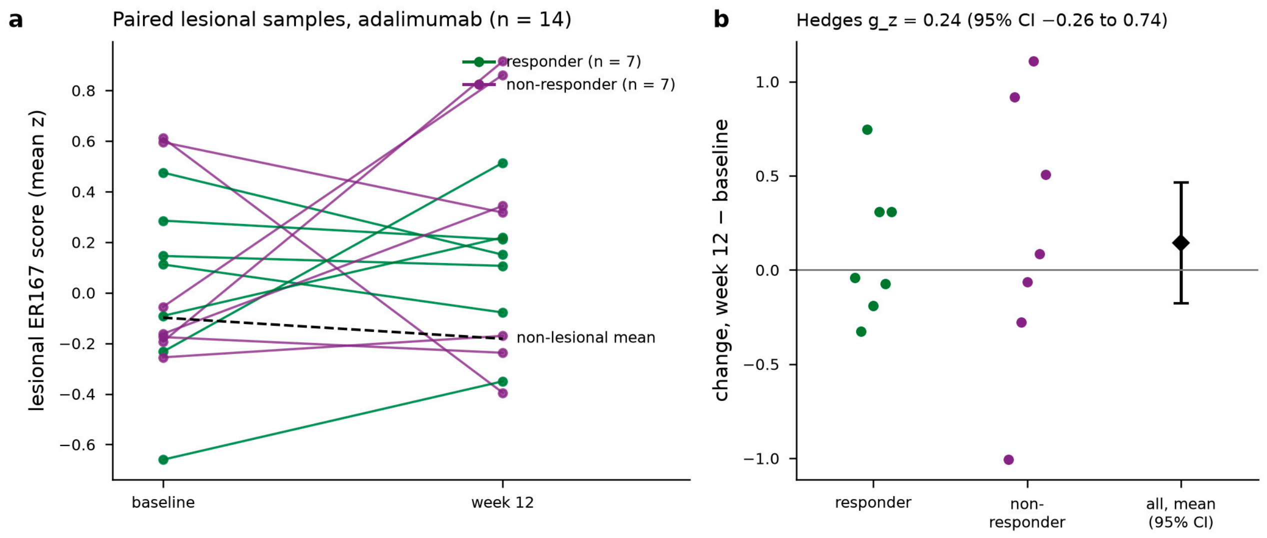

Figure S13. Lesional ER167 score before and after 12 weeks of adalimumab (GSE213761). (a) Lesional ER167 scores (mean z-standardised log2 counts per million across ER167 genes) at baseline and week 12 in the 14 patients with paired lesional samples, coloured by the responder field of the GEO record (seven responders, seven non-responders); the dashed line joins the mean non-lesional scores at the two visits. (b) Change from baseline to week 12 in each patient by responder status, and the mean change with its 95% confidence interval (0.14; −0.18 to 0.47; Hedges g=0.24, 95% confidence interval −0.26 to 0.74). The GEO record does not define the responder field (Table S18B).

Main-text linkage: Methods 2.12; treatment Results; Tables S10C and S18.

Supplementary Tables

Table numbering follows the citations in the main text.

Table S1. ER167 panel, HGNC harmonization, stable identifiers, and manually assigned functional categories

Main-text linkage: ER167 panel and category counts.

Original symbol	Current symbol	Changed	Gene name	Entrez ID	Ensembl gene ID	HGNC ID	Functional category (manual)	MyGene matches
S100P	*S100P*	No	S100 calcium binding protein P	6286	ENSG00000163993	10504	inflammatory_myeloid_context	1
TFF3	*TFF3*	No	trefoil factor 3	7033	ENSG00000160180	11757	uncategorized	1
TESC	*TESC*	No	tescalcin	54997	ENSG00000088992	26065	uncategorized	1
SAA1	*SAA1*	No	serum amyloid A1	6288	ENSG00000173432	10513	inflammatory_myeloid_context	1
SPP1	*SPP1*	No	secreted phosphoprotein 1	6696	ENSG00000118785	11255	inflammatory_myeloid_context	2
S100A9	*S100A9*	No	S100 calcium binding protein A9	6280	ENSG00000163220	10499	inflammatory_myeloid_context	1
IGFBP2	*IGFBP2*	No	insulin like growth factor binding protein 2	3485	ENSG00000115457	5471	uncategorized	1
FOSB	*FOSB*	No	FosB proto-oncogene, AP-1 transcription factor subunit	2354	ENSG00000125740	3797	inflammatory_myeloid_context	1
MARCO	*MARCO*	No	macrophage receptor with collagenous structure	8685	ENSG00000019169	6895	recognition_tethering	1
ALDH1A1	*ALDH1A1*	No	aldehyde dehydrogenase 1 family member A1	216	ENSG00000165092	402	post_engulfment_resolution	1
MMP9	*MMP9*	No	matrix metallopeptidase 9	4318	ENSG00000100985	7176	inflammatory_myeloid_context	1
LYZ	*LYZ*	No	lysozyme	4069	ENSG00000090382	6740	inflammatory_myeloid_context	1
PLAU	*PLAU*	No	plasminogen activator, urokinase	5328	ENSG00000122861	9052	inflammatory_myeloid_context	1
CCL18	*CCL18*	No	C-C motif chemokine ligand 18	6362	ENSG00000278006	10616	inflammatory_myeloid_context	1
DUSP4	*DUSP4*	No	dual specificity phosphatase 4	1846	ENSG00000120875	3070	inflammatory_myeloid_context	1
MRC1	*MRC1*	No	mannose receptor C-type 1	4360	ENSG00000260314	7228	recognition_tethering	1
BASP1	*BASP1*	No	brain abundant membrane attached signal protein 1	10409	ENSG00000176788	957	engulfment_processing	1
OLR1	*OLR1*	No	oxidized low density lipoprotein receptor 1	4973	ENSG00000173391	8133	recognition_tethering	1
HIST1H1C	*H1-2*	Yes	H1.2 linker histone, cluster member	3006	ENSG00000187837	4716	uncategorized	1
VSIG4	*VSIG4*	No	V-set and immunoglobulin domain containing 4	11326	ENSG00000155659	17032	recognition_tethering	1
VCAN	*VCAN*	No	versican	1462	ENSG00000038427	2464	inflammatory_myeloid_context	1
C1QB	*C1QB*	No	complement C1q B chain	713	ENSG00000173369	1242	recognition_tethering	1
CFD	*CFD*	No	complement factor D	1675	ENSG00000274619	2771	recognition_tethering	1
APOC1	*APOC1*	No	apolipoprotein C1	341	ENSG00000130208	607	post_engulfment_resolution	1
MCEMP1	*MCEMP1*	No	mast cell expressed membrane protein 1	199675	ENSG00000183019	27291	inflammatory_myeloid_context	1
C1QC	*C1QC*	No	complement C1q C chain	714	ENSG00000159189	1245	recognition_tethering	1
CAV1	*CAV1*	No	caveolin 1	857	ENSG00000105974	1527	inflammatory_myeloid_context	1

Original symbol	Current symbol	Changed	Gene name	Entrez ID	Ensembl gene ID	HGNC ID	Functional category (manual)	MyGene matches
CD52	*CD52*	No	CD52 molecule	1043	ENSG00000169442	1804	recognition_tethering	1
C1QA	*C1QA*	No	complement C1q A chain	712	ENSG00000173372	1241	recognition_tethering	1
ITGB8	*ITGB8*	No	integrin subunit beta 8	3696	ENSG00000105855	6163	engulfment_processing	1
CYBB	*CYBB*	No	cytochrome b-245 beta chain	1536	ENSG00000165168	2578	engulfment_processing	1
FCGR3A	*FCGR3A*	No	Fc gamma receptor IIIa	2214	ENSG00000203747	3619	recognition_tethering	1
COLEC12	*COLEC12*	No	collectin subfamily member 12	81035	ENSG00000158270	16016	recognition_tethering	1
PER1	*PER1*	No	period circadian regulator 1	5187	ENSG00000179094	8845	inflammatory_myeloid_context	1
KLF4	*KLF4*	No	KLF transcription factor 4	9314	ENSG00000136826	6348	post_engulfment_resolution	1
CD163	*CD163*	No	CD163 molecule	9332	ENSG00000177575	1631	recognition_tethering	1
IL6	*IL6*	No	interleukin 6	3569	ENSG00000136244	6018	inflammatory_myeloid_context	1
RETN	*RETN*	No	resistin	56729	ENSG00000104918	20389	post_engulfment_resolution	1
CYP27A1	*CYP27A1*	No	cytochrome P450 family 27 subfamily A member 1	1593	ENSG00000135929	2605	post_engulfment_resolution	1
MNDA	*MNDA*	No	myeloid cell nuclear differentiation antigen	4332	ENSG00000163563	7183	engulfment_processing	1
MSR1	*MSR1*	No	macrophage scavenger receptor 1	4481	ENSG00000038945	7376	recognition_tethering	1
A2M	*A2M*	No	alpha-2-macroglobulin	2	ENSG00000175899	7	recognition_tethering	1
RBP4	*RBP4*	No	retinol binding protein 4	5950	ENSG00000138207	9922	post_engulfment_resolution	2
ASPH	*ASPH*	No	aspartate beta-hydroxylase	444	ENSG00000198363	757	uncategorized	1
ODC1	*ODC1*	No	ornithine decarboxylase 1	4953	ENSG00000115758	8109	post_engulfment_resolution	2
CCL2	*CCL2*	No	C-C motif chemokine ligand 2	6347	ENSG00000108691	10618	inflammatory_myeloid_context	1
PLBD1	*PLBD1*	No	phospholipase B domain containing 1	79887	ENSG00000121316	26215	engulfment_processing	1
TYROBP	*TYROBP*	No	transmembrane immune signaling adaptor TYROBP	7305	ENSG00000011600	12449	engulfment_processing	1
EMB	*EMB*	No	embigin	133418	ENSG00000170571	30465	engulfment_processing	2
RGS16	*RGS16*	No	regulator of G protein signaling 16	6004	ENSG00000143333	9997	inflammatory_myeloid_context	1
MMP14	*MMP14*	No	matrix metallopeptidase 14	4323	ENSG00000157227	7160	inflammatory_myeloid_context	1
PPARG	*PPARG*	No	peroxisome proliferator activated receptor gamma	5468	ENSG00000132170	9236	post_engulfment_resolution	1
OSCAR	*OSCAR*	No	osteoclast associated Ig-like receptor	126014	ENSG00000170909	29960	recognition_tethering	1
BCL2A1	*BCL2A1*	No	BCL2 related protein A1	597	ENSG00000140379	991	inflammatory_myeloid_context	1
FABP5	*FABP5*	No	fatty acid binding protein 5	2171	ENSG00000164687	3560	post_engulfment_resolution	2
SIGLEC1	*SIGLEC1*	No	sialic acid binding Ig like lectin 1	6614	ENSG00000088827	11127	recognition_tethering	1
SPI1	*SPI1*	No	Spi-1 proto-oncogene	6688	ENSG00000066336	11241	inflammatory_myeloid_context	1
AQP9	*AQP9*	No	aquaporin 9	366	ENSG00000103569	643	inflammatory_myeloid_context	1

Original symbol	Current symbol	Changed	Gene name	Entrez ID	Ensembl gene ID	HGNC ID	Functional category (manual)	MyGene matches
HCK	*HCK*	No	HCK proto-oncogene, Src family tyrosine kinase	3055	ENSG00000101336	4840	engulfment_processing	1
ALOX5	*ALOX5*	No	arachidonate 5-lipoxygenase	240	ENSG00000012779	435	post_engulfment_resolution	1
PTAFR	*PTAFR*	No	platelet activating factor receptor	5724	ENSG00000169403	9582	recognition_tethering	1
SLCO2B1	*SLCO2B1*	No	solute carrier organic anion transporter family member 2B1	11309	ENSG00000137491	10962	post_engulfment_resolution	1
MS4A7	*MS4A7*	No	membrane spanning 4-domains A7	58475	ENSG00000166927	13378	inflammatory_myeloid_context	2
FABP4	*FABP4*	No	fatty acid binding protein 4	2167	ENSG00000170323	3559	post_engulfment_resolution	1
LY86	*LY86*	No	lymphocyte antigen 86	9450	ENSG00000112799	16837	recognition_tethering	1
BHLHE41	*BHLHE41*	No	basic helix-loop-helix family member e41	79365	ENSG00000123095	16617	post_engulfment_resolution	1
FCER1G	*FCER1G*	No	Fc epsilon receptor Ig	2207	ENSG00000158869	3611	recognition_tethering	1
MS4A4A	*MS4A4A*	No	membrane spanning 4-domains A4A	51338	ENSG00000110079	13371	recognition_tethering	1
FPR1	*FPR1*	No	formyl peptide receptor 1	2357	ENSG00000171051	3826	recognition_tethering	1
SOCS3	*SOCS3*	No	suppressor of cytokine signaling 3	9021	ENSG00000184557	19391	post_engulfment_resolution	1
TUBB6	*TUBB6*	No	tubulin beta 6 class V	84617	ENSG00000176014	20776	engulfment_processing	1
CSTA	*CSTA*	No	cystatin A	1475	ENSG00000121552	2481	inflammatory_myeloid_context	1
NCF2	*NCF2*	No	neutrophil cytosolic factor 2	4688	ENSG00000116701	7661	engulfment_processing	1
RGCC	*RGCC*	No	regulator of cell cycle	28984	ENSG00000102760	20369	inflammatory_myeloid_context	1
KCTD12	*KCTD12*	No	potassium channel tetramerization domain containing 12	115207	ENSG00000178695	14678	inflammatory_myeloid_context	1
PAPSS2	*PAPSS2*	No	3'-phosphoadenosine 5'-phosphosulfate synthase 2	9060	ENSG00000198682	8604	post_engulfment_resolution	1
BATF	*BATF*	No	basic leucine zipper ATF-like transcription factor	10538	ENSG00000156127	958	inflammatory_myeloid_context	1
HCST	*HCST*	No	hematopoietic cell signal transducer	10870	ENSG00000126264	16977	engulfment_processing	2
CD37	*CD37*	No	CD37 molecule	951	ENSG00000104894	1666	recognition_tethering	1
LST1	*LST1*	No	leukocyte specific transcript 1	7940	ENSG00000223465	14189	engulfment_processing	1
THBD	*THBD*	No	thrombomodulin	7056	ENSG00000178726	11784	recognition_tethering	1
FBP1	*FBP1*	No	fructose-bisphosphatase 1	2203	ENSG00000165140	3606	post_engulfment_resolution	1
HBEGF	*HBEGF*	No	heparin binding EGF like growth factor	1839	ENSG00000113070	3059	inflammatory_myeloid_context	1
EMP1	*EMP1*	No	epithelial membrane protein 1	2012	ENSG00000134531	3333	inflammatory_myeloid_context	1
CD83	*CD83*	No	CD83 molecule	9308	ENSG00000112149	1703	inflammatory_myeloid_context	1
C5AR1	*C5AR1*	No	complement C5a receptor 1	728	ENSG00000197405	1338	recognition_tethering	1
FERMT3	*FERMT3*	No	FERM domain containing kindlin 3	83706	ENSG00000149781	23151	engulfment_processing	1
SERPING1	*SERPING1*	No	serpin family G member 1	710	ENSG00000149131	1228	post_engulfment_resolution	1
FBXO32	*FBXO32*	No	F-box protein 32	114907	ENSG00000156804	16731	post_engulfment_resolution	1

Original symbol	Current symbol	Changed	Gene name	Entrez ID	Ensembl gene ID	HGNC ID	Functional category (manual)	MyGene matches
SDS	*SDS*	No	serine dehydratase	10993	ENSG00000135094	10691	post_engulfment_resolution	2
ACP5	*ACP5*	No	acid phosphatase 5, tartrate resistant	54	ENSG00000102575	124	post_engulfment_resolution	1
LAPTM5	*LAPTM5*	No	lysosomal protein transmembrane 5	7805	ENSG00000162511	29612	engulfment_processing	1
IER5L	*IER5L*	No	immediate early response 5 like	389792	ENSG00000188483	23679	inflammatory_myeloid_context	1
LAIR1	*LAIR1*	No	leukocyte associated immunoglobulin like receptor 1	3903	ENSG00000167613	6477	recognition_tethering	1
CD93	*CD93*	No	CD93 molecule	22918	ENSG00000125810	15855	recognition_tethering	1
PCOLCE2	*PCOLCE2*	No	procollagen C-endopeptidase enhancer 2	26577	ENSG00000163710	8739	post_engulfment_resolution	1
PILRA	*PILRA*	No	paired immunoglobin like type 2 receptor alpha	29992	ENSG00000085514	20396	recognition_tethering	1
SGK1	*SGK1*	No	serum/glucocorticoid regulated kinase 1	6446	ENSG00000118515	10810	post_engulfment_resolution	1
FGR	*FGR*	No	FGR proto-oncogene, Src family tyrosine kinase	2268	ENSG00000000938	3697	engulfment_processing	1
LIPA	*LIPA*	No	lipase A, lysosomal acid type	3988	ENSG00000107798	6617	post_engulfment_resolution	2
VIM	*VIM*	No	vimentin	7431	ENSG00000026025	12692	engulfment_processing	1
PTGER4	*PTGER4*	No	prostaglandin E receptor 4	5734	ENSG00000171522	9596	post_engulfment_resolution	1
IL1B	*IL1B*	No	interleukin 1 beta	3553	ENSG00000125538	5992	inflammatory_myeloid_context	1
JUNB	*JUNB*	No	JunB proto-oncogene, AP-1 transcription factor subunit	3726	ENSG00000171223	6205	inflammatory_myeloid_context	1
FCN1	*FCN1*	No	ficolin 1	2219	ENSG00000085265	3623	inflammatory_myeloid_context	1
GLIPR2	*GLIPR2*	No	GLI pathogenesis related 2	152007	ENSG00000122694	18007	post_engulfment_resolution	1
EMP3	*EMP3*	No	epithelial membrane protein 3 (MAM blood group)	2014	ENSG00000142227	3335	inflammatory_myeloid_context	1
ITGAX	*ITGAX*	No	integrin subunit alpha X	3687	ENSG00000140678	6152	engulfment_processing	1
SLC15A3	*SLC15A3*	No	solute carrier family 15 member 3	51296	ENSG00000110446	18068	post_engulfment_resolution	1
C1orf162	*C1orf162*	No	chromosome 1 open reading frame 162	128346	ENSG00000143110	28344	uncategorized	1
SLC7A7	*SLC7A7*	No	solute carrier family 7 member 7	9056	ENSG00000155465	11065	post_engulfment_resolution	1
LBH	*LBH*	No	LBH regulator of Wnt signaling pathway	81606	ENSG00000213626	29532	inflammatory_myeloid_context	2
SAMHD1	*SAMHD1*	No	SAM and HD domain containing deoxynucleoside triphosphate triphosphohydrolase 1	25939	ENSG00000101347	15925	post_engulfment_resolution	1
CD36	*CD36*	No	CD36 molecule (CD36 blood group)	948	ENSG00000135218	1663	recognition_tethering	1
SLC11A1	*SLC11A1*	No	solute carrier family 11 member 1	6556	ENSG00000018280	10907	post_engulfment_resolution	1
TLR4	*TLR4*	No	toll like receptor 4	7099	ENSG00000136869	11850	recognition_tethering	1
SIRPA	*SIRPA*	No	signal regulatory protein alpha	140885	ENSG00000198053	9662	recognition_tethering	1
TIMP1	*TIMP1*	No	TIMP metallopeptidase inhibitor 1	7076	ENSG00000102265	11820	post_engulfment_resolution	1
DAB2	*DAB2*	No	DAB adaptor protein 2	1601	ENSG00000153071	2662	engulfment_processing	1
FAM49A	*CYRIA*	Yes	CYFIP related Rac1 interactor A	81553	ENSG00000197872	25373	uncategorized	1

Original symbol	Current symbol	Changed	Gene name	Entrez ID	Ensembl gene ID	HGNC ID	Functional category (manual)	MyGene matches
PMP22	*PMP22*	No	peripheral myelin protein 22	5376	ENSG00000109099	9118	uncategorized	2
TTYH3	*TTYH3*	No	tweety family member 3	80727	ENSG00000136295	22222	uncategorized	1
CAT	*CAT*	No	catalase	847	ENSG00000121691	1516	post_engulfment_resolution	2
ABCG1	*ABCG1*	No	ATP binding cassette subfamily G member 1	9619	ENSG00000160179	73	post_engulfment_resolution	1
THEMIS2	*THEMIS2*	No	thymocyte selection associated family member 2	9473	ENSG00000130775	16839	engulfment_processing	1
SLA	*SLA*	No	Src like adaptor	6503	ENSG00000155926	10902	engulfment_processing	2
MMP19	*MMP19*	No	matrix metallopeptidase 19	4327	ENSG00000123342	7165	inflammatory_myeloid_context	1
STX11	*STX11*	No	syntaxin 11	8676	ENSG00000135604	11429	engulfment_processing	1
ENG	*ENG*	No	endoglin	2022	ENSG00000106991	3349	inflammatory_myeloid_context	1
PIK3IP1	*PIK3IP1*	No	phosphoinositide-3-kinase interacting protein 1	113791	ENSG00000100100	24942	post_engulfment_resolution	1
ARRDC4	*ARRDC4*	No	arrestin domain containing 4	91947	ENSG00000140450	28087	post_engulfment_resolution	1
JUND	*JUND*	No	JunD proto-oncogene, AP-1 transcription factor subunit	3727	ENSG00000130522	6206	inflammatory_myeloid_context	1
ACE	*ACE*	No	angiotensin I converting enzyme	1636	ENSG00000159640	2707	inflammatory_myeloid_context	1
PECAM1	*PECAM1*	No	platelet and endothelial cell adhesion molecule 1	5175	ENSG00000261371	8823	recognition_tethering	1
CCRL2	*CCRL2*	No	C-C motif chemokine receptor like 2	9034	ENSG00000121797	1612	inflammatory_myeloid_context	1
TNS3	*TNS3*	No	tensin 3	64759	ENSG00000136205	21616	engulfment_processing	1
PDIA4	*PDIA4*	No	protein disulfide isomerase family A member 4	9601	ENSG00000155660	30167	uncategorized	1
EPB41L3	*EPB41L3*	No	erythrocyte membrane protein band 4.1 like 3	23136	ENSG00000082397	3380	engulfment_processing	1
SH3BP5	*SH3BP5*	No	SH3 domain binding protein 5	9467	ENSG00000131370	10827	engulfment_processing	1
CXCL16	*CXCL16*	No	C-X-C motif chemokine ligand 16	58191	ENSG00000161921	16642	inflammatory_myeloid_context	1
ARHGAP18	*ARHGAP18*	No	Rho GTPase activating protein 18	93663	ENSG00000146376	21035	engulfment_processing	1
EMILIN2	*EMILIN2*	No	elastin microfibril interfacer 2	84034	ENSG00000132205	19881	uncategorized	1
TTC39C	*TTC39C*	No	tetratricopeptide repeat domain 39C	125488	ENSG00000168234	26595	uncategorized	1
MOB3B	*MOB3B*	No	MOB kinase activator 3B	79817	ENSG00000120162	23825	uncategorized	1
GPR65	*GPR65*	No	G protein-coupled receptor 65	8477	ENSG00000140030	4517	recognition_tethering	1
IL3RA	*IL3RA*	No	interleukin 3 receptor subunit alpha	3563	ENSG00000185291	6012	inflammatory_myeloid_context	1
SMAD7	*SMAD7*	No	SMAD family member 7	4092	ENSG00000101665	6773	post_engulfment_resolution	1
QKI	*QKI*	No	QKI, KH domain containing RNA binding	9444	ENSG00000112531	21100	post_engulfment_resolution	1
C1orf56	*C1orf56*	No	chromosome 1 open reading frame 56	54964	ENSG00000143443	26045	uncategorized	1
RCAN1	*RCAN1*	No	regulator of calcineurin 1	1827	ENSG00000159200	3040	post_engulfment_resolution	1
PDLIM1	*PDLIM1*	No	PDZ and LIM domain 1	9124	ENSG00000107438	2067	engulfment_processing	1

Original symbol	Current symbol	Changed	Gene name	Entrez ID	Ensembl gene ID	HGNC ID	Functional category (manual)	MyGene matches
DOCK4	*DOCK4*	No	dedicator of cytokinesis 4	9732	ENSG00000128512	19192	engulfment_processing	1
PDIA6	*PDIA6*	No	protein disulfide isomerase family A member 6	10130	ENSG00000143870	30168	uncategorized	1
EREG	*EREG*	No	epiregulin	2069	ENSG00000124882	3443	inflammatory_myeloid_context	1
MATK	*MATK*	No	megakaryocyte-associated tyrosine kinase	4145	ENSG00000007264	6906	engulfment_processing	1
CD300E	*CD300E*	No	CD300e molecule	342510	ENSG00000186407	28874	recognition_tethering	1
MITF	*MITF*	No	melanocyte inducing transcription factor	4286	ENSG00000187098	7105	inflammatory_myeloid_context	1
OTUD1	*OTUD1*	No	OTU deubiquitinase 1	220213	ENSG00000165312	27346	post_engulfment_resolution	1
GLDN	*GLDN*	No	gliomedin	342035	ENSG00000186417	29514	uncategorized	1
PNPLA6	*PNPLA6*	No	patatin like domain 6, lysophospholipase	10908	ENSG00000032444	16268	post_engulfment_resolution	1
CD68	*CD68*	No	CD68 molecule	968	ENSG00000129226	1693	recognition_tethering	1
LILRB3	*LILRB3*	No	leukocyte immunoglobulin like receptor B3	11025	ENSG00000204577	6607	recognition_tethering	1
SLC16A6	*SLC16A6*	No	solute carrier family 16 member 6	9120	ENSG00000108932	10927	post_engulfment_resolution	1
MCOLN1	*MCOLN1*	No	mucolipin TRP cation channel 1	57192	ENSG00000090674	13356	engulfment_processing	1
SLC31A2	*SLC31A2*	No	solute carrier family 31 member 2	1318	ENSG00000136867	11017	post_engulfment_resolution	1
FRMD4A	*FRMD4A*	No	FERM domain containing 4A	55691	ENSG00000151474	25491	engulfment_processing	1
IL1A	*IL1A*	No	interleukin 1 alpha	3552	ENSG00000115008	5991	inflammatory_myeloid_context	1

The ER167 panel was derived from the published supplementary efferocytosis-related gene list of Sun et al. (J. Cancer 2026, 17, 1419–1434); an identical list, in the same order, was published earlier as Table S1 of Zhang et al. (Transl. Cancer Res. 2025, 14, 7214–7228), and neither publication describes how the list was selected. Original gene symbols were preserved for traceability, with current HGNC symbols reported separately. Two symbols in the source list were updated to current HGNC nomenclature: HIST1H1C to H1-2 and FAM49A to CYRIA; both are one-to-one updates and did not change panel membership or size. The Methods text of the source article refers to 157 genes, whereas its supplementary list contains 167 unique entries; all 167 were retained. Functional categories were assigned manually by the authors and were not taken from the source publication or from a formal ontology; Gene Ontology and KEGG annotations of every panel gene are given in Table S13.

Table S2. Stage-0 GEO dataset inventory and cohort-level metadata summary

Main-text linkage: Dataset identification, role assignment, and the 717-record sample audit.

GSE	Analytical role	Samples	Single-cell	Raw counts	Unfiltered droplets	Groups	Platform	PubMed
GSE249793	discovery scRNA	20	Yes	yes	no/unknown	HS lesional 10; control/healthy 10	GPL24676	38113104;40690378
GSE212721	independent scRNA replication 1	9	Yes	yes	yes	HS lesional 6; control/healthy 3	GPL18573	37243544
GSE220116	scRNA replication 2 (gate)	36	Yes	yes	no/unknown	HS lesional 25; control/healthy 10; HS non-lesional 1	GPL18573	37271319
GSE274880	secondary scRNA / whole-skin ref	8	Yes	yes(series RAW.tar)	no/unknown	HS lesional 6; control/healthy 2	GPL24676	39560648
GSE249027	paired bulk 1	30	No	yes(series-level)	n/a(bulk)	HS non-lesional 15; HS lesional 15	GPL24676	38069342
GSE151243	paired bulk 2	40	No	yes(series-level)	n/a(bulk)	HS lesional 20; HS perilesional 20	GPL21290	37023004
GSE154773	external bulk	63	No	yes(series-level)	n/a(bulk)	HS lesional 43; control/healthy 20	GPL18573	32853177
GSE189266	brodalumab longitudinal	75	No	yes(series-level)	n/a(bulk)	HS lesional 25; HS perilesional 25; HS non-lesional 25	GPL24676	35191018
GSE213761	adalimumab longitudinal	59	No	yes(series-level)	n/a(bulk)	HS lesional 30; HS non-lesional 29	GPL20301	36689500
GSE155176	supporting bulk	87	No	yes(series-level)	n/a(bulk)	HS lesional 42; HS non-lesional 29; control/healthy 16	GPL11154	32841223
GSE315442	secondary tunnel	48	No	yes(series-level)	n/a(bulk)	not classified 40; control/healthy 8	GPL24676	—
GSE306437	secondary sensitivity	118	No	yes(series-level)	n/a(bulk)	HS non-lesional 118	GPL20301	42079581
GSE72702	reserve microarray	30	No	microarray(CEL)	n/a(bulk)	HS lesional 17; HS non-lesional 13	GPL13158	26707687
GSE137141	reserve	24	No	yes(series RAW.tar)	n/a(bulk)	HS lesional 8; HS non-lesional 8; control/healthy 8	GPL16699	31838778
GSE148027	reserve microarray	33	No	microarray(CEL)	n/a(bulk)	HS lesional 18; control/healthy 8; HS non-lesional 7	GPL570	32387270
GSE128637	reserve	21	No	yes(series RAW.tar)	n/a(bulk)	control/healthy 11; HS lesional 10	GPL10558	30828847
GSE245451	reserve	16	No	yes(series RAW.tar)	n/a(bulk)	control/healthy 6; HS lesional 5; HS perilesional 5	GPL17303	38183615;38534777

This table retains all 17 candidate datasets and the cohort-level fields used for analytical role assignment.

Table S3A. Go/no-go assessment for single-cell discovery, replication, and reference cohorts

Main-text linkage: Eligibility audit and role assignment.

GSE	Role	Raw counts	Donor mapping	Blood excluded	Disease structure	Macrophage gate	Ambient method	Decision
GSE249793	discovery scRNA	yes (10x MTX per lib)	yes (title-encoded: HS10x*/HS419, NST*)	yes (B-suffix libs dropped)	5 HS lesional skin + 5 healthy skin	assessed in preprocessing (≥30 cells per donor)	decontX (filtered MTX; no raw_feature_bc listed)	Included: primary discovery
GSE212721	independent scRNA replication 1	yes (RAW.tar, kallisto)	yes (HS1-6, Con1-3; 1 lib/donor)	n/a (all tissue)	6 HS + 3 healthy	assessed in preprocessing (≥30 cells per donor)	decontX after emptyDrops cell calling (FDR<0.001)	Included: replication 1
GSE220116	scRNA replication 2 (conditional)	yes (RAW.tar)	partial (HS libs fragmented by skin layer; controls shared w/ psoriasis cohort)	—	~5 HS donors (8 libs, Epi/Der split) + 10 shared controls; bulk psoriasis	assessed in preprocessing; few HS donors	decontX	Conditional: usable as secondary replication (controls shared with a psoriasis cohort); libraries merged by donor; not pooled with the discovery cohort
GSE274880	secondary scRNA / whole-skin reference	yes (RAW.tar)	yes (6 HS, 2 healthy whole-skin)	—	6 HS + 2 healthy (HC n=2)	n/a for reference use	decontX	Reference only: two healthy controls, too few for inferential replication; used as deconvolution reference

Table S3B. ER167 annotation, symbol mapping, and detection coverage across candidate cohorts

Main-text linkage: Coverage audit preceding formal cohort analyses.

GSE	Role	Platform type	ER167 annotated	Mappable symbols	Detected/expressed	Raw counts
GSE249793	discovery scRNA	scRNA	167	167	141/167	yes
GSE212721	independent scRNA replication 1	scRNA	167	167	158/167	yes
GSE220116	scRNA replication 2 (gate)	scRNA	167	167	162/167	yes
GSE274880	secondary scRNA / whole-skin ref	scRNA	167	167	Reference only; not inferentially tested	yes(series RAW.tar)
GSE249027	paired bulk 1	bulk/array	167	167	162/167	yes(series-level)
GSE151243	paired bulk 2	bulk/array	167	167	164/167	yes(series-level)
GSE154773	external bulk	bulk/array	167	167	164/167	yes(series-level)
GSE189266	brodalumab longitudinal	bulk/array	167	167	155/167	yes(series-level)
GSE213761	adalimumab longitudinal	bulk/array	167	167	161/167	yes(series-level)
GSE155176	supporting bulk	bulk/array	167	167	Not analyzed in final workflow	yes(series-level)
GSE315442	secondary tunnel	bulk/array	167	167	Not analyzed in final workflow	yes(series-level)
GSE306437	secondary sensitivity	bulk/array	167	167	Not analyzed in final workflow	yes(series-level)
GSE72702	reserve microarray	bulk/array	167	167	Not analyzed in final workflow	microarray(CEL)
GSE137141	reserve	bulk/array	167	167	Not analyzed in final workflow	yes(series RAW.tar)
GSE148027	reserve microarray	bulk/array	167	167	Not analyzed in final workflow	microarray(CEL)
GSE128637	reserve	bulk/array	167	167	Not analyzed in final workflow	yes(series RAW.tar)
GSE245451	reserve	bulk/array	167	167	Not analyzed in final workflow	yes(series RAW.tar)

Table S4. Per-donor discovery scRNA-seq quality-control metrics and mitochondrial-threshold sensitivity

Main-text linkage: Discovery preprocessing; Figures S1, S2, and S3.

Donor	Group	Raw cells	Median UMIs	Median genes	Median mtRNA (%)	Pass mt<10%	Pass mt<15%	Pass mt<20%	Median decontX
NST10	HC	4,970	7,508	1,134	2.57	4,593	4,720	4,797	0.024
NST5	HC	483	3,724	1,287	0.78	463	466	469	0.009
NST7	HC	4,022	2,284	847	3.47	3,496	3,708	3,830	0.034
NST8	HC	5,604	4,690	1,033	2.33	4,717	5,042	5,224	0.059
NST9	HC	4,669	4,095	1,173	0.61	4,501	4,566	4,601	0.003
HS10x2	HS	1,546	4,380	1,548	3.49	1,472	1,505	1,515	0.013
HS10x4	HS	6,558	2,732	1,038	4.82	5,944	6,169	6,245	0.004
HS10x5	HS	6,305	2,689	1,053	4.23	6,128	6,235	6,263	0.003
HS10x6	HS	1,869	3,524	1,130	1.14	1,824	1,833	1,835	0.010

Donor	Group	Raw cells	Median UMIs	Median genes	Median mtRNA (%)	Pass mt<10%	Pass mt<15%	Pass mt<20%	Median decontX
HS419	HS	2,575	3,242	1,289	4.56	2,455	2,525	2,550	0.028

Table S5A. Discovery macrophage pseudobulk differential-expression results for detected ER167 genes

Main-text linkage: Gene-level ER167 changes and the 62 FDR-significant genes reported in Results.

Gene	Functional category (manual)	log2FC	logCPM	P value	FDR	95% CI lower	95% CI upper
BHLHE41	post_engulfment_resolution	5.679	3.242	3.89×10^{-7}	1.46×10^{-4}	4.389	6.969
EMP1	inflammatory_myeloid_context	-3.648	7.838	5.76×10^{-7}	2.02×10^{-4}	-4.418	-2.877
TLR4	recognition_tethering	3.398	2.881	5.93×10^{-7}	2.02×10^{-4}	2.639	4.157
AQP9	inflammatory_myeloid_context	3.536	6.953	8.21×10^{-7}	2.24×10^{-4}	2.764	4.307
S100P	inflammatory_myeloid_context	5.322	3.715	8.55×10^{-7}	2.24×10^{-4}	4.036	6.608
SLC7A7	post_engulfment_resolution	2.818	5.047	1.61×10^{-6}	3.03×10^{-4}	2.164	3.471
CCRL2	inflammatory_myeloid_context	3.103	5.281	2.01×10^{-6}	3.03×10^{-4}	2.368	3.838
LAIR1	recognition_tethering	2.178	5.007	2.21×10^{-6}	3.11×10^{-4}	1.658	2.699
CD93	recognition_tethering	2.481	6.949	3.16×10^{-6}	3.90×10^{-4}	1.867	3.095
SGK1	post_engulfment_resolution	-4.578	9.508	4.94×10^{-6}	4.84×10^{-4}	-5.759	-3.397
ITGB8	engulfment_processing	2.725	7.501	9.63×10^{-6}	6.55×10^{-4}	1.975	3.475
PLBD1	engulfment_processing	-4.605	3.479	1.39×10^{-5}	7.92×10^{-4}	-6.057	-3.154
SLC11A1	post_engulfment_resolution	2.188	7.077	2.18×10^{-5}	9.95×10^{-4}	1.534	2.841
MMP14	inflammatory_myeloid_context	3.016	7.035	3.63×10^{-5}	0.0014	2.067	3.965
VCAN	inflammatory_myeloid_context	2.637	6.799	3.81×10^{-5}	0.0014	1.803	3.471
SLC15A3	post_engulfment_resolution	1.722	4.973	5.62×10^{-5}	0.0017	1.155	2.289
VSIG4	recognition_tethering	-5.573	3.241	6.60×10^{-5}	0.0018	-7.565	-3.581
PTAFR	recognition_tethering	1.857	5.106	6.90×10^{-5}	0.0019	1.232	2.481
KLF4	post_engulfment_resolution	-3.258	5.249	9.01×10^{-5}	0.0023	-4.388	-2.128
HBEGF	inflammatory_myeloid_context	2.309	6.001	1.14×10^{-4}	0.0027	1.489	3.13
THEMIS2	engulfment_processing	1.665	5.493	1.45×10^{-4}	0.003	1.058	2.272
MRC1	recognition_tethering	-3.067	5.316	1.71×10^{-4}	0.0033	-4.208	-1.927
IL1A	inflammatory_myeloid_context	3.343	7.466	2.23×10^{-4}	0.004	2.062	4.623
PDLIM1	engulfment_processing	-4.172	4.27	2.36×10^{-4}	0.0042	-5.849	-2.494
IL6	inflammatory_myeloid_context	4.08	8.336	2.42×10^{-4}	0.0042	2.502	5.657
RGCC	inflammatory_myeloid_context	-1.987	7.291	2.55×10^{-4}	0.0043	-2.76	-1.214
FCGR3A	recognition_tethering	3.595	3.241	2.57×10^{-4}	0.0044	2.166	5.024
PILRA	recognition_tethering	1.475	7.038	2.75×10^{-4}	0.0045	0.896	2.054
PTGER4	post_engulfment_resolution	-2.028	6.813	2.76×10^{-4}	0.0045	-2.825	-1.232

Gene	Functional category (manual)	log2FC	logCPM	P value	FDR	95% CI lower	95% CI upper
HCK	engulfment_processing	1.454	6.958	3.83×10^{-4}	0.0056	0.861	2.047
JUNB	inflammatory_myeloid_context	-1.918	10.233	3.98×10^{-4}	0.0057	-2.705	-1.132
EMP3	inflammatory_myeloid_context	-1.781	9.197	4.42×10^{-4}	0.0061	-2.521	-1.042
MARCO	recognition_tethering	-2.866	5.334	4.61×10^{-4}	0.0062	-4.062	-1.671
MCEMP1	inflammatory_myeloid_context	2.782	4.476	5.68×10^{-4}	0.0072	1.592	3.973
CD52	recognition_tethering	-3.754	6.776	7.01×10^{-4}	0.0081	-5.402	-2.106
IL1B	inflammatory_myeloid_context	2.084	12.589	7.06×10^{-4}	0.0081	1.167	3
CD36	recognition_tethering	-2.606	2.847	9.12×10^{-4}	0.0096	-3.831	-1.381
S100A9	inflammatory_myeloid_context	3.379	7.831	0.001	0.0103	1.82	4.937
FOSB	inflammatory_myeloid_context	-3.002	6.078	0.0011	0.0106	-4.393	-1.611
SPP1	inflammatory_myeloid_context	4.675	7.84	0.0017	0.0139	2.373	6.977
FCER1G	recognition_tethering	1.47	10.361	0.0018	0.0141	0.742	2.198
MNDA	engulfment_processing	-2.325	4.37	0.0019	0.0148	-3.489	-1.161
QKI	post_engulfment_resolution	0.882	5.417	0.002	0.0152	0.437	1.326
EPB41L3	engulfment_processing	1.113	7.115	0.002	0.0155	0.55	1.675
TTYH3	uncategorized	1.02	4.222	0.0024	0.0173	0.493	1.547
MOB3B	uncategorized	-1.326	4.621	0.0025	0.0177	-2.016	-0.636
APOC1	post_engulfment_resolution	2.419	4.443	0.003	0.0196	1.128	3.711
DOCK4	engulfment_processing	1.347	6.183	0.0032	0.0202	0.622	2.072
CYBB	engulfment_processing	1.57	3.872	0.0034	0.0211	0.717	2.423
C5AR1	recognition_tethering	-1.379	7.489	0.0043	0.0245	-2.157	-0.601
OSCAR	recognition_tethering	0.855	4.982	0.0046	0.0252	0.369	1.341
MS4A7	inflammatory_myeloid_context	1.318	7.375	0.0046	0.0254	0.567	2.07
CXCL16	inflammatory_myeloid_context	-1.504	9.605	0.0049	0.0262	-2.369	-0.638
VIM	engulfment_processing	-2.029	12.664	0.0057	0.0284	-3.224	-0.834
GPR65	recognition_tethering	1.705	3.746	0.0062	0.0299	0.689	2.72
PLAU	inflammatory_myeloid_context	1.621	5.131	0.0064	0.0306	0.649	2.593
LST1	engulfment_processing	-1.99	7.076	0.0065	0.0307	-3.186	-0.795
CYRIA	uncategorized	-1.613	8.333	0.0069	0.0323	-2.592	-0.633
STX11	engulfment_processing	-0.764	6.568	0.0083	0.0361	-1.242	-0.285
C1orf162	uncategorized	-2.281	5.208	0.0106	0.0423	-3.77	-0.791

Gene	Functional category (manual)	log2FC	logCPM	P value	FDR	95% CI lower	95% CI upper
GLIPR2	post_engulfment_resolution	-0.828	6.814	0.0119	0.0453	-1.381	-0.276
MATK	engulfment_processing	-2.322	3.868	0.0133	0.0484	-3.906	-0.738
RGS16	inflammatory_myeloid_context	-1.734	3.843	0.0141	0.0504	-2.927	-0.541
EREG	inflammatory_myeloid_context	1.17	10.255	0.0148	0.0521	0.358	1.982
SLA	engulfment_processing	2.166	2.7	0.0153	0.0533	0.649	3.683
SERPING1	post_engulfment_resolution	2.074	3.645	0.0158	0.0546	0.616	3.531
MSR1	recognition_tethering	-3.19	2.714	0.0159	0.0548	-5.496	-0.884
MS4A4A	recognition_tethering	1.613	2.528	0.0185	0.0605	0.443	2.783
PAPSS2	post_engulfment_resolution	-0.925	6.826	0.0186	0.0607	-1.596	-0.254
PECAM1	recognition_tethering	1.178	3.128	0.0187	0.0608	0.323	2.032
C1QB	recognition_tethering	1.879	5.909	0.0202	0.064	0.495	3.263
FGR	engulfment_processing	0.878	6.185	0.0209	0.0654	0.227	1.53
PER1	inflammatory_myeloid_context	-1.005	5.315	0.0219	0.0675	-1.758	-0.252
CD37	recognition_tethering	-1.569	7.274	0.0226	0.0687	-2.752	-0.385
FPR1	recognition_tethering	1.915	3.704	0.026	0.0761	0.428	3.402
H1-2	uncategorized	-1.328	4.979	0.0269	0.0778	-2.366	-0.29
LYZ	inflammatory_myeloid_context	-1.517	10.497	0.0293	0.0827	-2.726	-0.308
CAT	post_engulfment_resolution	-1.454	4.656	0.0308	0.0852	-2.624	-0.283
EMB	engulfment_processing	-0.942	2.886	0.0342	0.0918	-1.72	-0.164
MCOLN1	engulfment_processing	0.859	4.272	0.0342	0.0918	0.151	1.567
CD83	inflammatory_myeloid_context	-1.463	11.199	0.0348	0.0929	-2.675	-0.251
FCN1	inflammatory_myeloid_context	-2.411	4.915	0.0365	0.096	-4.43	-0.392
ARRDC4	post_engulfment_resolution	0.846	4.384	0.037	0.0969	0.136	1.557
LY86	recognition_tethering	-2.584	5.467	0.0374	0.0974	-4.759	-0.409
HCST	engulfment_processing	-1.175	7.804	0.047	0.1148	-2.219	-0.13
MMP19	inflammatory_myeloid_context	-0.814	7.949	0.0515	0.1223	-1.555	-0.073
EMILIN2	uncategorized	0.628	5.593	0.0549	0.1278	0.047	1.21
MMP9	inflammatory_myeloid_context	-1.27	9.645	0.0588	0.1342	-2.468	-0.073
SPI1	inflammatory_myeloid_context	-0.661	8.524	0.0602	0.1366	-1.288	-0.034
LILRB3	recognition_tethering	0.594	5.199	0.0659	0.1456	0.016	1.172
BASP1	engulfment_processing	0.741	8.967	0.0659	0.1456	0.02	1.461

Gene	Functional category (manual)	log2FC	logCPM	P value	FDR	95% CI lower	95% CI upper
PMP22	uncategorized	-0.929	6.546	0.067	0.1472	-1.836	-0.021
CFD	recognition_tethering	-0.908	5.267	0.0751	0.1595	-1.825	0.009
ARHGAP18	engulfment_processing	-1.294	5.268	0.0866	0.1766	-2.658	0.069
FBP1	post_engulfment_resolution	1	3.657	0.093	0.1857	-0.077	2.076
CCL18	inflammatory_myeloid_context	1.994	6.049	0.0931	0.1859	-0.156	4.145
LAPTM5	engulfment_processing	-0.776	8.946	0.1087	0.2073	-1.657	0.105
SIRPA	recognition_tethering	0.578	6.845	0.1209	0.2242	-0.103	1.26
SH3BP5	engulfment_processing	-0.713	7.224	0.1211	0.2244	-1.553	0.127
IL3RA	inflammatory_myeloid_context	-0.684	7.269	0.1221	0.2256	-1.493	0.125
ABCG1	post_engulfment_resolution	0.874	4.328	0.1251	0.2291	-0.168	1.916
CCL2	inflammatory_myeloid_context	2.982	9.082	0.1384	0.2471	-0.711	6.675
DAB2	engulfment_processing	0.503	5.731	0.1766	0.295	-0.185	1.192
FABP5	post_engulfment_resolution	-0.644	7.732	0.1777	0.2959	-1.528	0.24
CD300E	recognition_tethering	0.464	6.151	0.1841	0.3041	-0.183	1.112
PNPLA6	post_engulfment_resolution	0.558	4.622	0.1847	0.3046	-0.221	1.338
TTC39C	uncategorized	-0.684	3.263	0.1879	0.3085	-1.647	0.279
CD163	recognition_tethering	-1.214	4.387	0.2113	0.334	-3.02	0.592
PDIA6	uncategorized	-0.56	6.559	0.2222	0.3472	-1.416	0.295
OTUD1	post_engulfment_resolution	-0.492	5.033	0.2255	0.3509	-1.249	0.265
FERMT3	engulfment_processing	0.388	6.246	0.2288	0.3542	-0.213	0.99
ALOX5	post_engulfment_resolution	-0.815	3.078	0.2676	0.3973	-2.191	0.562
SDS	post_engulfment_resolution	-0.633	3.806	0.2795	0.41	-1.731	0.465
SAMHD1	post_engulfment_resolution	-0.545	4.168	0.295	0.4258	-1.523	0.432
C1QC	recognition_tethering	0.665	6.683	0.3245	0.457	-0.606	1.935
TIMP1	post_engulfment_resolution	-0.466	12.471	0.3496	0.4824	-1.406	0.474
SLC31A2	post_engulfment_resolution	0.357	5.412	0.3609	0.4929	-0.381	1.096
C1QA	recognition_tethering	0.662	6.32	0.4049	0.5348	-0.843	2.168
TNS3	engulfment_processing	-0.407	4.843	0.4071	0.5372	-1.338	0.523
ASPH	uncategorized	0.333	5.476	0.4308	0.5581	-0.468	1.133
A2M	recognition_tethering	-0.499	3.206	0.5035	0.6218	-1.918	0.92
BCL2A1	inflammatory_myeloid_context	0.251	9.704	0.5181	0.6355	-0.489	0.992

Gene	Functional category (manual)	log2FC	logCPM	P value	FDR	95% CI lower	95% CI upper
JUND	inflammatory_myeloid_context	-0.194	6.304	0.5699	0.6794	-0.845	0.457
ODC1	post_engulfment_resolution	-0.204	3.784	0.6124	0.7134	-0.971	0.564
ITGAX	engulfment_processing	-0.181	6.88	0.6136	0.7143	-0.864	0.503
ENG	inflammatory_myeloid_context	-0.148	6.067	0.6418	0.7366	-0.758	0.461
DUSP4	inflammatory_myeloid_context	0.146	6.668	0.7305	0.8091	-0.665	0.956
SOCS3	post_engulfment_resolution	0.133	8.435	0.7305	0.8091	-0.606	0.871
BATF	inflammatory_myeloid_context	0.133	3.427	0.7664	0.838	-0.728	0.994
RCAN1	post_engulfment_resolution	0.137	4.666	0.7765	0.8468	-0.788	1.061
CSTA	inflammatory_myeloid_context	-0.124	6.779	0.7864	0.8552	-0.999	0.752
NCF2	engulfment_processing	-0.117	5.893	0.8033	0.866	-1.023	0.788
OLR1	recognition_tethering	0.1	6.955	0.8841	0.9198	-1.212	1.411
THBD	recognition_tethering	-0.051	6.617	0.8887	0.9232	-0.751	0.649
SMAD7	post_engulfment_resolution	0.057	3.459	0.8892	0.9235	-0.724	0.838
CD68	recognition_tethering	0.051	8.118	0.8942	0.9268	-0.682	0.784
PDIA4	uncategorized	0.039	5.145	0.9136	0.9404	-0.653	0.732
TUBB6	engulfment_processing	0.012	5.211	0.9641	0.9751	-0.499	0.523
TYROBP	engulfment_processing	-0.017	10.826	0.9663	0.9766	-0.785	0.751
ACP5	post_engulfment_resolution	0.014	4.637	0.9758	0.9834	-0.872	0.899
MITF	inflammatory_myeloid_context	-0.003	4.395	0.9939	0.9957	-0.724	0.719

This table contains all 141 ER167 genes detected in discovery macrophage pseudobulk. Positive log2FC indicates higher expression in HS lesional macrophages.

Table S5B. Per-donor complementary ER167 UCell expression-score summaries

Main-text linkage: Complementary net-score analysis; the primary endpoint was the mixed-direction gene-set test.

Donor	Group	Cells	Median UCell	IQR UCell
NST10	HC	3,066	0.159	0.034
NST8	HC	3,118	0.147	0.038
NST9	HC	1,137	0.139	0.038
NST5	HC	100	0.131	0.028
NST7	HC	490	0.125	0.056
HS10x2	HS	397	0.142	0.052
HS10x5	HS	967	0.14	0.073
HS10x4	HS	220	0.132	0.068
HS10x6	HS	133	0.114	0.068
HS419	HS	14	0.055	0.027

Donor-level comparison of median ER167 UCell scores between HS and healthy controls was not significant (P=0.73). UCell was used as a complementary expression-score analysis and not as the primary inferential endpoint.

Table S6A. Primary ER167 gene-set and competitive matched-null results

Main-text linkage: Primary endpoint in discovery macrophages.

Test	Genes	Direction	P value	Notes
ER167 mixed-direction (FRY)	141	mixed	8.56×10^{-7}	PValue.Mixed from limma::fry, voom pseudobulk; order-invariant specification (default weighted test, original sample order: 5.96×10^{-9}; Table S11)
ER167 mixed-direction (ROAST msq)	141	mixed	2×10^{-4}	nrot=9999, active.prop=0.69; unweighted input (weighted input: P=1×10^{-4}, active.prop=0.71)
ER167 directional (FRY)	141	up (ns)	0.479	net directional, non-significant → mixed pattern
ER167 competitive matched-null	141	modulus	1/10,001	obs mean t^2=16.10 > all 10,000 matched sets; null mean 8.58, SD 1.20, maximum 14.57

Table S6B. Functional-category-resolved matched-null results

Main-text linkage: Category-level Results (Section 3.4) and Figure 3b.

Category	Genes	Mean t^2	Mean signed t	Proportion up	Matched-null percentile	Empirical P
inflammatory_myeloid_context	39	21.38	0.847	0.538	99.99	2/10,001
post_engulfment_resolution	29	17.807	0.44	0.483	99.79	0.0022
recognition_tethering	33	16.572	0.889	0.545	99	0.0101
engulfment_processing	29	10.372	0.438	0.517	75.31	0.247
uncategorized	11	6.578	-0.745	0.273	38.03	0.62

Empirical P values in Table S6B are nominal. Benjamini-Hochberg-adjusted values across the five categories, in row order, are 0.0010, 0.0055, 0.017, 0.31, and 0.62. Categories were assigned manually by the authors (Table S1). The single-category tests were repeated with an independent draw of random gene sets in Table S12B, which gives slightly different empirical P values.

Table S6C. Primary endpoint sensitivity to donor gates, count source, and matched-null statistic

Main-text linkage: Specificity and sensitivity Results.

Configuration	HC	HS	ER genes	Fry mixed P	Matched-null empirical P	Percentile
primary: gate30, -HS419, raw counts, mean-t^2 null	5	4	141	8.56×10^{-7}	1/10,001	100
count gate ≥20 (vs 30)	5	4	141	8.56×10^{-7}	1/10,001	100
count gate ≥50 (vs 30)	5	4	141	8.56×10^{-7}	1/10,001	100
include HS419 (gate ≥10)	5	5	134	3.20×10^{-7}	2/10,001	99.99
decontX-corrected counts (vs raw)	5	4	137	2.88×10^{-5}	3/10,001	99.98
matched-null statistic mean|t| (vs t^2)	5	4	141	8.56×10^{-7}	1/10,001	100

Fry P values in this table are from the order-invariant specification (voom log2 counts per million, observation weights removed, standardize = "none"; Methods 2.7). The values of the default weighted test in the original sample order and their distribution over random sample orderings are given in Table S11. The ≥20- and ≥50-cell gates select the same nine donors as the primary ≥30-cell gate (monocyte/macrophage cells per donor: HS10x2 397, HS10x4 220, HS10x5 967, HS10x6 133, HS419 14, NST5 100, NST7 490, NST8 3118, NST9 1137, NST10 3066); these two rows are therefore identical to the primary row and do not constitute independent sensitivity tests.

Table S6D. Specificity of ER167 relative to general-inflammation and myeloid-activation panels

Main-text linkage: Panel-specificity Results; Figure S10 and Table S12.

Panel	Genes	Observed t^2	Fry mixed P	Fry direction	Matched-null empirical P	Percentile
ER167	141	16.1	8.56×10^{-7}	Up	1/10,001	100
Inflammation	31	5.6	0.0117	Up	0.946	5.40
Myeloid_activation	36	13.15	2.57×10^{-5}	Down	0.053	94.71

Matched-null empirical P values, rather than Fry P values, quantify specificity relative to expression- and detection-matched random genes. Fry P values in this table are from the order-invariant specification (voom log2 counts per million, observation weights removed, standardize = "none"; Methods 2.7). The values of the default weighted test in the original sample order and their distribution over random sample orderings are given in Table S11. The Fry direction is the sign of the net directional statistic, which was not significant for any of the three panels (P=0.479, 0.0523 and 0.712). ER167 after removal of the genes shared with either comparator panel (115 detected genes): Fry mixed P=8.70×10^{-8}. Results for established gene sets are given in Table S12. The Inflammation and Myeloid_activation comparator panels were curated manually by the authors from canonical marker genes (38 genes each; 31 and 36 detected in the discovery pseudobulk). Inflammation: IL1B, IL6, TNF, IL18, CXCL8, CXCL9, CXCL10, CXCL11, CCL2, CCL3, CCL4, CCL5, NFKB1, NFKB2, RELA, RELB, NLRP3, CASP1, PTGS2, NOS2, IL1A, IL12B, IL23A, CXCL1, CXCL2, CXCL3, CCL20, TNFAIP3, NFKBIA, STAT1, IRF1, SOCS3, IL1RN, SAA1, SAA2, S100A8, S100A9, S100A12. Myeloid_activation: CD14, CD68, CD163, ITGAM, FCGR1A, FCGR3A, CSF1R, MERTK, LYZ, AIF1, CD86, CD80, MRC1, MSR1, MARCO, CD36, TREM2, TYROBP, FCER1G, SPI1, CEBPB, IRF8, MAFB, VSIG4, C1QA, C1QB, C1QC, APOE, APOC1, TLR2, TLR4, CD48, ITGAX, HLA-DRA, HLA-DRB1, CD74, FCN1, VCAN.

Table S7A. Donor-level monocyte/macrophage-state abundance analysis

Main-text linkage: Cell-state remodelling in Figure 4a.

State	Baseline cluster	Baseline frequency	Mean proportion HC	Mean proportion HS	Proportion ratio	t statistic	P value	FDR
Mono_FCN1_CCL3	Mono_FCN1_CCL3	0.042	0.004	0.26	0.014	-6.063	3.34×10^{-5}	2.34×10^{-4}
Mono_FCN1_infl	Mono_FCN1_infl	0.109	0.032	0.443	0.073	-4.677	3.86×10^{-4}	0.0014
Mac_C1QC_MRC1	Mac_C1QC_MRC1	0.158	0.255	0.033	7.689	3.51	0.0036	0.0084
Mac_HLADR_APC	Mac_HLADR_APC	0.131	0.096	0.007	13.845	3.231	0.0062	0.0109
Mac_MMP9_SPP1	Mac_MMP9_SPP1	0.202	0.287	0.059	4.824	2.899	0.012	0.0168
MonoMac_ribo_hi	MonoMac_ribo_hi	0.175	0.12	0.01	12.089	2.657	0.0192	0.0224
MonoMac_hypoxic_lipid	MonoMac_hypoxic_lipid	0.182	0.206	0.187	1.1	0.396	0.6984	0.6984

Table S7B. Within-state mixed-direction ER167 analyses

Main-text linkage: Within-state transcriptional component in Figure 4b.

State	HC donors	HS donors	ER genes	Direction	Directional P	Mixed-direction P
Mac_MMP9_SPP1	5	4	61	Down	0.0316	2.01×10^{-6}
Mono FCN1 infl	5	4	52	Up	0.0135	2.99×10^{-6}
MonoMac_hypoxic_lipid	5	4	86	Down	0.568	7.93×10^{-8}

Within-state significance is compatible with a transcriptional component that remains detectable after analysis is restricted to broadly defined states; it does not prove cell-autonomous efferocytic dysfunction. P values are nominal; Benjamini-Hochberg-adjusted mixed-direction P values across the three testable states are 2.99×10^{-6} (Mac_MMP9_SPP1), 2.99×10^{-6} (Mono_FCN1_infl), and 2.38×10^{-7} (MonoMac_hypoxic_lipid). Fry P values in this table are from the order-invariant specification (voom log2 counts per million, observation weights removed, standardize = "none"; Methods 2.7). The values of the default weighted test in the original sample order and their distribution over random sample orderings are given in Table S11. The direction of the net directional statistic was not stable: with the default weighted test, the Mac_MMP9_SPP1 directional P was ≥0.05 in 81% of random sample orderings, and the MonoMac_hypoxic_lipid direction differed between the weighted and the order-invariant test. The remaining states did not meet the requirement of at least 10 cells per donor in at least three donors per group. These analyses are exploratory.

Table S8A. Independent scRNA-seq replication and descriptive-cohort summary

Main-text linkage: Replication Results and Figure 5.

Cohort	Design	HS	HC	ER detected	Fry mixed P	Direction	Matched-null P	Percentile	Status
GSE249793 (discovery)	5 HS / 5 HC (HS419 gated)	4	5	141	8.56×10^{-7}	Up (NS; P=0.479)	1/10,001	100	primary
GSE212721 (replication 1)	6 HS / 3 HC	6	3	158	0.0278	Down (NS; P=0.55)	0.0296	97.05	independent replication
GSE220116 (descriptive)	5 HS / 1 HC (controls sparse)	5	1	162	0.0065	Up (descriptive)	0.1222	87.79	descriptive (1 control)

The Fry values of the discovery cohort are from the order-invariant specification (Methods 2.7). For GSE212721 and GSE220116 the values shown are from the default weighted test in the original sample order.

Table S8B. Gene-level ER167 concordance between discovery and GSE212721

Main-text linkage: Pearson correlation and sign-concordance Results; Figure S5.

Gene	Discovery log2FC	Discovery P	Discovery FDR	Replication log2FC	Replication P	Replication FDR
A2M	-0.499	0.5035	0.6218	-1.186	0.0379	0.3475
ABCG1	0.874	0.1251	0.2291	-0.264	0.5979	0.8319
ACP5	0.014	0.9758	0.9834	0.499	0.2459	0.5894
ALOX5	-0.815	0.2676	0.3973	1.226	0.0152	0.2972
APOC1	2.419	0.003	0.0196	2.169	0.0536	0.3722
AQP9	3.536	8.21×10^{-7}	2.24×10^{-4}	2.189	0.0075	0.2787
ARHGAP18	-1.294	0.0866	0.1766	-1.018	0.0044	0.2535
ARRDC4	0.846	0.037	0.0969	-0.022	0.962	0.9884
ASPH	0.333	0.4308	0.5581	0.302	0.4202	0.7171
BASP1	0.741	0.0659	0.1456	-0.899	0.1818	0.5247
BATF	0.133	0.7664	0.838	-0.062	0.8435	0.9408
BCL2A1	0.251	0.5181	0.6355	0.303	0.4801	0.7554
BHLHE41	5.679	3.89×10^{-7}	1.46×10^{-4}	-0.461	0.5784	0.8202
C1orf162	-2.281	0.0106	0.0423	0.323	0.1964	0.5389
C1QA	0.662	0.4049	0.5348	-0.623	0.2624	0.6025
C1QB	1.879	0.0202	0.064	-0.195	0.7579	0.9059
C1QC	0.665	0.3245	0.457	-0.324	0.6003	0.8327
C5AR1	-1.379	0.0043	0.0245	-0.623	0.19	0.5317
CAT	-1.454	0.0308	0.0852	0.269	0.2497	0.5929
CCL18	1.994	0.0931	0.1859	3.557	0.0155	0.2972
CCL2	2.982	0.1384	0.2471	-0.215	0.7565	0.9052
CCRL2	3.103	2.01×10^{-6}	3.03×10^{-4}	-0.217	0.5183	0.7847
CD163	-1.214	0.2113	0.334	-0.209	0.7083	0.8853
CD300E	0.464	0.1841	0.3041	0.831	0.1778	0.523
CD36	-2.606	9.12×10^{-4}	0.0096	-0.249	0.5755	0.8188
CD37	-1.569	0.0226	0.0687	-0.116	0.6388	0.8512
CD52	-3.754	7.01×10^{-4}	0.0081	0.318	0.5309	0.7913
CD68	0.051	0.8942	0.9268	0.461	0.2776	0.6116
CD83	-1.463	0.0348	0.0929	-0.556	0.2823	0.6152

Gene	Discovery log2FC	Discovery P	Discovery FDR	Replication log2FC	Replication P	Replication FDR
CD93	2.481	3.16×10^{-6}	3.90×10^{-4}	0.819	0.019	0.3027
CFD	-0.908	0.0751	0.1595	-0.046	0.8983	0.9646
CSTA	-0.124	0.7864	0.8552	0.957	0.0929	0.4237
CXCL16	-1.504	0.0049	0.0262	-0.293	0.3122	0.637
CYBB	1.57	0.0034	0.0211	1.272	0.0446	0.3575
DAB2	0.503	0.1766	0.295	-0.966	0.0549	0.3742
DOCK4	1.347	0.0032	0.0202	0.282	0.4192	0.7165
DUSP4	0.146	0.7305	0.8091	-0.678	0.1665	0.5117
EMB	-0.942	0.0342	0.0918	-1.033	0.0039	0.2492
EMILIN2	0.628	0.0549	0.1278	-0.893	0.0353	0.3475
EMP1	-3.648	5.76×10^{-7}	2.02×10^{-4}	-1.251	0.0573	0.3766
EMP3	-1.781	4.42×10^{-4}	0.0061	-0.286	0.2621	0.6023
ENG	-0.148	0.6418	0.7366	0.939	0.0014	0.2217
EPB41L3	1.113	0.002	0.0155	0.12	0.6563	0.8612
EREG	1.17	0.0148	0.0521	0.979	0.0854	0.4163
FABP5	-0.644	0.1777	0.2959	0.081	0.8668	0.9511
CYRIA	-1.613	0.0069	0.0323	-0.331	0.2617	0.6018
FBP1	1	0.093	0.1857	0.022	0.9587	0.9874
FCER1G	1.47	0.0018	0.0141	0.31	0.2688	0.6059
FCGR3A	3.595	2.57×10^{-4}	0.0044	2.39	0.0352	0.3475
FCN1	-2.411	0.0365	0.096	2.708	0.0051	0.2607
FERMT3	0.388	0.2288	0.3542	0.173	0.4357	0.7276
FGR	0.878	0.0209	0.0654	0.291	0.3004	0.6291
FOSB	-3.002	0.0011	0.0106	-0.705	0.0942	0.4249
FPR1	1.915	0.026	0.0761	2.586	0.0054	0.2642
GLIPR2	-0.828	0.0119	0.0453	0.419	0.2071	0.5535
GPR65	1.705	0.0062	0.0299	-0.528	0.1914	0.5332
HBEGF	2.309	1.14×10^{-4}	0.0027	-0.347	0.5377	0.7955
HCK	1.454	3.83×10^{-4}	0.0056	0.582	0.111	0.4472
HCST	-1.175	0.047	0.1148	0.169	0.4667	0.7469
H1-2	-1.328	0.0269	0.0778	0.161	0.698	0.8812

Gene	Discovery log2FC	Discovery P	Discovery FDR	Replication log2FC	Replication P	Replication FDR
IL1A	3.343	2.23×10^{-4}	0.004	0.778	0.2718	0.6071
IL1B	2.084	7.06×10^{-4}	0.0081	0.242	0.6039	0.8349
IL3RA	-0.684	0.1221	0.2256	-0.368	0.2883	0.6195
IL6	4.08	2.42×10^{-4}	0.0042	0.204	0.8656	0.9507
ITGAX	-0.181	0.6136	0.7143	0.335	0.3337	0.6566
ITGB8	2.725	9.63×10^{-6}	6.55×10^{-4}	0.417	0.6565	0.8612
JUNB	-1.918	3.98×10^{-4}	0.0057	-0.228	0.544	0.799
JUND	-0.194	0.5699	0.6794	-0.165	0.5702	0.8163
KLF4	-3.258	9.01×10^{-5}	0.0023	-1.194	0.0148	0.2972
LAIR1	2.178	2.21×10^{-6}	3.11×10^{-4}	0.842	0.1331	0.4723
LAPTM5	-0.776	0.1087	0.2073	-0.099	0.649	0.8574
LILRB3	0.594	0.0659	0.1456	0.86	0.0547	0.3742
LST1	-1.99	0.0065	0.0307	0.286	0.2394	0.5858
LY86	-2.584	0.0374	0.0974	0.237	0.4477	0.7355
LYZ	-1.517	0.0293	0.0827	1.165	0.0169	0.2984
MARCO	-2.866	4.61×10^{-4}	0.0062	-1.241	0.0259	0.3241
MATK	-2.322	0.0133	0.0484	-0.175	0.6551	0.8608
MCEMP1	2.782	5.68×10^{-4}	0.0072	1.07	0.1028	0.4347
MCOLN1	0.859	0.0342	0.0918	0.058	0.8107	0.9265
MITF	-0.003	0.9939	0.9957	0.089	0.8466	0.9424
MMP14	3.016	3.63×10^{-5}	0.0014	0.764	0.1573	0.5018
MMP19	-0.814	0.0515	0.1223	-0.918	0.0149	0.2972
MMP9	-1.27	0.0588	0.1342	0.223	0.5425	0.798
MNDA	-2.325	0.0019	0.0148	0.686	0.0653	0.3912
MOB3B	-1.326	0.0025	0.0177	0.72	0.0224	0.3067
MRC1	-3.067	1.71×10^{-4}	0.0033	-0.751	0.1235	0.4602
MS4A4A	1.613	0.0185	0.0605	-0.728	0.0989	0.4297
MS4A7	1.318	0.0046	0.0254	0.281	0.4628	0.7445
MSR1	-3.19	0.0159	0.0548	-0.888	0.0054	0.2642
NCF2	-0.117	0.8033	0.866	-0.254	0.3283	0.6512
ODC1	-0.204	0.6124	0.7134	-0.378	0.426	0.7214

Gene	Discovery log2FC	Discovery P	Discovery FDR	Replication log2FC	Replication P	Replication FDR
OLR1	0.1	0.8841	0.9198	2.017	0.019	0.3027
OSCAR	0.855	0.0046	0.0252	0.578	0.1701	0.5168
OTUD1	-0.492	0.2255	0.3509	-0.414	0.4161	0.7154
PAPSS2	-0.925	0.0186	0.0607	-0.744	0.1055	0.4399
PDIA4	0.039	0.9136	0.9404	0.685	0.0415	0.3489
PDIA6	-0.56	0.2222	0.3472	0.129	0.6693	0.8667
PDLIM1	-4.172	$2.36{\times}10^{-4}$	0.0042	-0.21	0.7232	0.8912
PECAM1	1.178	0.0187	0.0608	1.031	0.0103	0.2879
PER1	-1.005	0.0219	0.0675	-1.089	0.0166	0.298
PILRA	1.475	$2.75{\times}10^{-4}$	0.0045	0.73	0.1023	0.4344
PLAU	1.621	0.0064	0.0306	0.155	0.7613	0.9069
PLBD1	-4.605	$1.39{\times}10^{-5}$	$7.92{\times}10^{-4}$	0.156	0.5467	0.8012
PMP22	-0.929	0.067	0.1472	-0.291	0.5731	0.8174
PNPLA6	0.558	0.1847	0.3046	0.966	0.2305	0.5744
PTAFR	1.857	$6.90{\times}10^{-5}$	0.0019	0.187	0.5613	0.8116
PTGER4	-2.028	$2.76{\times}10^{-4}$	0.0045	-0.596	0.2139	0.5584
QKI	0.882	0.002	0.0152	0.004	0.9851	0.9954
RCAN1	0.137	0.7765	0.8468	-0.56	0.1334	0.4726
RGCC	-1.987	$2.55{\times}10^{-4}$	0.0043	-1.313	0.1135	0.4494
RGS16	-1.734	0.0141	0.0504	-0.899	0.1415	0.4843
S100A9	3.379	0.001	0.0103	4.641	0.0028	0.2492
SAMHD1	-0.545	0.295	0.4258	0.52	0.0861	0.417
SDS	-0.633	0.2795	0.41	0.851	0.0839	0.4137
SERPING1	2.074	0.0158	0.0546	0.041	0.9434	0.9809
SGK1	-4.578	$4.94{\times}10^{-6}$	$4.84{\times}10^{-4}$	-0.657	0.1612	0.5061
SH3BP5	-0.713	0.1211	0.2244	-1.12	0.0076	0.2787
SIRPA	0.578	0.1209	0.2242	0.233	0.304	0.6314
SLA	2.166	0.0153	0.0533	0.242	0.4862	0.7599
SLC11A1	2.188	$2.18{\times}10^{-5}$	$9.95{\times}10^{-4}$	1.773	0.0287	0.3312
SLC15A3	1.722	$5.62{\times}10^{-5}$	0.0017	0.12	0.7223	0.8909
SLC31A2	0.357	0.3609	0.4929	-1.15	0.0083	0.2799

Gene	Discovery log2FC	Discovery P	Discovery FDR	Replication log2FC	Replication P	Replication FDR
SLC7A7	2.818	1.61×10^{-6}	3.03×10^{-4}	0.947	0.0721	0.399
SMAD7	0.057	0.8892	0.9235	-0.695	0.1541	0.4977
SOCS3	0.133	0.7305	0.8091	0.252	0.4754	0.7523
SPI1	-0.661	0.0602	0.1366	0.393	0.0585	0.3775
SPP1	4.675	0.0017	0.0139	1.583	0.3527	0.6696
STX11	-0.764	0.0083	0.0361	-0.655	0.1873	0.5303
THBD	-0.051	0.8887	0.9232	0.222	0.597	0.8314
THEMIS2	1.665	1.45×10^{-4}	0.003	-0.143	0.708	0.8852
TIMP1	-0.466	0.3496	0.4824	0.787	0.0041	0.2492
TLR4	3.398	5.93×10^{-7}	2.02×10^{-4}	-0.129	0.6644	0.8652
TNS3	-0.407	0.4071	0.5372	0.137	0.761	0.9069
TTC39C	-0.684	0.1879	0.3085	-0.726	0.0192	0.3027
TTYH3	1.02	0.0024	0.0173	0.459	0.1982	0.5416
TUBB6	0.012	0.9641	0.9751	-0.418	0.2745	0.6094
TYROBP	-0.017	0.9663	0.9766	0.718	0.0183	0.3027
VCAN	2.637	3.81×10^{-5}	0.0014	2.552	0.0055	0.2642
VIM	-2.029	0.0057	0.0284	-0.562	0.0974	0.4281
VSIG4	-5.573	6.60×10^{-5}	0.0018	-0.567	0.3292	0.6517

This table includes all 140 ER167 genes shared with discovery.

Table S8C. ER167 gene-level effects in discovery and formal scRNA-seq replication

Main-text linkage: Discovery and gene-resolved replication Results.

Gene	Functional category (manual)	Discovery log2FC	Discovery FDR	Replication log2FC	Replication FDR
S100P	inflammatory_myeloid_context	5.322	2.24×10^{-4}	—	—
TFF3	uncategorized	—	—	—	—
TESC	uncategorized	—	—	2.208	0.2642
SAA1	inflammatory_myeloid_context	—	—	—	—
SPP1	inflammatory_myeloid_context	4.675	0.0139	1.583	0.6696
S100A9	inflammatory_myeloid_context	3.379	0.0103	4.641	0.2492
IGFBP2	uncategorized	—	—	—	—
FOSB	inflammatory_myeloid_context	-3.002	0.0106	-0.705	0.4249
MARCO	recognition_tethering	-2.866	0.0062	-1.241	0.3241
ALDH1A1	post_engulfment_resolution	—	—	-1.449	0.2915
MMP9	inflammatory_myeloid_context	-1.27	0.1342	0.223	0.798
LYZ	inflammatory_myeloid_context	-1.517	0.0827	1.165	0.2984
PLAU	inflammatory_myeloid_context	1.621	0.0306	0.155	0.9069
CCL18	inflammatory_myeloid_context	1.994	0.1859	3.557	0.2972
DUSP4	inflammatory_myeloid_context	0.146	0.8091	-0.678	0.5117
MRC1	recognition_tethering	-3.067	0.0033	-0.751	0.4602
BASP1	engulfment_processing	0.741	0.1456	-0.899	0.5247
OLR1	recognition_tethering	0.1	0.9198	2.017	0.3027
H1-2	uncategorized	-1.328	0.0778	0.161	0.8812
VSIG4	recognition_tethering	-5.573	0.0018	-0.567	0.6517
VCAN	inflammatory_myeloid_context	2.637	0.0014	2.552	0.2642
C1QB	recognition_tethering	1.879	0.064	-0.195	0.9059
CFD	recognition_tethering	-0.908	0.1595	-0.046	0.9646
APOC1	post_engulfment_resolution	2.419	0.0196	2.169	0.3722
MCEMP1	inflammatory_myeloid_context	2.782	0.0072	1.07	0.4347
C1QC	recognition_tethering	0.665	0.457	-0.324	0.8327
CAV1	inflammatory_myeloid_context	—	—	—	—
CD52	recognition_tethering	-3.754	0.0081	0.318	0.7913
C1QA	recognition_tethering	0.662	0.5348	-0.623	0.6025

Gene	Functional category (manual)	Discovery log2FC	Discovery FDR	Replication log2FC	Replication FDR
ITGB8	engulfment_processing	2.725	6.55×10^{-4}	0.417	0.8612
CYBB	engulfment_processing	1.57	0.0211	1.272	0.3575
FCGR3A	recognition_tethering	3.595	0.0044	2.39	0.3475
COLEC12	recognition_tethering	—	—	-1.497	0.4237
PER1	inflammatory_myeloid_context	-1.005	0.0675	-1.089	0.298
KLF4	post_engulfment_resolution	-3.258	0.0023	-1.194	0.2972
CD163	recognition_tethering	-1.214	0.334	-0.209	0.8853
IL6	inflammatory_myeloid_context	4.08	0.0042	0.204	0.9507
RETN	post_engulfment_resolution	—	—	-0.474	0.5837
CYP27A1	post_engulfment_resolution	—	—	-0.062	0.9802
MNDA	engulfment_processing	-2.325	0.0148	0.686	0.3912
MSR1	recognition_tethering	-3.19	0.0548	-0.888	0.2642
A2M	recognition_tethering	-0.499	0.6218	-1.186	0.3475
RBP4	post_engulfment_resolution	—	—	—	—
ASPH	uncategorized	0.333	0.5581	0.302	0.7171
ODC1	post_engulfment_resolution	-0.204	0.7134	-0.378	0.7214
CCL2	inflammatory_myeloid_context	2.982	0.2471	-0.215	0.9052
PLBD1	engulfment_processing	-4.605	7.92×10^{-4}	0.156	0.8012
TYROBP	engulfment_processing	-0.017	0.9766	0.718	0.3027
EMB	engulfment_processing	-0.942	0.0918	-1.033	0.2492
RGS16	inflammatory_myeloid_context	-1.734	0.0504	-0.899	0.4843
MMP14	inflammatory_myeloid_context	3.016	0.0014	0.764	0.5018
PPARG	post_engulfment_resolution	—	—	0.106	0.9403
OSCAR	recognition_tethering	0.855	0.0252	0.578	0.5168
BCL2A1	inflammatory_myeloid_context	0.251	0.6355	0.303	0.7554
FABP5	post_engulfment_resolution	-0.644	0.2959	0.081	0.9511
SIGLEC1	recognition_tethering	—	—	-0.735	0.4813
SPI1	inflammatory_myeloid_context	-0.661	0.1366	0.393	0.3775
AQP9	inflammatory_myeloid_context	3.536	2.24×10^{-4}	2.189	0.2787
HCK	engulfment_processing	1.454	0.0056	0.582	0.4472
ALOX5	post_engulfment_resolution	-0.815	0.3973	1.226	0.2972

Gene	Functional category (manual)	Discovery log2FC	Discovery FDR	Replication log2FC	Replication FDR
PTAFR	recognition_tethering	1.857	0.0019	0.187	0.8116
SLCO2B1	post_engulfment_resolution	—	—	-0.341	0.7037
MS4A7	inflammatory_myeloid_context	1.318	0.0254	0.281	0.7445
FABP4	post_engulfment_resolution	—	—	-4.563	0.2492
LY86	recognition_tethering	-2.584	0.0974	0.237	0.7355
BHLHE41	post_engulfment_resolution	5.679	1.46×10^{-4}	-0.461	0.8202
FCER1G	recognition_tethering	1.47	0.0141	0.31	0.6059
MS4A4A	recognition_tethering	1.613	0.0605	-0.728	0.4297
FPR1	recognition_tethering	1.915	0.0761	2.586	0.2642
SOCS3	post_engulfment_resolution	0.133	0.8091	0.252	0.7523
TUBB6	engulfment_processing	0.012	0.9751	-0.418	0.6094
CSTA	inflammatory_myeloid_context	-0.124	0.8552	0.957	0.4237
NCF2	engulfment_processing	-0.117	0.866	-0.254	0.6512
RGCC	inflammatory_myeloid_context	-1.987	0.0043	-1.313	0.4494
KCTD12	inflammatory_myeloid_context	—	—	0.032	0.961
PAPSS2	post_engulfment_resolution	-0.925	0.0607	-0.744	0.4399
BATF	inflammatory_myeloid_context	0.133	0.838	-0.062	0.9408
HCST	engulfment_processing	-1.175	0.1148	0.169	0.7469
CD37	recognition_tethering	-1.569	0.0687	-0.116	0.8512
LST1	engulfment_processing	-1.99	0.0307	0.286	0.5858
THBD	recognition_tethering	-0.051	0.9232	0.222	0.8314
FBP1	post_engulfment_resolution	1	0.1857	0.022	0.9874
HBEGF	inflammatory_myeloid_context	2.309	0.0027	-0.347	0.7955
EMP1	inflammatory_myeloid_context	-3.648	2.02×10^{-4}	-1.251	0.3766
CD83	inflammatory_myeloid_context	-1.463	0.0929	-0.556	0.6152
C5AR1	recognition_tethering	-1.379	0.0245	-0.623	0.5317
FERMT3	engulfment_processing	0.388	0.3542	0.173	0.7276
SERPING1	post_engulfment_resolution	2.074	0.0546	0.041	0.9809
FBXO32	post_engulfment_resolution	—	—	—	—
SDS	post_engulfment_resolution	-0.633	0.41	0.851	0.4137
ACP5	post_engulfment_resolution	0.014	0.9834	0.499	0.5894

Gene	Functional category (manual)	Discovery log2FC	Discovery FDR	Replication log2FC	Replication FDR
LAPTM5	engulfment_processing	-0.776	0.2073	-0.099	0.8574
IER5L	inflammatory_myeloid_context	—	—	-0.824	0.3489
LAIR1	recognition_tethering	2.178	3.11×10^{-4}	0.842	0.4723
CD93	recognition_tethering	2.481	3.90×10^{-4}	0.819	0.3027
PCOLCE2	post_engulfment_resolution	—	—	—	—
PILRA	recognition_tethering	1.475	0.0045	0.73	0.4344
SGK1	post_engulfment_resolution	-4.578	4.84×10^{-4}	-0.657	0.5061
FGR	engulfment_processing	0.878	0.0654	0.291	0.6291
LIPA	post_engulfment_resolution	—	—	0.321	0.7961
VIM	engulfment_processing	-2.029	0.0284	-0.562	0.4281
PTGER4	post_engulfment_resolution	-2.028	0.0045	-0.596	0.5584
IL1B	inflammatory_myeloid_context	2.084	0.0081	0.242	0.8349
JUNB	inflammatory_myeloid_context	-1.918	0.0057	-0.228	0.799
FCN1	inflammatory_myeloid_context	-2.411	0.096	2.708	0.2607
GLIPR2	post_engulfment_resolution	-0.828	0.0453	0.419	0.5535
EMP3	inflammatory_myeloid_context	-1.781	0.0061	-0.286	0.6023
ITGAX	engulfment_processing	-0.181	0.7143	0.335	0.6566
SLC15A3	post_engulfment_resolution	1.722	0.0017	0.12	0.8909
C1orf162	uncategorized	-2.281	0.0423	0.323	0.5389
SLC7A7	post_engulfment_resolution	2.818	3.03×10^{-4}	0.947	0.399
LBH	inflammatory_myeloid_context	—	—	-1.074	0.3516
SAMHD1	post_engulfment_resolution	-0.545	0.4258	0.52	0.417
CD36	recognition_tethering	-2.606	0.0096	-0.249	0.8188
SLC11A1	post_engulfment_resolution	2.188	9.95×10^{-4}	1.773	0.3312
TLR4	recognition_tethering	3.398	2.02×10^{-4}	-0.129	0.8652
SIRPA	recognition_tethering	0.578	0.2242	0.233	0.6314
TIMP1	post_engulfment_resolution	-0.466	0.4824	0.787	0.2492
DAB2	engulfment_processing	0.503	0.295	-0.966	0.3742
CYRIA	uncategorized	-1.613	0.0323	-0.331	0.6018
PMP22	uncategorized	-0.929	0.1472	-0.291	0.8174
TTYH3	uncategorized	1.02	0.0173	0.459	0.5416

Gene	Functional category (manual)	Discovery log2FC	Discovery FDR	Replication log2FC	Replication FDR
CAT	post_engulfment_resolution	-1.454	0.0852	0.269	0.5929
ABCG1	post_engulfment_resolution	0.874	0.2291	-0.264	0.8319
THEMIS2	engulfment_processing	1.665	0.003	-0.143	0.8852
SLA	engulfment_processing	2.166	0.0533	0.242	0.7599
MMP19	inflammatory_myeloid_context	-0.814	0.1223	-0.918	0.2972
STX11	engulfment_processing	-0.764	0.0361	-0.655	0.5303
ENG	inflammatory_myeloid_context	-0.148	0.7366	0.939	0.2217
PIK3IP1	post_engulfment_resolution	—	—	-0.646	0.4222
ARRDC4	post_engulfment_resolution	0.846	0.0969	-0.022	0.9884
JUND	inflammatory_myeloid_context	-0.194	0.6794	-0.165	0.8163
ACE	inflammatory_myeloid_context	—	—	0.267	0.8985
PECAM1	recognition_tethering	1.178	0.0608	1.031	0.2879
CCRL2	inflammatory_myeloid_context	3.103	3.03×10^{-4}	-0.217	0.7847
TNS3	engulfment_processing	-0.407	0.5372	0.137	0.9069
PDIA4	uncategorized	0.039	0.9404	0.685	0.3489
EPB41L3	engulfment_processing	1.113	0.0155	0.12	0.8612
SH3BP5	engulfment_processing	-0.713	0.2244	-1.12	0.2787
CXCL16	inflammatory_myeloid_context	-1.504	0.0262	-0.293	0.637
ARHGAP18	engulfment_processing	-1.294	0.1766	-1.018	0.2535
EMILIN2	uncategorized	0.628	0.1278	-0.893	0.3475
TTC39C	uncategorized	-0.684	0.3085	-0.726	0.3027
MOB3B	uncategorized	-1.326	0.0177	0.72	0.3067
GPR65	recognition_tethering	1.705	0.0299	-0.528	0.5332
IL3RA	inflammatory_myeloid_context	-0.684	0.2256	-0.368	0.6195
SMAD7	post_engulfment_resolution	0.057	0.9235	-0.695	0.4977
QKI	post_engulfment_resolution	0.882	0.0152	0.004	0.9954
C1orf56	uncategorized	—	—	-0.233	0.7554
RCAN1	post_engulfment_resolution	0.137	0.8468	-0.56	0.4726
PDLIM1	engulfment_processing	-4.172	0.0042	-0.21	0.8912
DOCK4	engulfment_processing	1.347	0.0202	0.282	0.7165
PDIA6	uncategorized	-0.56	0.3472	0.129	0.8667

Gene	Functional category (manual)	Discovery log2FC	Discovery FDR	Replication log2FC	Replication FDR
EREG	inflammatory_myeloid_context	1.17	0.0521	0.979	0.4163
MATK	engulfment_processing	-2.322	0.0484	-0.175	0.8608
CD300E	recognition_tethering	0.464	0.3041	0.831	0.523
MITF	inflammatory_myeloid_context	-0.003	0.9957	0.089	0.9424
OTUD1	post_engulfment_resolution	-0.492	0.3509	-0.414	0.7154
GLDN	uncategorized	—	—	—	—
PNPLA6	post_engulfment_resolution	0.558	0.3046	0.966	0.5744
CD68	recognition_tethering	0.051	0.9268	0.461	0.6116
LILRB3	recognition_tethering	0.594	0.1456	0.86	0.3742
SLC16A6	post_engulfment_resolution	—	—	0.481	0.6002
MCOLN1	engulfment_processing	0.859	0.0918	0.058	0.9265
SLC31A2	post_engulfment_resolution	0.357	0.4929	-1.15	0.2799
FRMD4A	engulfment_processing	—	—	-0.974	0.2972
IL1A	inflammatory_myeloid_context	3.343	0.004	0.778	0.6071

Table S9A. Bulk-transcriptomic ER167 validation across paired and external cohorts

Main-text linkage: Three-cohort bulk validation Results and Figure 6.

Cohort	Samples	ER genes	Fry mixed P	Direction	Directional P	Matched-null P	Percentile	Design
GSE249027	30	162	8.56×10^{-8}	Up	1.14×10^{-5}	1/10,001	100	paired lesional vs non-lesional (15 pts)
GSE151243	40	164	9.75×10^{-11}	Up	2.66×10^{-8}	1/10,001	100	paired lesional vs perilesional (20 pts)
GSE154773	32	164	2.39×10^{-9}	Up	0.00601	2/10,001	99.99	external: 22 HS lesional vs 10 healthy

Fry P values in this table are from the order-invariant specification (voom log2 counts per million, observation weights removed, standardize = "none"; Methods 2.7). The values of the default weighted test in the original sample order and their distribution over random sample orderings are given in Table S11.

Table S9B. ER167 gene-level effects across the three bulk validation cohorts

Main-text linkage: Bulk-validation Results.

Gene	GSE249027 log2FC	GSE249027 FDR	GSE151243 log2FC	GSE151243 FDR	GSE154773 log2FC	GSE154773 FDR
S100P	1.886	6.52×10^{-4}	1.329	0.0015	-0.008	0.9881
TFF3	0.493	0.0585	-0.441	0.0485	0.047	0.9424
TESC	0.632	0.0835	0.224	0.3423	0.133	0.6331
SAA1	2.5	9.38×10^{-4}	0.363	0.6107	-1.266	0.1303
SPP1	6.328	1.40×10^{-5}	5.139	1.73×10^{-7}	1.422	0.3531
S100A9	5.719	1.23×10^{-7}	4.602	6.28×10^{-7}	2.775	0.0034
IGFBP2	0.247	0.3133	-0.401	0.0835	-0.151	0.4908
FOSB	0.701	0.1313	-0.377	0.4114	-3.095	0.0052
MARCO	1.806	0.0043	1.146	0.0019	1.377	0.1481
ALDH1A1	0.822	0.0222	0.09	0.6849	-0.983	6.87×10^{-4}
MMP9	4.225	1.75×10^{-6}	2.822	2.45×10^{-8}	1.969	0.0675
LYZ	2.894	4.52×10^{-6}	2.2	9.70×10^{-8}	2.815	1.97×10^{-6}
PLAU	1.188	4.48×10^{-5}	1.464	1.91×10^{-6}	0.311	0.5514
CCL18	—	—	—	—	2.319	0.0112
DUSP4	0.154	0.4856	-0.188	0.2965	-2.063	2.07×10^{-5}
MRC1	0.863	0.0051	0.364	0.0171	1.252	0.2669
BASP1	1.578	8.78×10^{-7}	1.612	5.14×10^{-9}	1.577	1.28×10^{-5}
OLR1	4.61	1.64×10^{-4}	4.056	1.21×10^{-7}	1.433	0.3219
H1-2	1.012	0.0034	-0.123	0.518	—	—
VSIG4	1.276	0.0073	0.838	3.56×10^{-4}	2.545	1.29×10^{-4}
VCAN	1.23	0.0023	1.341	2.81×10^{-7}	1.912	1.92×10^{-6}
C1QB	2.816	1.16×10^{-6}	1.677	1.79×10^{-6}	2.491	2.76×10^{-5}
CFD	—	—	—	—	-0.968	0.1146
APOC1	2.547	0.0025	0.695	0.2632	-1.847	0.0095
MCEMP1	1.92	0.0105	2.227	2.57×10^{-4}	0.736	0.5222
C1QC	2.268	6.41×10^{-6}	1.458	7.62×10^{-7}	2.326	1.23×10^{-5}
CAV1	0.004	0.9827	-0.145	0.2499	0.128	0.6214
CD52	1.912	7.24×10^{-6}	0.96	3.12×10^{-5}	1.806	2.14×10^{-4}
C1QA	2.265	2.68×10^{-6}	1.288	1.19×10^{-5}	2.231	1.05×10^{-5}

Gene	GSE249027 log2FC	GSE249027 FDR	GSE151243 log2FC	GSE151243 FDR	GSE154773 log2FC	GSE154773 FDR
ITGB8	-0.726	2.07×10^{-4}	-0.339	0.026	-1.083	3.67×10^{-5}
CYBB	2.632	9.51×10^{-6}	1.935	3.10×10^{-7}	2.929	1.67×10^{-6}
FCGR3A	3.142	1.16×10^{-5}	3.863	1.11×10^{-8}	3.306	6.14×10^{-4}
COLEC12	0.257	0.2054	0.025	0.8776	0.788	0.0157
PER1	-1.354	7.57×10^{-6}	-1.144	6.60×10^{-8}	-0.893	0.0529
KLF4	-0.741	7.77×10^{-5}	-0.957	1.13×10^{-5}	-0.807	0.0211
CD163	1.88	2.00×10^{-4}	1.589	2.84×10^{-7}	1.843	9.66×10^{-5}
IL6	4.286	3.62×10^{-4}	4.047	3.91×10^{-5}	-2.295	0.0446
RETN	—	—	0.425	0.2232	1.396	0.0921
CYP27A1	0.653	0.1117	0.192	0.4442	-1.081	0.0061
MNDA	1.042	3.72×10^{-4}	1.606	3.32×10^{-6}	0.663	0.1702
MSR1	2.439	1.13×10^{-4}	2.067	1.10×10^{-8}	1.556	0.0041
A2M	0.947	0.0011	0.719	4.09×10^{-5}	0.942	3.68×10^{-4}
RBP4	-2.499	7.97×10^{-4}	-2.302	0.0034	-3.157	0.0023
ASPH	-0.036	0.8165	0.347	0.0123	-0.473	0.0016
ODC1	0.769	0.0252	0.052	0.8207	-0.511	0.2054
CCL2	1.84	1.33×10^{-4}	1.048	0.0031	-1.037	0.1271
PLBD1	0.892	2.13×10^{-4}	0.708	1.47×10^{-4}	1.351	3.50×10^{-7}
TYROBP	1.993	4.35×10^{-6}	1.337	2.52×10^{-7}	1.695	1.32×10^{-4}
EMB	-0.217	0.1146	0.025	0.8902	0.019	0.9294
RGS16	1.839	1.60×10^{-5}	1.544	3.39×10^{-5}	-0.813	0.09
MMP14	0.745	0.002	1.323	8.73×10^{-7}	1.797	4.71×10^{-6}
PPARG	0.348	0.3814	-0.496	0.1193	-1.9	3.00×10^{-6}
OSCAR	1.266	2.85×10^{-4}	1.16	3.86×10^{-6}	1.529	0.0016
BCL2A1	3.013	1.63×10^{-5}	3.27	1.10×10^{-7}	0.659	0.4827
FABP5	1.523	4.69×10^{-5}	1.922	1.88×10^{-7}	0.632	0.2688
SIGLEC1	1.215	0.0018	0.472	0.0135	2.266	4.01×10^{-5}
SPI1	1.007	1.81×10^{-4}	1.189	1.14×10^{-7}	1.257	0.0016
AQP9	-1.063	0.0018	-0.611	0.0086	-0.034	0.9479
HCK	1.481	2.05×10^{-5}	1.621	3.36×10^{-7}	1.329	0.0037
ALOX5	0.783	0.0014	1.299	3.95×10^{-8}	1.362	7.38×10^{-4}

Gene	GSE249027 log2FC	GSE249027 FDR	GSE151243 log2FC	GSE151243 FDR	GSE154773 log2FC	GSE154773 FDR
PTAFR	1.453	1.34×10^{-6}	0.99	2.06×10^{-6}	0.984	0.0016
SLCO2B1	1.351	1.54×10^{-5}	1.194	3.42×10^{-7}	1.01	3.55×10^{-4}
MS4A7	2.239	2.01×10^{-5}	1.822	4.99×10^{-8}	1.851	1.25×10^{-4}
FABP4	-0.334	0.6516	-0.912	0.0627	-2.188	0.0381
LY86	2.149	2.23×10^{-6}	1.715	3.59×10^{-8}	2	2.62×10^{-5}
BHLHE41	0.636	0.0391	0.668	0.0022	-0.03	0.9438
FCER1G	1.808	2.22×10^{-5}	1.541	6.57×10^{-7}	1.714	4.91×10^{-4}
MS4A4A	1.651	4.44×10^{-5}	1.052	2.09×10^{-6}	1.496	7.04×10^{-4}
FPR1	2.955	1.01×10^{-4}	3.688	2.45×10^{-8}	2.282	0.0396
SOCS3	2.731	1.52×10^{-5}	2.117	1.98×10^{-5}	-1.304	0.1003
TUBB6	0.511	0.0032	0.614	1.46×10^{-4}	-0.238	0.4603
CSTA	0.421	0.0497	0.292	0.3306	0.692	0.0548
NCF2	0.533	0.0118	0.39	0.021	0.887	0.013
RGCC	0.51	0.0054	0.322	0.0174	-0.078	0.839
KCTD12	0.508	0.0123	0.405	9.27×10^{-4}	0.357	0.0135
PAPSS2	1.998	3.62×10^{-6}	1.565	3.24×10^{-8}	1.511	3.18×10^{-5}
BATF	1.939	2.94×10^{-5}	1.33	2.57×10^{-6}	0.437	0.2425
HCST	1.692	2.83×10^{-5}	0.758	1.66×10^{-4}	1.594	2.04×10^{-5}
CD37	2.107	3.67×10^{-6}	1.214	1.19×10^{-6}	1.907	3.23×10^{-5}
LST1	—	—	—	—	1.082	0.0101
THBD	0.14	0.5289	-0.177	0.2408	-0.578	0.0551
FBP1	1.441	0.0205	0.236	0.5303	-0.815	0.2253
HBEGF	1.609	3.10×10^{-4}	0.521	0.0079	-1.767	0.0036
EMP1	-0.325	0.0037	-0.08	0.4756	-0.342	0.1192
CD83	1.522	2.39×10^{-5}	0.67	0.0022	-0.174	0.7383
C5AR1	2.269	1.37×10^{-5}	1.885	4.85×10^{-8}	0.718	0.2706
FERMT3	1.887	2.94×10^{-6}	1.378	4.89×10^{-8}	1.355	3.73×10^{-4}
SERPING1	0.589	0.0067	0.201	0.1041	0.568	0.0357
FBXO32	-0.422	0.0366	-0.564	0.0046	-0.629	0.0546
SDS	3.318	1.35×10^{-6}	2.708	2.69×10^{-8}	-0.642	0.5438
ACP5	1.644	2.14×10^{-5}	0.975	3.15×10^{-4}	0.697	0.0722

Gene	GSE249027 log2FC	GSE249027 FDR	GSE151243 log2FC	GSE151243 FDR	GSE154773 log2FC	GSE154773 FDR
LAPTM5	1.908	1.90×10^{-5}	1.678	9.13×10^{-8}	1.539	1.10×10^{-4}
IER5L	1.1	3.86×10^{-6}	0.71	4.94×10^{-6}	0.368	0.1267
LAIR1	2.512	1.66×10^{-5}	1.783	6.04×10^{-8}	1.752	3.47×10^{-4}
CD93	1.227	2.08×10^{-4}	1.35	8.22×10^{-8}	0.934	3.29×10^{-4}
PCOLCE2	-0.727	0.1037	-1.719	2.71×10^{-6}	-0.103	0.904
PILRA	1.314	8.82×10^{-4}	1.998	2.21×10^{-8}	1.506	0.0055
SGK1	1.104	5.78×10^{-6}	0.83	1.39×10^{-5}	-0.331	0.2633
FGR	1.684	1.57×10^{-5}	1.617	1.85×10^{-7}	0.919	0.0444
LIPA	1.43	2.69×10^{-5}	0.983	4.68×10^{-5}	1.202	2.04×10^{-4}
VIM	0.651	0.0018	0.202	0.1105	0.648	0.0211
PTGER4	1.031	2.39×10^{-5}	0.669	5.25×10^{-5}	-0.181	0.5079
IL1B	3.02	4.98×10^{-4}	2.908	4.17×10^{-5}	0.598	0.5819
JUNB	0.665	0.0079	0.398	0.0379	-1.468	0.0085
FCN1	2.681	2.69×10^{-6}	2.407	4.69×10^{-7}	2.388	0.0013
GLIPR2	1.122	2.92×10^{-4}	0.829	4.82×10^{-6}	1.142	6.64×10^{-4}
EMP3	0.84	5.17×10^{-4}	0.389	0.0188	0.635	0.0371
ITGAX	2.282	2.84×10^{-5}	2.234	2.46×10^{-8}	0.636	0.3717
SLC15A3	2.165	3.36×10^{-5}	1.384	1.31×10^{-7}	1.449	0.0013
C1orf162	1.103	0.0014	0.956	1.47×10^{-6}	1.404	0.0013
SLC7A7	2.478	3.77×10^{-6}	1.627	1.13×10^{-7}	2.446	1.06×10^{-5}
LBH	0.813	7.23×10^{-4}	0.581	1.58×10^{-5}	0.076	0.6715
SAMHD1	1.086	1.66×10^{-4}	0.868	6.45×10^{-7}	1.858	7.64×10^{-7}
CD36	1.375	1.66×10^{-4}	1.155	1.72×10^{-6}	-0.815	0.0084
SLC11A1	2.431	8.80×10^{-4}	2.365	1.28×10^{-7}	1.154	0.2416
TLR4	1.241	4.93×10^{-4}	0.852	3.18×10^{-4}	0.802	0.0119
SIRPA	0.474	5.89×10^{-5}	0.541	9.68×10^{-6}	0.452	0.003
TIMP1	1.407	2.51×10^{-5}	1.446	2.05×10^{-7}	0.731	0.0653
DAB2	1.139	4.09×10^{-4}	0.748	1.31×10^{-5}	0.508	0.0996
CYRIA	1.233	3.63×10^{-4}	1.07	1.41×10^{-6}	—	—
PMP22	0.167	0.4118	0.166	0.1794	0.547	0.0469
TTYH3	1.15	6.90×10^{-5}	1.601	2.45×10^{-8}	0.707	0.0035

Gene	GSE249027 log2FC	GSE249027 FDR	GSE151243 log2FC	GSE151243 FDR	GSE154773 log2FC	GSE154773 FDR
CAT	-0.861	4.10×10^{-7}	-0.575	8.53×10^{-5}	-0.568	0.002
ABCG1	0.542	0.025	0.263	0.0422	0.154	0.4839
THEMIS2	2.442	1.11×10^{-6}	1.78	5.43×10^{-8}	1.766	1.49×10^{-4}
SLA	1.125	7.35×10^{-5}	0.904	2.28×10^{-6}	1.222	4.44×10^{-4}
MMP19	1.59	1.01×10^{-5}	1.819	7.92×10^{-9}	0.715	0.0412
STX11	0.354	0.0935	0.63	1.81×10^{-4}	-0.664	0.0574
ENG	1.232	5.90×10^{-5}	1.225	2.28×10^{-6}	1.447	5.26×10^{-7}
PIK3IP1	-0.286	0.0748	-0.25	0.042	0.699	0.0244
ARRDC4	-0.491	1.90×10^{-4}	-0.244	0.061	-0.119	0.5876
JUND	0.047	0.7609	-0.332	0.0256	-0.648	0.0241
ACE	1.221	3.35×10^{-4}	0.789	2.11×10^{-5}	1.444	8.16×10^{-5}
PECAM1	1.351	4.32×10^{-5}	0.881	8.74×10^{-8}	—	—
CCRL2	2.741	1.26×10^{-6}	2.088	3.19×10^{-6}	1.325	0.0381
TNS3	0.488	0.011	0.48	0.0013	0.464	0.0899
PDIA4	0.865	4.11×10^{-6}	0.797	8.52×10^{-7}	0.944	2.13×10^{-6}
EPB41L3	0.434	0.0062	0.768	2.07×10^{-5}	1.116	7.42×10^{-5}
SH3BP5	0.798	3.84×10^{-4}	0.33	0.0029	-0.589	0.0213
CXCL16	0.54	0.004	0.624	3.00×10^{-5}	0.14	0.6488
ARHGAP18	0.52	4.86×10^{-4}	0.756	4.39×10^{-6}	0.43	0.0201
EMILIN2	0.998	0.0025	0.795	6.20×10^{-6}	1.333	1.51×10^{-4}
TTC39C	-0.432	2.44×10^{-4}	-0.316	0.001	0.017	0.9042
MOB3B	-0.14	0.0803	-0.186	0.0362	-0.031	0.8601
GPR65	2.391	2.19×10^{-6}	2.068	1.17×10^{-7}	1.405	0.0088
IL3RA	1.189	1.07×10^{-4}	0.413	0.0031	—	—
SMAD7	0.368	0.0046	0.407	0.0026	-0.124	0.5716
QKI	0.052	0.6326	0.453	3.89×10^{-4}	0.033	0.7637
C1orf56	-0.346	0.0126	-0.211	0.0632	0.204	0.1671
RCAN1	-0.129	0.4027	0.611	5.75×10^{-4}	-0.324	0.2327
PDLIM1	0.321	0.006	0.344	0.0045	0.296	0.17
DOCK4	0.29	0.1099	0.655	2.08×10^{-4}	0.346	0.1595
PDIA6	0.68	2.47×10^{-5}	0.673	2.67×10^{-6}	0.561	2.80×10^{-4}

Gene	GSE249027 log2FC	GSE249027 FDR	GSE151243 log2FC	GSE151243 FDR	GSE154773 log2FC	GSE154773 FDR
EREG	0.122	0.5437	-0.046	0.8385	-0.242	0.5694
MATK	1.496	4.71×10^{-5}	0.591	5.62×10^{-4}	0.905	0.0303
CD300E	2.239	2.17×10^{-4}	3.375	7.70×10^{-8}	1.052	0.2274
MITF	-0.103	0.6044	-0.231	0.1109	-0.148	0.6481
OTUD1	-0.626	2.47×10^{-5}	-0.536	2.09×10^{-5}	-0.042	0.8871
GLDN	-0.589	0.008	-0.371	0.0735	-0.817	0.0063
PNPLA6	0.308	0.005	0.265	0.04	0.598	2.73×10^{-5}
CD68	—	—	0.276	0.5454	—	—
LILRB3	2.414	5.43×10^{-5}	2.102	1.53×10^{-7}	1.348	0.0259
SLC16A6	0.626	7.00×10^{-4}	0.49	0.0048	0.11	0.6845
MCOLN1	-0.07	0.4958	0.079	0.4963	0.13	0.3985
SLC31A2	-0.153	0.1335	-0.454	0.0017	-0.274	0.1357
FRMD4A	-0.727	5.74×10^{-5}	-0.558	6.04×10^{-6}	0.122	0.5222
IL1A	-0.936	0.0453	-0.306	0.4821	-0.848	0.2381

Table S10A. Associations between ER167 bulk scores and estimated monocyte/macrophage fractions

Main-text linkage: Deconvolution Results and Figure 7.

Cohort	Samples	Spearman rho (all)	P (all)	Spearman rho (lesional)	P (lesional)	Mono/mac lesional	Mono/mac comparator
GSE249027	30	0.653	9.07×10^{-5}	0.339	0.2159	0.061	0
GSE151243	40	0.609	3.10×10^{-5}	0.589	0.0063	0.139	0.033

The fractions in this table are mean proportions (0.061 corresponds to 6.1%) estimated by method M1 (non-negative least squares on the 2000-gene signature, which includes 80 ER167 genes). They were not reliable and should not be interpreted quantitatively; the comparison of six composition estimates is given in Table S14.

Table S10B. Attenuation of the ER167 disease statistic after composition adjustment

Main-text linkage: Composition-adjusted bulk analysis and Figure 7.

Cohort	Unadjusted statistic	Adjusted statistic	Attenuation (%)	Unadjusted Fry P	Adjusted Fry P
GSE249027 (paired L/NL)	32.84	19.908	39.38	8.56×10^{-8}	1.22×10^{-5}
GSE151243 (paired L/periL)	43.774	13.66	68.79	9.75×10^{-11}	5.78×10^{-9}

Adjustment for the monocyte/macrophage fraction estimated by method M1. Fry P values in this table are from the order-invariant specification (voom log2 counts per million, observation weights removed, standardize = "none"; Methods 2.7). The values of the default weighted test in the original sample order and their distribution over random sample orderings are given in Table S11. Attenuation with the other composition estimates, and with all immune-cell classes as covariates, is given in Table S14.

Table S10C. Twelve-week adalimumab and brodalumab ER167 gene-set results

Main-text linkage: Treatment Results and Figure 8.

Cohort	Design	Contrast	Fry mixed P	Directional P	Direction	Interpretation
GSE213761 (adalimumab, anti-TNF)	14 pts paired lesional V1->wk12	wk12 vs baseline	0.378	0.314	Up	no detectable change at wk12
GSE189266 (brodalumab, anti-IL17RA)	lesional Baseline vs wk12 (9 v 9)	wk12 vs baseline	0.754	0.793	Up	no detectable change at wk12

Fry P values in this table are from the order-invariant specification (voom log2 counts per million, observation weights removed, standardize = "none"; Methods 2.7). The values of the default weighted test in the original sample order and their distribution over random sample orderings are given in Table S11.

Table S10D. Adalimumab longitudinal sample metadata and ER167 scores (GSE213761)
Main-text linkage: Paired lesional baseline-to-week-12 analysis.

GSM	Title	Column	Subject	Site	Visit	Responder	ER167 score
GSM6593907	5_Lesional_V12	GTG.360.B1.47	5	LS	V12	NO	-0.395
GSM6593908	5_Nonlesional_V12	GTG.360.B1.48	5	NL	V12	NO	-0.387
GSM6593909	5_Lesional_V1	GTG.360.B1.49	5	LS	V1	NO	0.612
GSM6593910	5_Nonlesional_V1	GTG.360.B1.50	5	NL	V1	NO	-0.752
GSM6593911	7_Lesional_V12	GTG.360.B2.45	7	LS	V12	YES	0.106
GSM6593912	7_Nonlesional_V12	GTG.360.B2.46	7	NL	V12	YES	-0.089
GSM6593913	7_Lesional_V1	GTG.360.B2.47	7	LS	V1	YES	0.146
GSM6593914	7_Nonlesional_V1	GTG.360.B2.48	7	NL	V1	YES	-0.27
GSM6593915	9_Lesional_V12	GTG.360.B1.51	9	LS	V12	YES	0.151
GSM6593916	9_Nonlesional_V12	GTG.360.B1.52	9	NL	V12	YES	-0.273
GSM6593917	9_Lesional_V1	GTG.360.B1.53	9	LS	V1	YES	0.475
GSM6593918	9_Nonlesional_V1	GTG.360.B1.54	9	NL	V1	YES	-0.037
GSM6593919	12_Lesional_V12	GTG.360.B2.49	12	LS	V12	NO	0.862
GSM6593920	12_Nonlesional_V12	GTG.360.B2.50	12	NL	V12	NO	-0.021
GSM6593921	12_Lesional_V1	GTG.360.B2.51	12	LS	V1	NO	-0.055
GSM6593922	12_Nonlesional_V1	GTG.360.B2.52	12	NL	V1	NO	0.033
GSM6593925	14_Lesional_V12	GTG.360.B1.55	14	LS	V12	YES	0.514
GSM6593926	14_Nonlesional_V12	GTG.360.B1.56	14	NL	V12	YES	0.461
GSM6593927	14_Lesional_V1	GTG.360.B1.57	14	LS	V1	YES	-0.233
GSM6593928	14_Nonlesional_V1	GTG.360.B1.58	14	NL	V1	YES	0.123
GSM6593929	16_Lesional_V12	X19.508.007	16	LS	V12	NO	0.317
GSM6593930	16_Lesional_V1	X19.508.008	16	LS	V1	NO	0.595
GSM6593931	18_Lesional_V12	X19.508.009	18	LS	V12	NO	-0.237
GSM6593932	18_Nonlesional_V12	X19.508.010	18	NL	V12	NO	-0.295
GSM6593933	18_Lesional_V1	X19.508.011	18	LS	V1	NO	-0.175
GSM6593934	18_Nonlesional_V1	X19.508.012	18	NL	V1	NO	-0.12
GSM6593935	21_Lesional_V12	X19.508.013	21	LS	V12	YES	-0.078
GSM6593936	21_Nonlesional_V12	X19.508.014	21	NL	V12	YES	-0.54
GSM6593937	21_Lesional_V1	X19.508.015	21	LS	V1	YES	0.112

GSM	Title	Column	Subject	Site	Visit	Responder	ER167 score
GSM6593938	21_Nonlesional_V1	X19.508.016	21	NL	V1	YES	-0.099
GSM6593939	23_Lesional_V12	X19.508.017	23	LS	V12	NO	0.915
GSM6593940	23_Nonlesional_V12	X19.508.018	23	NL	V12	NO	-0.225
GSM6593941	23_Lesional_V1	X19.508.019	23	LS	V1	NO	-0.192
GSM6593942	23_Nonlesional_V1	X19.508.020	23	NL	V1	NO	0.207
GSM6593943	24_Lesional_V12	X19.508.021	24	LS	V12	NO	-0.17
GSM6593944	24_Nonlesional_V12	X19.508.022	24	NL	V12	NO	-0.366
GSM6593945	24_Lesional_V1	X19.508.023	24	LS	V1	NO	-0.255
GSM6593946	24_Nonlesional_V1	X19.508.024	24	NL	V1	NO	-0.925
GSM6593947	25_Lesional_V12	X19.508.025	25	LS	V12	YES	0.087
GSM6593948	25_Nonlesional_V12	X19.508.026	25	NL	V12	YES	0.249
GSM6593949	25_Nonlesional_V1	X19.508.028	25	NL	V1	YES	0.078
GSM6593950	26_Lesional_V12	X19.508.029	26	LS	V12	YES	-0.35
GSM6593951	26_Nonlesional_V12	X19.508.030	26	NL	V12	YES	-0.056
GSM6593952	26_Lesional_V1	X19.508.031	26	LS	V1	YES	-0.66
GSM6593953	26_Nonlesional_V1	X19.508.032	26	NL	V1	YES	0.345
GSM6593923	13_Lesional_V1	X20.559.001	13	LS	V1	NO	0.289
GSM6593924	13_Nonlesional_V1	X20.559.002	13	NL	V1	NO	0.127
GSM6593954	27_Lesional_V12	X20.559.005	27	LS	V12	YES	0.211
GSM6593955	27_Nonlesional_V12	X20.559.006	27	NL	V12	YES	-0.161
GSM6593956	27_Lesional_V1	X20.559.003	27	LS	V1	YES	0.285
GSM6593957	27_Nonlesional_V1	X20.559.004	27	NL	V1	YES	0.784
GSM6593958	28_Lesional_V12	X20.559.009	28	LS	V12	NO	0.344
GSM6593959	28_Nonlesional_V12	X20.559.010	28	NL	V12	NO	-0.573
GSM6593960	28_Lesional_V1	X20.559.007	28	LS	V1	NO	-0.162
GSM6593961	28_Nonlesional_V1	X20.559.008	28	NL	V1	NO	-0.362
GSM6593962	31_Lesional_V12	X20.559.013	31	LS	V12	YES	0.219
GSM6593963	31_Nonlesional_V12	X20.559.014	31	NL	V12	YES	0.163
GSM6593964	31_Lesional_V1	X20.559.011	31	LS	V1	YES	-0.091
GSM6593965	31_Nonlesional_V1	X20.559.012	31	NL	V1	YES	-0.208

Table S10E. Brodalumab sample metadata and ER167 scores by time point (GSE189266)
Main-text linkage: Cross-sectional time-point analysis because donor linkage was unavailable.

GSM	Column	Time point	Compartment	ER167 score	Grouped time point
GSM5696793	samX11	Baseline	Perilesional	0.09	Baseline
GSM5696795	samX13	Week2	Lesional	-0.188	Early(wk2-4)
GSM5696805	samX6	Week4	Nonlesional	-0.372	Early(wk2-4)
GSM5696800	samX20	Baseline	Perilesional	0.324	Baseline
GSM5696801	samX21	Baseline	Nonlesional	-0.195	Baseline
GSM5696806	samX7	Week2	Lesional	0.44	Early(wk2-4)
GSM5696807	samX8	Week2	Perilesional	0.793	Early(wk2-4)
GSM5696791	samX1	Baseline	Lesional	-0.149	Baseline
GSM5696792	samX10	Baseline	Lesional	0.486	Baseline
GSM5696796	samX14	Week2	Perilesional	-0.254	Early(wk2-4)
GSM5696804	samX5	Week4	Perilesional	0.022	Early(wk2-4)
GSM5696799	samX2	Baseline	Perilesional	-0.077	Baseline
GSM5696803	samX4	Week4	Lesional	-0.174	Early(wk2-4)
GSM5696808	samX9	Week2	Nonlesional	-0.396	Early(wk2-4)
GSM5696794	samX12	Baseline	Nonlesional	-0.346	Baseline
GSM5696798	samX19	Baseline	Lesional	0.484	Baseline
GSM5696802	samX3	Baseline	Nonlesional	-0.199	Baseline
GSM5696797	samX15	Week2	Nonlesional	-0.26	Early(wk2-4)
GSM5696837	samAx53	Baseline	Perilesional	0.553	Baseline
GSM5696836	samAx52	Baseline	Lesional	0.25	Baseline
GSM5696829	samAx45	Baseline	Nonlesional	-0.034	Baseline
GSM5696817	samAx33	Week12	Nonlesional	0.111	Late(wk8-12)
GSM5696821	samAx37	Week4	Lesional	-0.295	Early(wk2-4)
GSM5696839	samAx55	Week4	Lesional	0.172	Early(wk2-4)
GSM5696834	samAx50	Week12	Perilesional	0.139	Late(wk8-12)
GSM5696863	samAx79	Week12	Lesional	0.426	Late(wk8-12)
GSM5696856	samAx72	Week12	Nonlesional	-0.068	Late(wk8-12)
GSM5696811	samAx24	Week12	Nonlesional	-0.271	Late(wk8-12)

GSM	Column	Time point	Compartment	ER167 score	Grouped time point
GSM5696819	samAx35	Week12	Perilesional	-0.41	Late(wk8-12)
GSM5696849	samAx65	Week12	Perilesional	-0.42	Late(wk8-12)
GSM5696862	samAx78	Week4	Nonlesional	0.026	Early(wk2-4)
GSM5696825	samAx41	Week12	Perilesional	0.403	Late(wk8-12)
GSM5696835	samAx51	Week12	Nonlesional	-0.447	Late(wk8-12)
GSM5696816	samAx32	Week12	Perilesional	0.764	Late(wk8-12)
GSM5696812	samAx28	Baseline	Lesional	0.257	Baseline
GSM5696857	samAx73	Baseline	Lesional	0.006	Baseline
GSM5696810	samAx23	Week12	Perilesional	-0.219	Late(wk8-12)
GSM5696842	samAx58	Week12	Lesional	0.025	Late(wk8-12)
GSM5696809	samAx22	Week12	Lesional	-0.102	Late(wk8-12)
GSM5696815	samAx31	Week12	Lesional	0.848	Late(wk8-12)
GSM5696859	samAx75	Baseline	Nonlesional	-0.272	Baseline
GSM5696838	samAx54	Baseline	Nonlesional	0.028	Baseline
GSM5696852	samAx68	Baseline	Perilesional	0.997	Baseline
GSM5696827	samAx43	Baseline	Lesional	0.606	Baseline
GSM5696823	samAx39	Week4	Nonlesional	-0.463	Early(wk2-4)
GSM5696831	samAx47	Week8	Perilesional	-0.15	Late(wk8-12)
GSM5696824	samAx40	Week12	Lesional	0.472	Late(wk8-12)
GSM5696845	samAx61	Baseline	Lesional	-0.466	Baseline
GSM5696858	samAx74	Baseline	Perilesional	0.108	Baseline
GSM5696861	samAx77	Week4	Perilesional	0.217	Early(wk2-4)
GSM5696850	samAx66	Week12	Nonlesional	-0.675	Late(wk8-12)
GSM5696832	samAx48	Week8	Nonlesional	-0.403	Late(wk8-12)
GSM5696864	samAx80	Week12	Perilesional	-0.242	Late(wk8-12)
GSM5696840	samAx56	Week4	Perilesional	0.798	Early(wk2-4)
GSM5696865	samAx81	Week12	Nonlesional	-0.432	Late(wk8-12)
GSM5696847	samAx63	Baseline	Nonlesional	-0.361	Baseline
GSM5696853	samAx69	Baseline	Nonlesional	-0.306	Baseline
GSM5696822	samAx38	Week4	Perilesional	-0.516	Early(wk2-4)

GSM	Column	Time point	Compartment	ER167 score	Grouped time point
GSM5696855	samAx71	Week12	Perilesional	-0.856	Late(wk8-12)
GSM5696830	samAx46	Week8	Lesional	0.864	Late(wk8-12)
GSM5696854	samAx70	Week12	Lesional	-0.157	Late(wk8-12)
GSM5696846	samAx62	Baseline	Perilesional	-0.371	Baseline
GSM5696833	samAx49	Week12	Lesional	0.655	Late(wk8-12)
GSM5696860	samAx76	Week4	Lesional	0.767	Early(wk2-4)
GSM5696848	samAx64	Week12	Lesional	-0.523	Late(wk8-12)
GSM5696844	samAx60	Week12	Nonlesional	-0.317	Late(wk8-12)
GSM5696820	samAx36	Week12	Nonlesional	-0.379	Late(wk8-12)
GSM5696814	samAx30	Baseline	Nonlesional	-0.375	Baseline
GSM5696828	samAx44	Baseline	Perilesional	0.18	Baseline
GSM5696841	samAx57	Week4	Nonlesional	0.015	Early(wk2-4)
GSM5696818	samAx34	Week12	Lesional	-0.249	Late(wk8-12)
GSM5696851	samAx67	Baseline	Lesional	-0.237	Baseline
GSM5696826	samAx42	Week12	Nonlesional	-0.643	Late(wk8-12)
GSM5696813	samAx29	Baseline	Perilesional	0.65	Baseline
GSM5696843	samAx59	Week12	Perilesional	0.292	Late(wk8-12)

Table S10F. Disease-treatment ER167 vector correlations across both disease references and both biologics

Main-text linkage: Directional-vector Results; Figure S6; Table S18D.

Disease reference	Treatment	Genes	Spearman rho	Permutation P (two-sided)
GSE154773	adalimumab	156	0.517	0.110
GSE154773	brodalumab	152	0.219	0.702
GSE249027	adalimumab	157	0.394	0.324
GSE249027	brodalumab	155	0.426	0.573

A negative correlation would support directional reversal. All observed correlations were positive, but none differed from the distribution obtained when the sample labels were permuted (10,000 permutations; Methods 2.12); the correlations are therefore descriptive. They give no indication of directional reversal in the available 12-week transcriptomic data and do not establish persistence of efferocytic dysfunction. Conventional gene-level P values are not given, because the genes of the panel are not independent. Permutation distributions and the comparison with random gene sets are given in Table S18D and Figure S6.

Table S11. Order dependence of the weighted Fry test and order-invariant values for every reported Fry result

Main-text linkage: Methods 2.7; all Fry P values in the Results; Figure S7.

Table	Analysis	Samples	ER167 genes tested	Order-invariant mixed P (reported)	Order-invariant directional P (direction)	Standardised order-invariant mixed P	Weighted test, original order: mixed P	Weighted test, original order: directional P (direction)	Random orderings (n)	Weighted test over orderings: median mixed P	Weighted test over orderings: 2.5th–97.5th percentiles
Table S6A	Discovery GSE249793 mono/mac pseudobulk, ER167, HS vs HC	9	141	8.56×10^{-7}	0.479 (up)	1.37×10^{-8}	5.96×10^{-9}	0.0926 (up)	1,000	1.60×10^{-8}	9.06×10^{-9} to 3.23×10^{-8}
Table S6C	Sensitivity: primary: gate ≥30, HS419 excluded, raw counts (4 HS vs 5 HC)	9	141	8.56×10^{-7}	0.479 (up)	1.37×10^{-8}	5.96×10^{-9}	0.0926 (up)	200	1.58×10^{-8}	9.03×10^{-9} to 2.79×10^{-8}
Table S6C	Sensitivity: cell-count gate ≥20 (4 HS vs 5 HC)	9	141	8.56×10^{-7}	0.479 (up)	1.37×10^{-8}	5.96×10^{-9}	0.0926 (up)	200	1.58×10^{-8}	9.03×10^{-9} to 2.79×10^{-8}
Table S6C	Sensitivity: cell-count gate ≥50 (4 HS vs 5 HC)	9	141	8.56×10^{-7}	0.479 (up)	1.37×10^{-8}	5.96×10^{-9}	0.0926 (up)	200	1.58×10^{-8}	9.03×10^{-9} to 2.79×10^{-8}
Table S6C	Sensitivity: HS419 included (gate ≥10) (5 HS vs 5 HC)	10	134	3.20×10^{-7}	0.69 (up)	1.32×10^{-9}	4.17×10^{-9}	0.327 (up)	200	6.82×10^{-9}	4.50×10^{-9} to 1.16×10^{-8}
Table S6C	Sensitivity: decontX-corrected counts (4 HS vs 5 HC)	9	137	2.88×10^{-5}	0.487 (up)	7.19×10^{-8}	1.13×10^{-8}	0.247 (up)	200	3.68×10^{-8}	1.81×10^{-8} to 7.88×10^{-8}
Table S6D	Discovery primary pseudobulk, panel: ER167	9	141	8.56×10^{-7}	0.479 (up)	1.37×10^{-8}	5.96×10^{-9}	0.0926 (up)	200	1.58×10^{-8}	9.03×10^{-9} to 2.79×10^{-8}
Table S6D	Discovery primary pseudobulk, panel: manual general-inflammation panel	9	31	0.0117	0.0523 (up)	2.88×10^{-4}	0.00157	0.126 (up)	200	0.00105	6.91×10^{-4} to 0.00359
Table S6D	Discovery primary pseudobulk, panel: manual myeloid-activation panel	9	36	2.57×10^{-5}	0.712 (down)	6.18×10^{-7}	2.68×10^{-8}	0.687 (up)	200	1.39×10^{-7}	4.40×10^{-8} to 5.15×10^{-7}
Table S6D	Discovery primary pseudobulk, panel: ER167 without the genes of both manual panels	9	115	8.70×10^{-8}	0.567 (up)	6.59×10^{-9}	4.58×10^{-9}	0.134 (up)	200	1.09×10^{-8}	6.62×10^{-9} to 1.91×10^{-8}
Table S7B	Within-state Mac_MMP9_SPP1 (4 HS vs 5 HC)	9	61	2.01×10^{-6}	0.0316 (down)	1.04×10^{-7}	5.61×10^{-8}	0.0388 (down)	1,000	6.97×10^{-8}	3.85×10^{-8} to 1.25×10^{-7}

Table	Analysis	Samples	ER167 genes tested	Order-invariant mixed P (reported)	Order-invariant directional P (direction)	Standardised order-invariant mixed P	Weighted test, original order: mixed P	Weighted test, original order: directional P (direction)	Random orderings (n)	Weighted test over orderings: median mixed P	Weighted test over orderings: 2.5th–97.5th percentiles
Table S7B	Within-state MonoMac_hypoxic_lipid (4 HS vs 5 HC)	9	86	7.93×10^{-8}	0.568 (down)	1.71×10^{-9}	1.90×10^{-9}	0.64 (up)	1,000	1.14×10^{-8}	2.24×10^{-9} to 3.43×10^{-8}
Table S7B	Within-state Mono_FCN1_infl (4 HS vs 5 HC)	9	52	2.99×10^{-6}	0.0135 (up)	3.04×10^{-8}	1.16×10^{-5}	0.0115 (up)	1,000	6.96×10^{-6}	3.13×10^{-6} to 2.82×10^{-5}
Table S9A	GSE249027 bulk, unadjusted	30	162	8.56×10^{-8}	1.14×10^{-5} (up)	3.74×10^{-8}	7.27×10^{-9}	3.70×10^{-6} (up)	1,000	8.28×10^{-9}	4.97×10^{-10} to 1.25×10^{-8}
Table S9A	GSE151243 bulk, unadjusted	40	164	9.75×10^{-11}	2.66×10^{-8} (up)	1.01×10^{-10}	2.59×10^{-14}	7.33×10^{-11} (up)	1,000	3.57×10^{-11}	5.50×10^{-12} to 4.77×10^{-11}
Table S9A	GSE154773 bulk, unadjusted	32	164	2.39×10^{-9}	0.00601 (up)	2.37×10^{-12}	7.57×10^{-12}	0.00154 (up)	1,000	7.40×10^{-12}	6.70×10^{-12} to 8.21×10^{-12}
Table S10B	GSE249027 bulk, composition-adjusted (Mono/Mac estimate M1)	30	162	1.22×10^{-5}	2.33×10^{-4} (up)	4.10×10^{-6}	1.72×10^{-6}	1.06×10^{-4} (up)	1,000	1.11×10^{-6}	5.59×10^{-8} to 1.68×10^{-6}
Table S10B	GSE151243 bulk, composition-adjusted (Mono/Mac estimate M1)	40	164	5.78×10^{-9}	3.61×10^{-5} (up)	2.52×10^{-8}	1.09×10^{-10}	3.42×10^{-6} (up)	1,000	6.63×10^{-9}	3.48×10^{-10} to 9.82×10^{-9}
Table S10C	GSE213761 adalimumab week 12 vs baseline (14 paired)	28	161	0.378	0.314 (up)	0.194	0.258	0.304 (up)	1,000	0.259	0.24 to 0.282
Table S10C	GSE189266 brodalumab week 12 vs baseline (9 vs 9, unpaired)	18	155	0.754	0.793 (up)	0.671	0.742	0.952 (up)	1,000	0.746	0.739 to 0.751
Table S8A	GSE212721 replication (6 HS vs 3 HC)	9	158	n.c.	n.c.	n.c.	0.0278	0.551 (down)	–	–	–
Table S8A	GSE220116 descriptive	6	162	n.c.	n.c.	n.c.	0.00653	0.0702 (up)	–	–	–

Order-invariant: limma::fry on voom log2 counts per million with the observation weights removed and standardize="none"; this value does not depend on the order of the samples and is the value reported in the main text and in Tables S6-S10. Standardised order-invariant: each gene divided by its residual standard deviation from the linear model before the same test. Weighted test: the default fry call on the voom object (observation weights, standardize="posterior.sd"), whose P value depends on the order of the samples; it is given for the original sample order and for random orderings (set.seed(1)). The first column gives the table in which the analysis is reported. n.c., not computable: for the two replication cohorts only the weighted result of the original analysis was archived (Table S8A). For the discovery endpoint, ROAST with 9999 rotations on the unweighted input gave a mixed-direction P of 2×10^{-4} (active proportion 0.69).

Table S12A. ER167 and established gene sets: Fry and matched-null results in the discovery and bulk cohorts

Main-text linkage: Methods 2.8; specificity Results; Figure S10.

Cohort	Gene set	Genes in set	Shared with ER167	Detected	Fry mixed P (order-invariant)	Fry P, BH across 14 sets	Observed mean t^2	Null mean	Null SD	z	Null percentile	Matched-null empirical P	Empirical P, BH across 14 sets
GSE249793 discovery scRNA-seq (4 HS vs 5 HC)	ER167 (full panel)	167	167	141	8.56×10^{-7}	–	16.10	8.58	1.20	6.27	100.00	1/10,001	–

Cohort	Gene set	Genes in set	Shared with ER167	Detected	Fry mixed P (order-invariant)	Fry P, BH across 14 sets	Observed mean t²	Null mean	Null SD	z	Null percentile	Matched-null empirical P	Empirical P, BH across 14 sets
GSE249793 discovery scRNA-seq (4 HS vs 5 HC)	ER167 without inflammatory/myeloid-context and uncategorized genes (A3)	106	106	91	2.08×10^{-6}	–	14.99	8.62	1.47	4.32	99.97	4/10,001	–
GSE249793 discovery scRNA-seq (4 HS vs 5 HC)	GO apoptotic cell clearance (GO:0043277)	44	5	23	0.00249	0.00291	17.82	8.28	2.76	3.45	99.63	0.0038	0.0252
GSE249793 discovery scRNA-seq (4 HS vs 5 HC)	GO phagocytosis (GO:0006909)	250	17	144	1.14×10^{-5}	2.29×10^{-5}	11.04	8.58	1.20	2.06	96.94	0.0307	0.086
GSE249793 discovery scRNA-seq (4 HS vs 5 HC)	GO phagocytosis, engulfment (GO:0006911)	48	4	30	0.00126	0.0016	8.92	8.99	2.77	−0.02	55.24	0.448	0.58
GSE249793 discovery scRNA-seq (4 HS vs 5 HC)	Reactome Fcγ receptor-dependent phagocytosis (R-HSA-2029480)	143	3	71	0.0525	0.0525	8.46	9.33	1.83	−0.48	33.93	0.661	0.712
GSE249793 discovery scRNA-seq (4 HS vs 5 HC)	KEGG lysosome (hsa04142)	121	6	83	5.35×10^{-7}	2.50×10^{-6}	9.86	8.66	1.60	0.75	78.55	0.215	0.376
GSE249793 discovery scRNA-seq (4 HS vs 5 HC)	GO phagosome maturation (GO:0090382)	31	1	19	4.79×10^{-5}	7.45×10^{-5}	9.60	8.84	3.17	0.24	65.54	0.345	0.536
GSE249793 discovery scRNA-seq (4 HS vs 5 HC)	Hallmark interferon-α response	97	1	65	3.30×10^{-8}	4.62×10^{-7}	9.22	9.16	1.78	0.03	54.45	0.456	0.58
GSE249793 discovery scRNA-seq (4 HS vs 5 HC)	Hallmark interferon-γ response	200	6	138	1.07×10^{-6}	3.74×10^{-6}	8.46	8.90	1.20	−0.37	38.22	0.618	0.712
GSE249793 discovery scRNA-seq (4 HS vs 5 HC)	Hallmark inflammatory response	200	22	140	1.25×10^{-7}	8.75×10^{-7}	16.01	8.69	1.19	6.13	100.00	1/10,001	0.0014
GSE249793 discovery scRNA-seq (4 HS vs 5 HC)	Hallmark TNF-α signaling via NF-κB	200	20	179	6.72×10^{-5}	9.40×10^{-5}	11.20	8.86	1.09	2.15	97.60	0.0241	0.0843
GSE249793 discovery scRNA-seq (4 HS vs 5 HC)	GO macrophage activation (GO:0042116)	119	11	67	1.15×10^{-5}	2.29×10^{-5}	11.79	8.67	1.78	1.75	94.47	0.0554	0.111
GSE249793 discovery scRNA-seq (4 HS vs 5 HC)	KEGG efferocytosis (hsa04148)	158	13	91	1.66×10^{-6}	4.66×10^{-6}	12.68	8.41	1.43	2.99	99.47	0.0054	0.0252
GSE249793 discovery scRNA-seq (4 HS vs 5 HC)	Manual general-inflammation panel	38	7	31	0.0117	0.0126	5.60	9.44	2.91	−1.32	5.40	0.946	0.946

Cohort	Gene set	Genes in set	Shared with ER167	Detected	Fry mixed P (order-invariant)	Fry P, BH across 14 sets	Observed mean t^2	Null mean	Null SD	z	Null percentile	Matched-null empirical P	Empirical P, BH across 14 sets
GSE249793 discovery scRNA-seq (4 HS vs 5 HC)	Manual myeloid-activation panel	38	20	36	2.57×10^{-5}	4.50×10^{-5}	13.15	8.90	2.34	1.82	94.71	0.053	0.111
GSE249027 bulk (paired L vs NL)	ER167 (full panel)	167	167	162	8.56×10^{-8}	–	32.84	17.26	1.77	8.79	100.00	1/10,001	–
GSE249027 bulk (paired L vs NL)	ER167 without inflammatory/myeloid-context and uncategorized genes (A3)	106	106	102	8.82×10^{-7}	–	34.73	16.27	2.17	8.51	100.00	1/10,001	–
GSE249027 bulk (paired L vs NL)	GO apoptotic cell clearance (GO:0043277)	44	5	42	9.41×10^{-8}	1.55×10^{-7}	23.94	16.49	3.37	2.21	97.71	0.023	0.0293
GSE249027 bulk (paired L vs NL)	GO phagocytosis (GO:0006909)	250	17	228	3.32×10^{-8}	8.68×10^{-8}	25.77	16.68	1.46	6.23	100.00	1/10,001	1.75×10^{-4}
GSE249027 bulk (paired L vs NL)	GO phagocytosis, engulfment (GO:0006911)	48	4	47	4.94×10^{-7}	6.28×10^{-7}	24.66	17.07	3.27	2.32	98.35	0.0166	0.0232
GSE249027 bulk (paired L vs NL)	Reactome Fcγ receptor-dependent phagocytosis (R-HSA-2029480)	143	3	142	6.96×10^{-7}	8.12×10^{-7}	74.23	48.13	3.26	8.00	100.00	1/10,001	1.75×10^{-4}
GSE249027 bulk (paired L vs NL)	KEGG lysosome (hsa04142)	121	6	119	8.80×10^{-7}	9.48×10^{-7}	18.57	17.27	2.05	0.64	74.49	0.255	0.255
GSE249027 bulk (paired L vs NL)	GO phagosome maturation (GO:0090382)	31	1	31	2.03×10^{-8}	7.09×10^{-8}	22.96	16.50	3.92	1.65	93.92	0.0609	0.071
GSE249027 bulk (paired L vs NL)	Hallmark interferon-α response	97	1	97	4.04×10^{-8}	8.68×10^{-8}	39.21	16.47	2.28	9.97	100.00	1/10,001	1.75×10^{-4}
GSE249027 bulk (paired L vs NL)	Hallmark interferon-γ response	200	6	200	2.07×10^{-7}	2.89×10^{-7}	35.01	16.83	1.63	11.12	100.00	1/10,001	1.75×10^{-4}
GSE249027 bulk (paired L vs NL)	Hallmark inflammatory response	200	22	195	9.99×10^{-8}	1.55×10^{-7}	29.07	17.73	1.63	6.95	100.00	1/10,001	1.75×10^{-4}
GSE249027 bulk (paired L vs NL)	Hallmark TNF-α signaling via NF-κB	200	20	199	4.34×10^{-8}	8.68×10^{-8}	24.94	17.12	1.60	4.87	100.00	1/10,001	1.75×10^{-4}
GSE249027 bulk (paired L vs NL)	GO macrophage activation (GO:0042116)	119	11	106	1.95×10^{-8}	7.09×10^{-8}	29.28	16.48	2.21	5.79	100.00	1/10,001	1.75×10^{-4}
GSE249027 bulk (paired L vs NL)	KEGG efferocytosis (hsa04148)	158	13	156	3.89×10^{-9}	5.45×10^{-8}	18.62	16.35	1.75	1.30	89.71	0.103	0.111
GSE249027 bulk (paired L vs NL)	Manual general-inflammation panel	38	7	38	9.43×10^{-9}	6.60×10^{-8}	40.53	25.28	4.86	3.14	99.73	0.0028	0.00436
GSE249027 bulk (paired L vs NL)	Manual myeloid-activation panel	38	20	37	5.08×10^{-6}	5.08×10^{-6}	39.49	18.26	4.18	5.09	100.00	1/10,001	1.75×10^{-4}
GSE151243 bulk (paired L vs perilesional)	ER167 (full panel)	167	167	164	9.75×10^{-11}	–	43.77	16.74	1.75	15.43	100.00	1/10,001	–
GSE151243 bulk (paired L vs perilesional)	ER167 without inflammatory/myeloid-context and uncategorized genes (A3)	106	106	104	1.87×10^{-10}	–	47.85	16.56	2.21	14.18	100.00	1/10,001	–

Cohort	Gene set	Genes in set	Shared with ER167	Detected	Fry mixed P (order-invariant)	Fry P, BH across 14 sets	Observed mean t²	Null mean	Null SD	z	Null percentile	Matched-null empirical P	Empirical P, BH across 14 sets
GSE151243 bulk (paired L vs perilesional)	GO apoptotic cell clearance (GO:0043277)	44	5	42	4.57×10^{-13}	6.40×10^{-12}	30.91	16.18	3.37	4.37	99.98	3/10,001	4.67×10^{-4}
GSE151243 bulk (paired L vs perilesional)	GO phagocytosis (GO:0006909)	250	17	237	2.20×10^{-11}	4.40×10^{-11}	32.77	15.87	1.40	12.11	100.00	1/10,001	1.75×10^{-4}
GSE151243 bulk (paired L vs perilesional)	GO phagocytosis, engulfment (GO:0006911)	48	4	48	7.62×10^{-12}	2.84×10^{-11}	36.49	16.48	3.22	6.21	100.00	1/10,001	1.75×10^{-4}
GSE151243 bulk (paired L vs perilesional)	Reactome Fcγ receptor-dependent phagocytosis (R-HSA-2029480)	143	3	143	8.74×10^{-8}	9.42×10^{-8}	39.88	18.17	1.87	11.61	100.00	1/10,001	1.75×10^{-4}
GSE151243 bulk (paired L vs perilesional)	KEGG lysosome (hsa04142)	121	6	122	1.48×10^{-11}	4.15×10^{-11}	20.04	16.56	2.00	1.74	95.45	0.0456	0.0456
GSE151243 bulk (paired L vs perilesional)	GO phagosome maturation (GO:0090382)	31	1	32	8.12×10^{-12}	2.84×10^{-11}	25.10	16.08	3.96	2.28	98.01	0.02	0.0233
GSE151243 bulk (paired L vs perilesional)	Hallmark interferon-α response	97	1	97	1.10×10^{-7}	1.10×10^{-7}	25.99	16.92	2.34	3.87	99.96	5/10,001	7.00×10^{-4}
GSE151243 bulk (paired L vs perilesional)	Hallmark interferon-γ response	200	6	199	3.20×10^{-9}	4.07×10^{-9}	31.21	16.85	1.63	8.79	100.00	1/10,001	1.75×10^{-4}
GSE151243 bulk (paired L vs perilesional)	Hallmark inflammatory response	200	22	197	3.17×10^{-9}	4.07×10^{-9}	32.96	16.87	1.57	10.25	100.00	1/10,001	1.75×10^{-4}
GSE151243 bulk (paired L vs perilesional)	Hallmark TNF-α signaling via NF-κB	200	20	199	9.11×10^{-9}	1.06×10^{-8}	24.71	16.89	1.60	4.88	100.00	1/10,001	1.75×10^{-4}
GSE151243 bulk (paired L vs perilesional)	GO macrophage activation (GO:0042116)	119	11	110	1.79×10^{-11}	4.17×10^{-11}	34.79	16.38	2.09	8.79	100.00	1/10,001	1.75×10^{-4}
GSE151243 bulk (paired L vs perilesional)	KEGG efferocytosis (hsa04148)	158	13	158	1.86×10^{-12}	1.30×10^{-11}	19.35	16.12	1.74	1.86	96.50	0.0351	0.0378
GSE151243 bulk (paired L vs perilesional)	Manual general-inflammation panel	38	7	38	2.56×10^{-9}	3.99×10^{-9}	29.75	19.33	3.66	2.84	99.42	0.0059	0.00751
GSE151243 bulk (paired L vs perilesional)	Manual myeloid-activation panel	38	20	38	2.90×10^{-10}	5.07×10^{-10}	56.45	16.58	3.69	10.80	100.00	1/10,001	1.75×10^{-4}
GSE154773 bulk (HS vs HC)	ER167 (full panel)	167	167	164	2.39×10^{-9}	–	13.63	9.22	1.00	4.42	99.99	2/10,001	–
GSE154773 bulk (HS vs HC)	ER167 without inflammatory/myeloid-context and uncategorized genes (A3)	106	106	104	3.50×10^{-9}	–	14.78	9.13	1.26	4.50	100.00	1/10,001	–
GSE154773 bulk (HS vs HC)	GO apoptotic cell clearance (GO:0043277)	44	5	41	1.39×10^{-13}	9.76×10^{-13}	10.84	9.20	2.05	0.80	79.86	0.201	0.247
GSE154773 bulk (HS vs HC)	GO phagocytosis (GO:0006909)	250	17	217	1.19×10^{-11}	5.56×10^{-11}	12.53	9.14	0.89	3.83	99.99	2/10,001	7.00×10^{-4}
GSE154773 bulk (HS vs HC)	GO phagocytosis, engulfment (GO:0006911)	48	4	45	6.11×10^{-9}	9.50×10^{-9}	13.49	9.53	1.98	1.99	96.58	0.0343	0.06

Cohort	Gene set	Genes in set	Shared with ER167	Detected	Fry mixed P (order-invariant)	Fry P, BH across 14 sets	Observed mean t²	Null mean	Null SD	z	Null percentile	Matched-null empirical P	Empirical P, BH across 14 sets
GSE154773 bulk (HS vs HC)	Reactome Fcγ receptor-dependent phagocytosis (R-HSA-2029480)	143	3	140	1.97×10^{-9}	3.93×10^{-9}	14.48	9.64	1.14	4.25	99.99	2/10,001	7.00×10^{-4}
GSE154773 bulk (HS vs HC)	KEGG lysosome (hsa04142)	121	6	117	2.57×10^{-9}	4.51×10^{-9}	13.86	9.77	1.27	3.22	99.81	0.002	0.0056
GSE154773 bulk (HS vs HC)	GO phagosome maturation (GO:0090382)	31	1	30	2.76×10^{-10}	9.67×10^{-10}	11.36	8.54	2.20	1.28	89.16	0.108	0.169
GSE154773 bulk (HS vs HC)	Hallmark interferon-α response	97	1	95	3.21×10^{-6}	3.45×10^{-6}	13.06	9.02	1.27	3.18	99.77	0.0024	0.0056
GSE154773 bulk (HS vs HC)	Hallmark interferon-γ response	200	6	196	1.75×10^{-9}	3.93×10^{-9}	12.93	8.90	0.89	4.52	100.00	1/10,001	7.00×10^{-4}
GSE154773 bulk (HS vs HC)	Hallmark inflammatory response	200	22	192	4.87×10^{-7}	5.68×10^{-7}	9.97	9.24	0.92	0.79	78.81	0.212	0.247
GSE154773 bulk (HS vs HC)	Hallmark TNF-α signaling via NF-κB	200	20	196	2.88×10^{-7}	3.67×10^{-7}	8.17	9.11	0.92	−1.03	15.61	0.844	0.909
GSE154773 bulk (HS vs HC)	GO macrophage activation (GO:0042116)	119	11	103	7.07×10^{-10}	1.98×10^{-9}	12.68	9.05	1.25	2.91	99.62	0.0039	0.0078
GSE154773 bulk (HS vs HC)	KEGG efferocytosis (hsa04148)	158	13	152	1.32×10^{-16}	1.84×10^{-15}	10.47	9.22	1.07	1.17	87.69	0.123	0.172
GSE154773 bulk (HS vs HC)	Manual general-inflammation panel	38	7	37	0.00276	0.00276	4.96	8.73	2.06	−1.83	1.67	0.983	0.983
GSE154773 bulk (HS vs HC)	Manual myeloid-activation panel	38	20	37	1.23×10^{-8}	1.73×10^{-8}	21.14	9.43	2.16	5.43	99.99	2/10,001	7.00×10^{-4}

Each gene set was compared with 10,000 random gene sets matched for size, expression level and detection in the same cohort; the statistic is the mean squared moderated t statistic. z=(observed − null mean)/null SD. Benjamini-Hochberg (BH) adjustment was applied within each cohort across the 14 comparator sets (12 established sets and the two manual panels); ER167 and set A3 were not included in the adjustment. Empirical P values of 5/10,001 or less are given as counts. In cohorts whose count matrix is indexed by Ensembl identifier, gene sets were matched by identifier; because a symbol can map to more than one identifier, the number of detected genes can exceed the number of symbols of the set (KEGG lysosome and GO phagosome maturation in GSE151243).

Table S12B. ER167 after exclusion of manually assigned functional categories

Main-text linkage: Methods 2.8; specificity Results; Figure S10.

Cohort	Gene set	Genes	Detected	Fry mixed P (order-invariant)	Fry directional P (direction)	Observed mean t²	Null mean	Null SD	z	Matched-null empirical P	Fry P, BH across the five single-category sets	Empirical P, BH across the five single-category sets
GSE249793 discovery scRNA-seq (4 HS vs 5 HC)	ER167 (full panel)	167	141	8.56×10^{-7}	0.479 (up)	16.10	8.58	1.20	6.27	1/10,001	–	–
GSE249793 discovery scRNA-seq (4 HS vs 5 HC)	A1: without inflammatory/myeloid context	122	102	4.21×10^{-6}	0.861 (down)	14.08	8.59	1.40	3.92	5/10,001	–	–
GSE249793 discovery scRNA-seq (4 HS vs 5 HC)	A2: without uncategorized	151	130	5.20×10^{-7}	0.316 (up)	16.91	8.57	1.25	6.69	1/10,001	–	–

Cohort	Gene set	Genes	Detected	Fry mixed P (order-invariant)	Fry directional P (direction)	Observed mean t^2	Null mean	Null SD	z	Matched-null empirical P	Fry P, BH across the five single-category sets	Empirical P, BH across the five single-category sets
GSE249793 discovery scRNA-seq (4 HS vs 5 HC)	A3: without inflammatory/myeloid context and uncategorized	106	91	2.08×10^{-6}	0.943 (up)	14.99	8.62	1.47	4.32	4/10,001	–	–
GSE249793 discovery scRNA-seq (4 HS vs 5 HC)	A4: without recognition/tethering	132	108	2.58×10^{-7}	0.272 (up)	15.96	8.62	1.39	5.29	2/10,001	–	–
GSE249793 discovery scRNA-seq (4 HS vs 5 HC)	A5: without engulfment/processing	137	112	3.28×10^{-7}	0.254 (up)	17.58	8.57	1.35	6.68	1/10,001	–	–
GSE249793 discovery scRNA-seq (4 HS vs 5 HC)	A6: without post-engulfment/resolution	126	112	4.99×10^{-6}	0.571 (up)	15.66	8.68	1.39	5.03	1/10,001	–	–
GSE249793 discovery scRNA-seq (4 HS vs 5 HC)	A7: without inflammatory/myeloid context (same set as A1)	122	102	4.21×10^{-6}	0.861 (down)	14.08	8.59	1.40	3.92	5/10,001	–	–
GSE249793 discovery scRNA-seq (4 HS vs 5 HC)	A8: without uncategorized (same set as A2)	151	130	5.20×10^{-7}	0.316 (up)	16.91	8.57	1.25	6.69	1/10,001	–	–
GSE249793 discovery scRNA-seq (4 HS vs 5 HC)	A9: recognition/tethering only	35	33	3.64×10^{-5}	0.987 (up)	16.57	8.94	2.57	2.97	0.0101	6.06×10^{-5}	0.0168
GSE249793 discovery scRNA-seq (4 HS vs 5 HC)	A9: engulfment/processing only	30	29	2.85×10^{-4}	0.599 (down)	10.37	8.82	2.74	0.57	0.244	3.56×10^{-4}	0.305
GSE249793 discovery scRNA-seq (4 HS vs 5 HC)	A9: post-engulfment/resolution only	41	29	5.05×10^{-8}	0.2 (up)	17.81	8.51	2.51	3.71	0.003	2.53×10^{-7}	0.0075
GSE249793 discovery scRNA-seq (4 HS vs 5 HC)	A9: inflammatory/myeloid context only	45	39	3.23×10^{-7}	0.00277 (up)	21.38	8.73	2.44	5.18	2/10,001	8.07×10^{-7}	1.00×10^{-3}
GSE249793 discovery scRNA-seq (4 HS vs 5 HC)	A9: uncategorized only	16	11	0.0222	0.086 (down)	6.58	8.44	4.27	−0.44	0.615	0.0222	0.615
GSE249027 bulk (paired L vs NL)	ER167 (full panel)	167	162	8.56×10^{-8}	1.14×10^{-5} (up)	32.84	17.26	1.77	8.79	1/10,001	–	–
GSE249027 bulk (paired L vs NL)	A3: without inflammatory/myeloid context and uncategorized	106	102	8.82×10^{-7}	3.68×10^{-5} (up)	34.73	16.27	2.17	8.51	1/10,001	–	–
GSE151243 bulk (paired L vs perilesional)	ER167 (full panel)	167	164	9.75×10^{-11}	2.66×10^{-8} (up)	43.77	16.74	1.75	15.43	1/10,001	–	–
GSE151243 bulk (paired L vs perilesional)	A3: without inflammatory/myeloid context and uncategorized	106	104	1.87×10^{-10}	2.63×10^{-8} (up)	47.85	16.56	2.21	14.18	1/10,001	–	–
GSE154773 bulk (HS vs HC)	ER167 (full panel)	167	164	2.39×10^{-9}	0.00601 (up)	13.63	9.22	1.00	4.42	2/10,001	–	–
GSE154773 bulk (HS vs HC)	A3: without inflammatory/myeloid context and uncategorized	106	104	3.50×10^{-9}	0.00113 (up)	14.78	9.13	1.26	4.50	1/10,001	–	–

Category assignments are those of Table S1. Set A3 could not be tested in the GSE212721 replication cohort, because the expression object of that analysis was not archived. The single-category sets (A9) were compared here with a draw of 10,000 random gene sets that is independent of the draw used for Table S6B, so that the empirical P values of the two tables differ slightly (post-engulfment/resolution 0.003 against 0.0022; engulfment/processing 0.244 against 0.247; uncategorized 0.615 against 0.62); the main text reports the values of Table S6B.

Table S12C. ER167 after removal of genes shared with established gene sets

Main-text linkage: Methods 2.8; specificity Results.

Cohort	Gene set	Genes	Detected	Fry mixed P (order-invariant)	Fry directional P (direction)	Observed mean t^2	Null mean	Null SD	z	Null percentile	Matched-null empirical P
GSE249793 discovery scRNA-seq (4 HS vs 5 HC)	B1: ER167 without genes of the Hallmark inflammatory response and TNF-α/NF-κB sets	133	108	1.12×10^{-6}	0.541 (up)	13.80	8.81	1.43	3.48	99.85	0.0016
GSE249793 discovery scRNA-seq (4 HS vs 5 HC)	B2: ER167 without genes of the Hallmark interferon-α and interferon-γ sets	160	134	1.12×10^{-6}	0.79 (up)	16.13	8.60	1.25	6.03	100.00	1/10,001
GSE249793 discovery scRNA-seq (4 HS vs 5 HC)	B3: ER167 without genes of GO macrophage activation	156	130	5.33×10^{-7}	0.733 (up)	15.25	8.63	1.26	5.24	100.00	1/10,001
GSE249793 discovery scRNA-seq (4 HS vs 5 HC)	B4: ER167 without genes of the phagocytosis, lysosome and phagosome sets	144	120	4.15×10^{-7}	0.572 (up)	16.16	8.65	1.31	5.74	100.00	1/10,001
GSE249793 discovery scRNA-seq (4 HS vs 5 HC)	B5: ER167 without genes of any of the ten non-efferocytosis sets	98	76	1.35×10^{-6}	0.926 (up)	13.77	8.84	1.72	2.87	99.19	0.0082
GSE249793 discovery scRNA-seq (4 HS vs 5 HC)	B6: ER167 genes present in GO apoptotic cell clearance or KEGG efferocytosis	17	15	0.0295	0.0288 (down)	11.48	8.34	3.15	1.00	84.72	0.153
GSE249027 bulk (paired L vs NL)	B1: ER167 without genes of the Hallmark inflammatory response and TNF-α/NF-κB sets	133	128	2.28×10^{-7}	2.32×10^{-5} (up)	32.48	17.51	2.04	7.33	100.00	1/10,001
GSE249027 bulk (paired L vs NL)	B2: ER167 without genes of the Hallmark interferon-α and interferon-γ sets	160	155	1.33×10^{-7}	1.66×10^{-5} (up)	32.51	17.21	1.80	8.50	100.00	1/10,001
GSE249027 bulk (paired L vs NL)	B3: ER167 without genes of GO macrophage activation	156	151	7.52×10^{-8}	1.19×10^{-5} (up)	32.62	17.21	1.85	8.34	100.00	1/10,001
GSE249027 bulk (paired L vs NL)	B4: ER167 without genes of the phagocytosis, lysosome and phagosome sets	144	140	4.19×10^{-8}	1.07×10^{-5} (up)	32.08	17.38	1.91	7.70	100.00	1/10,001
GSE249027 bulk (paired L vs NL)	B5: ER167 without genes of any of the ten non-efferocytosis sets	98	94	5.51×10^{-7}	4.00×10^{-5} (up)	29.60	17.33	2.34	5.24	100.00	1/10,001
GSE249027 bulk (paired L vs NL)	B6: ER167 genes present in GO apoptotic cell clearance or KEGG efferocytosis	17	17	3.95×10^{-7}	4.18×10^{-6} (up)	42.49	17.22	5.38	4.69	99.98	3/10,001
GSE151243 bulk (paired L vs perilesional)	B1: ER167 without genes of the Hallmark inflammatory response and TNF-α/NF-κB sets	133	130	1.55×10^{-11}	1.16×10^{-8} (up)	44.32	16.43	1.98	14.06	100.00	1/10,001
GSE151243 bulk (paired L vs perilesional)	B2: ER167 without genes of the Hallmark interferon-α and interferon-γ sets	160	157	5.22×10^{-11}	2.19×10^{-8} (up)	43.63	16.51	1.79	15.11	100.00	1/10,001
GSE151243 bulk (paired L vs perilesional)	B3: ER167 without genes of GO macrophage activation	156	153	4.68×10^{-11}	2.43×10^{-8} (up)	42.61	16.50	1.80	14.48	100.00	1/10,001
GSE151243 bulk (paired L vs perilesional)	B4: ER167 without genes of the phagocytosis, lysosome and phagosome sets	144	141	9.96×10^{-11}	4.40×10^{-8} (up)	41.42	16.89	1.90	12.94	100.00	1/10,001
GSE151243 bulk (paired L vs perilesional)	B5: ER167 without genes of any of the ten non-efferocytosis sets	98	95	1.05×10^{-11}	2.01×10^{-8} (up)	41.27	16.67	2.33	10.56	100.00	1/10,001
GSE151243 bulk (paired L vs perilesional)	B6: ER167 genes present in GO apoptotic cell clearance or KEGG efferocytosis	17	17	1.19×10^{-8}	6.55×10^{-8} (up)	44.29	16.38	5.43	5.14	99.98	3/10,001
GSE154773 bulk (HS vs HC)	B1: ER167 without genes of the Hallmark inflammatory response and TNF-α/NF-κB sets	133	130	1.05×10^{-9}	2.44×10^{-4} (up)	14.76	9.22	1.13	4.89	100.00	1/10,001

Cohort	Gene set	Genes	Detected	Fry mixed P (order-invariant)	Fry directional P (direction)	Observed mean t^2	Null mean	Null SD	z	Null percentile	Matched-null empirical P
GSE154773 bulk (HS vs HC)	B2: ER167 without genes of the Hallmark interferon-α and interferon-γ sets	160	157	2.79×10^{-9}	0.00372 (up)	13.64	9.22	1.03	4.31	100.00	1/10,001
GSE154773 bulk (HS vs HC)	B3: ER167 without genes of GO macrophage activation	156	153	1.12×10^{-9}	0.00623 (up)	13.51	9.23	1.05	4.06	99.98	3/10,001
GSE154773 bulk (HS vs HC)	B4: ER167 without genes of the phagocytosis, lysosome and phagosome sets	144	143	4.88×10^{-10}	0.0107 (up)	13.75	9.22	1.09	4.14	100.00	1/10,001
GSE154773 bulk (HS vs HC)	B5: ER167 without genes of any of the ten non-efferocytosis sets	98	97	1.97×10^{-8}	0.00129 (up)	13.15	9.24	1.34	2.92	99.64	0.0037
GSE154773 bulk (HS vs HC)	B6: ER167 genes present in GO apoptotic cell clearance or KEGG efferocytosis	17	16	2.44×10^{-5}	0.0136 (up)	20.54	9.71	3.34	3.25	99.64	0.0037

Table S12D. Definitions and sources of the gene sets compared with ER167

Main-text linkage: Methods 2.8 and 2.13.

Gene set	Set name in the source	Source	Collection	Systematic identifier	Ontology or pathway identifier	Database version	Genes
GO apoptotic cell clearance (GO:0043277)	GOBP_APOPTOTIC_CELL_CLEARANCE	MSigDB (msigdbr 26.1.1)	C5:GO:BP	M11346	GO:0043277	2026.1.Hs	44
GO phagocytosis (GO:0006909)	GOBP_PHAGOCYTOSIS	MSigDB (msigdbr 26.1.1)	C5:GO:BP	M16307	GO:0006909	2026.1.Hs	250
GO phagocytosis, engulfment (GO:0006911)	GOBP_PHAGOCYTOSIS_ENGULFMENT	MSigDB (msigdbr 26.1.1)	C5:GO:BP	M45812	GO:0006911	2026.1.Hs	48
Reactome Fcγ receptor-dependent phagocytosis (R-HSA-2029480)	REACTOME_FCGAMMA_RECEPTOR_FCGR_DEPENDENT_PHAGOCYTOSIS	MSigDB (msigdbr 26.1.1)	C2:CP:REACTOME	M27107	R-HSA-2029480	2026.1.Hs	143
KEGG lysosome (hsa04142)	KEGG_LYSOSOME	MSigDB (msigdbr 26.1.1)	C2:CP:KEGG_LEGACY	M11266	hsa04142	2026.1.Hs	121
GO phagosome maturation (GO:0090382)	GOBP_PHAGOSOME_MATURATION	MSigDB (msigdbr 26.1.1)	C5:GO:BP	M12757	GO:0090382	2026.1.Hs	31
Hallmark interferon-α response	HALLMARK_INTERFERON_ALPHA_RESPONSE	MSigDB (msigdbr 26.1.1)	H	M5911	–	2026.1.Hs	97
Hallmark interferon-γ response	HALLMARK_INTERFERON_GAMMA_RESPONSE	MSigDB (msigdbr 26.1.1)	H	M5913	–	2026.1.Hs	200
Hallmark inflammatory response	HALLMARK_INFLAMMATORY_RESPONSE	MSigDB (msigdbr 26.1.1)	H	M5932	–	2026.1.Hs	200
Hallmark TNF-α signaling via NF-κB	HALLMARK_TNFA_SIGNALING_VIA_NFKB	MSigDB (msigdbr 26.1.1)	H	M5890	–	2026.1.Hs	200
GO macrophage activation (GO:0042116)	GOBP_MACROPHAGE_ACTIVATION	MSigDB (msigdbr 26.1.1)	C5:GO:BP	M13410	GO:0042116	2026.1.Hs	119
KEGG efferocytosis (hsa04148)	KEGG pathway hsa04148 Efferocytosis	KEGG (KEGGREST 1.50.0)	KEGG PATHWAY	hsa04148	hsa04148	KEGG, accessed 3 October 2026	158
Manual general-inflammation panel	Curated manually by the authors	Authors (gene list in Table S6D)	–	–	–	–	38
Manual myeloid-activation panel	Curated manually by the authors	Authors (gene list in Table S6D)	–	–	–	–	38

Table S13A. Gene Ontology and KEGG annotation of the 167 panel genes

Main-text linkage: Methods 2.2; functional-category Results.

Gene	Manual category	GO:0043277 apoptotic cell clearance	GO:0006909 phagocytosis	GO:0006911 phagocytosis, engulfment	GO:0090382 phagosome maturation	GO:0005764 lysosome	GO:0006954 inflammatory response	GO:0002274 myeloid leukocyte activation	KEGG hsa04148 efferocytosis
S100P	inflammatory myeloid context	–	–	–	–	–	–	–	–
TFF3	uncategorized	–	–	–	–	–	–	–	–
TESC	uncategorized	–	–	–	–	–	–	–	–
SAA1	inflammatory myeloid context	–	–	–	–	–	IEA, NAS	–	–
SPP1	inflammatory myeloid context	–	–	–	–	–	–	–	–
S100A9	inflammatory myeloid context	–	–	–	–	–	IBA, IDA, IEA, NAS, TAS	–	–
IGFBP2	uncategorized	–	–	–	–	–	–	–	–
FOSB	inflammatory myeloid context	–	–	–	–	–	–	–	–
MARCO	recognition tethering	IEA	IEA, ISS	IEA, ISS	–	–	–	–	–
ALDH1A1	post engulfment resolution	–	–	–	–	–	–	–	–
MMP9	inflammatory myeloid context	–	–	–	–	–	TAS	–	–
LYZ	inflammatory myeloid context	–	–	–	–	TAS	TAS	–	–
PLAU	inflammatory myeloid context	–	–	–	–	–	–	–	–
CCL18	inflammatory myeloid context	–	–	–	–	–	IBA, IEA	–	–
DUSP4	inflammatory myeloid context	–	–	–	–	–	–	–	yes
MRC1	recognition tethering	–	–	–	–	–	–	–	–
BASP1	engulfment processing	–	–	–	–	–	–	–	–
OLR1	recognition tethering	–	–	–	–	–	IEA	–	–
H1-2	uncategorized	–	–	–	–	–	–	–	–
VSIG4	recognition tethering	–	–	–	–	–	–	IEA	–
VCAN	inflammatory myeloid context	–	–	–	–	IEA, TAS	–	–	–
C1QB	recognition tethering	–	–	–	–	–	–	–	yes
CFD	recognition tethering	–	–	–	–	–	–	–	–
APOC1	post engulfment resolution	–	–	–	–	–	–	–	–
MCEMP1	inflammatory myeloid context	–	–	–	–	–	–	–	–
C1QC	recognition tethering	–	–	–	–	–	–	–	yes
CAV1	inflammatory myeloid context	–	–	–	–	–	–	–	–
CD52	recognition tethering	–	–	–	–	–	–	–	–

Gene	Manual category	GO:0043277 apoptotic cell clearance	GO:0006909 phagocytosis	GO:0006911 phagocytosis, engulfment	GO:0090382 phagosome maturation	GO:0005764 lysosome	GO:0006954 inflammatory response	GO:0002274 myeloid leukocyte activation	KEGG hsa04148 efferocytosis
C1QA	recognition tethering	–	–	–	–	–	IEA, ISS	IEA, ISS	yes
ITGB8	engulfment processing	–	–	–	–	–	–	IEA	–
CYBB	engulfment processing	–	–	–	–	–	IEA, ISS, TAS	–	–
FCGR3A	recognition tethering	–	–	–	–	–	IBA, IDA	IDA	–
COLEC12	recognition tethering	–	IBA, IDA	–	–	–	–	–	–
PER1	inflammatory myeloid context	–	–	–	–	–	ISS	–	–
KLF4	post engulfment resolution	–	–	–	–	–	TAS	–	–
CD163	recognition tethering	–	–	–	–	–	IEA	–	–
IL6	inflammatory myeloid context	–	–	–	–	–	IBA, IDA, IEA, IGI, TAS	TAS	–
RETN	post engulfment resolution	–	–	–	–	TAS	–	–	–
CYP27A1	post engulfment resolution	–	–	–	–	–	–	–	–
MNDA	engulfment processing	–	–	–	–	TAS	–	–	–
MSR1	recognition tethering	–	IEA, ISS	IEA, ISS	–	–	–	–	–
A2M	recognition tethering	–	–	–	–	–	–	–	–
RBP4	post engulfment resolution	–	–	–	–	–	–	–	–
ASPH	uncategorized	–	–	–	–	–	–	–	–
ODC1	post engulfment resolution	–	–	–	–	–	–	–	yes
CCL2	inflammatory myeloid context	ISS	ISS	–	–	–	IBA, IEA	–	–
PLBD1	engulfment processing	–	–	–	–	IDA, IEA, ISS	–	–	–
TYROBP	engulfment processing	IEA, ISS	IEA, ISS	–	–	–	IBA, IEA, ISS	IBA, IDA, IEA, ISS	–
EMB	engulfment processing	–	–	–	–	–	–	–	–
RGS16	inflammatory myeloid context	–	–	–	–	–	–	–	–
MMP14	inflammatory myeloid context	–	–	–	–	–	–	–	–
PPARG	post engulfment resolution	–	–	–	–	–	IBA, ISS	–	yes
OSCAR	recognition tethering	–	–	–	–	–	–	–	–
BCL2A1	inflammatory myeloid context	–	–	–	–	–	–	–	–
FABP5	post engulfment resolution	–	–	–	–	TAS	–	–	–
SIGLEC1	recognition tethering	–	–	–	–	–	NAS	–	–
SPI1	inflammatory myeloid context	–	–	–	–	–	–	IEA, ISS	–

Gene	Manual category	GO:0043277 apoptotic cell clearance	GO:0006909 phagocytosis	GO:0006911 phagocytosis, engulfment	GO:0090382 phagosome maturation	GO:0005764 lysosome	GO:0006954 inflammatory response	GO:0002274 myeloid leukocyte activation	KEGG hsa04148 efferocytosis
AQP9	inflammatory myeloid context	–	–	–	–	–	–	–	–
HCK	engulfment processing	–	IEA, IMP, TAS	–	–	IDA, IEA	IEA, TAS	–	–
ALOX5	post engulfment resolution	–	–	–	–	–	IDA, IEA, IMP, ISS	–	yes
PTAFR	recognition tethering	–	–	–	–	–	IEA, TAS	–	–
SLCO2B1	post engulfment resolution	–	–	–	–	–	–	–	–
MS4A7	inflammatory myeloid context	–	–	–	–	–	–	–	–
FABP4	post engulfment resolution	–	–	–	–	–	IEA	–	–
LY86	recognition tethering	–	–	–	–	–	IEA	–	–
BHLHE41	post engulfment resolution	–	–	–	–	–	–	–	–
FCER1G	recognition tethering	–	IBA, IEA	IEA	–	–	IEA	IBA, IEA	–
MS4A4A	recognition tethering	–	–	–	–	–	–	–	–
FPR1	recognition tethering	–	–	–	–	TAS	IBA	–	–
SOCS3	post engulfment resolution	–	–	–	–	–	IEA	–	–
TUBB6	engulfment processing	–	–	–	–	–	–	–	–
CSTA	inflammatory myeloid context	–	–	–	–	–	–	–	–
NCF2	engulfment processing	–	IEA	–	–	TAS	–	–	–
RGCC	inflammatory myeloid context	–	–	–	–	–	–	–	–
KCTD12	inflammatory myeloid context	–	–	–	–	–	–	–	–
PAPSS2	post engulfment resolution	–	–	–	–	–	–	–	–
BATF	inflammatory myeloid context	–	–	–	–	–	–	IEA, ISS	–
HCST	engulfment processing	–	–	–	–	–	–	–	–
CD37	recognition tethering	–	–	–	–	–	–	IEA	–
LST1	engulfment processing	–	–	–	–	–	–	–	–
THBD	recognition tethering	–	–	–	–	–	–	–	–
FBP1	post engulfment resolution	–	–	–	–	–	–	–	–
HBEGF	inflammatory myeloid context	–	–	–	–	–	–	–	–
EMP1	inflammatory myeloid context	–	–	–	–	–	–	–	–
CD83	inflammatory myeloid context	–	–	–	–	–	–	–	–
C5AR1	recognition tethering	–	–	–	–	–	IBA, IEA, ISS	IEA, ISS	–
FERMT3	engulfment processing	–	–	–	–	–	–	–	–

Gene	Manual category	GO:0043277 apoptotic cell clearance	GO:0006909 phagocytosis	GO:0006911 phagocytosis, engulfment	GO:0090382 phagosome maturation	GO:0005764 lysosome	GO:0006954 inflammatory response	GO:0002274 myeloid leukocyte activation	KEGG hsa04148 efferocytosis
SERPING1	post engulfment resolution	–	–	–	–	–	–	–	–
FBXO32	post engulfment resolution	–	–	–	–	–	–	–	–
SDS	post engulfment resolution	–	–	–	–	–	–	–	–
ACP5	post engulfment resolution	–	–	–	–	IEA	IEA	–	–
LAPTM5	engulfment processing	–	–	–	–	IBA, IDA, IEA	–	–	–
IER5L	inflammatory myeloid context	–	–	–	–	–	–	–	–
LAIR1	recognition tethering	–	–	–	–	–	–	–	–
CD93	recognition tethering	–	NAS	–	–	–	–	NAS	–
PCOLCE2	post engulfment resolution	–	–	–	–	–	–	–	–
PILRA	recognition tethering	–	–	–	–	–	–	–	–
SGK1	post engulfment resolution	–	–	–	–	–	–	–	yes
FGR	engulfment processing	–	IEA, ISS, TAS	–	–	–	IEA, TAS	IEA, ISS	–
LIPA	post engulfment resolution	–	–	–	–	IEA, ISS, TAS	IEA	–	yes
VIM	engulfment processing	–	–	–	–	–	–	–	–
PTGER4	post engulfment resolution	–	–	–	–	–	IBA, IDA, ISS	–	yes
IL1B	inflammatory myeloid context	–	–	–	–	IEA	IBA, IDA, IEA, IGI, ISS, NAS, TAS	–	–
JUNB	inflammatory myeloid context	–	–	–	–	–	–	–	–
FCN1	inflammatory myeloid context	IDA	IDA	–	–	–	–	–	–
GLIPR2	post engulfment resolution	–	–	–	–	–	–	–	–
EMP3	inflammatory myeloid context	–	–	–	–	–	–	–	–
ITGAX	engulfment processing	–	–	–	–	–	–	–	–
SLC15A3	post engulfment resolution	–	–	–	–	HDA, IBA, IEA, TAS	–	–	–
C1orf162	uncategorized	–	–	–	–	–	–	–	–
SLC7A7	post engulfment resolution	–	–	–	–	–	–	–	–
LBH	inflammatory myeloid context	–	–	–	–	–	–	–	–
SAMHD1	post engulfment resolution	–	–	–	–	–	–	–	–
CD36	recognition tethering	IEA	IEA, TAS	IEA, TAS	–	–	IEA, ISS, TAS	–	yes
SLC11A1	post engulfment resolution	–	IEA, ISS	–	–	IBA, IEA, ISS	IEA, ISS	IEA, ISS	–

Gene	Manual category	GO:0043277 apoptotic cell clearance	GO:0006909 phagocytosis	GO:0006911 phagocytosis, engulfment	GO:0090382 phagosome maturation	GO:0005764 lysosome	GO:0006954 inflammatory response	GO:0002274 myeloid leukocyte activation	KEGG hsa04148 efferocytosis
TLR4	recognition tethering	–	IDA	–	–	–	IBA, IDA, IEA, IMP, ISS	IEA, IMP, ISS	–
SIRPA	recognition tethering	–	IBA, ISS	–	–	–	IMP, ISS	–	yes
TIMP1	post engulfment resolution	–	–	–	–	–	IEA	–	–
DAB2	engulfment processing	–	–	–	–	TAS	–	–	–
CYRIA	uncategorized	–	–	–	–	–	–	–	–
PMP22	uncategorized	–	–	–	–	–	–	–	–
TTYH3	uncategorized	–	–	–	–	–	–	–	–
CAT	post engulfment resolution	–	–	–	–	–	–	–	–
ABCG1	post engulfment resolution	–	–	–	–	–	–	–	–
THEMIS2	engulfment processing	–	–	–	–	–	IEA	–	–
SLA	engulfment processing	–	–	–	–	–	–	–	–
MMP19	inflammatory myeloid context	–	–	–	–	–	–	–	–
STX11	engulfment processing	–	–	–	–	–	–	–	–
ENG	inflammatory myeloid context	–	–	–	–	–	–	–	–
PIK3IP1	post engulfment resolution	–	–	–	–	–	–	–	–
ARRDC4	post engulfment resolution	–	–	–	–	–	–	–	–
JUND	inflammatory myeloid context	–	–	–	–	–	–	IEA	–
ACE	inflammatory myeloid context	–	–	–	–	IDA	–	–	–
PECAM1	recognition tethering	–	IDA, IEA	–	–	–	–	–	yes
CCRL2	inflammatory myeloid context	–	–	–	–	–	IBA, ISS	–	–
TNS3	engulfment processing	–	–	–	–	–	–	–	–
PDIA4	uncategorized	–	–	–	–	–	–	–	–
EPB41L3	engulfment processing	–	–	–	–	–	–	–	–
SH3BP5	engulfment processing	–	–	–	–	–	–	–	–
CXCL16	inflammatory myeloid context	–	–	–	–	–	–	–	–
ARHGAP18	engulfment processing	–	–	–	–	–	–	–	–
EMILIN2	uncategorized	–	–	–	–	–	–	–	–
TTC39C	uncategorized	–	–	–	–	–	–	–	–
MOB3B	uncategorized	–	–	–	–	–	–	–	–

Gene	Manual category	GO:0043277 apoptotic cell clearance	GO:0006909 phagocytosis	GO:0006911 phagocytosis, engulfment	GO:0090382 phagosome maturation	GO:0005764 lysosome	GO:0006954 inflammatory response	GO:0002274 myeloid leukocyte activation	KEGG hsa04148 efferocytosis
GPR65	recognition tethering	–	–	–	–	–	–	–	–
IL3RA	inflammatory myeloid context	–	–	–	–	–	–	–	–
SMAD7	post engulfment resolution	–	–	–	–	–	–	–	–
QKI	post engulfment resolution	–	–	–	–	–	–	–	–
C1orf56	uncategorized	–	–	–	–	–	–	–	–
RCAN1	post engulfment resolution	–	–	–	–	–	–	–	–
PDLIM1	engulfment processing	–	–	–	–	–	–	–	–
DOCK4	engulfment processing	–	–	–	–	–	–	–	–
PDIA6	uncategorized	–	–	–	–	–	–	–	–
EREG	inflammatory myeloid context	–	–	–	–	–	–	–	–
MATK	engulfment processing	–	–	–	–	–	–	–	–
CD300E	recognition tethering	–	–	–	–	–	–	–	–
MITF	inflammatory myeloid context	–	–	–	–	IDA, IEA	–	–	–
OTUD1	post engulfment resolution	–	–	–	–	–	–	–	–
GLDN	uncategorized	–	–	–	–	–	–	–	–
PNPLA6	post engulfment resolution	–	–	–	–	–	–	–	–
CD68	recognition tethering	–	–	–	–	IBA, IEA, ISS, TAS	ISS	–	–
LILRB3	recognition tethering	–	–	–	–	–	–	–	–
SLC16A6	post engulfment resolution	–	–	–	–	–	–	–	–
MCOLN1	engulfment processing	–	–	–	IDA, IEA	HDA, IBA, IDA, IEA	–	–	–
SLC31A2	post engulfment resolution	–	–	–	–	IDA, IEA	–	–	–
FRMD4A	engulfment processing	–	–	–	–	–	–	–	–
IL1A	inflammatory myeloid context	–	–	–	–	–	IBA, IEA	–	–

Annotations were taken from org.Hs.eg.db 3.22.0 (Gene Ontology, including annotations to descendant terms) and from KEGG (hsa04148; accessed 3 October 2026). Cells give the Gene Ontology evidence codes of the annotation; –, not annotated. Seventeen of the 167 genes are annotated to GO:0043277 or hsa04148.

Table S13B. Agreement between the manually assigned categories and ontology annotation

Main-text linkage: Methods 2.2; functional-category Results.

Manual category	Genes	Ontology terms taken as supporting the category	Genes with a supporting annotation	%	Genes per supporting term	Genes in GO:0043277 or hsa04148

Manual category	Genes	Ontology terms taken as supporting the category	Genes with a supporting annotation	%	Genes per supporting term	Genes in GO:0043277 or hsa04148
recognition tethering	35	GO:0043277; GO:0006909; GO:0006911	9	25.7	GO:0043277: 2; GO:0006909: 9; GO:0006911: 4	7
engulfment processing	30	GO:0043277; GO:0006909; GO:0006911	4	13.3	GO:0043277: 1; GO:0006909: 4; GO:0006911: 0	1
post engulfment resolution	41	GO:0090382; GO:0005764; GO:0043277	7	17.1	GO:0090382: 0; GO:0005764: 7; GO:0043277: 0	6
inflammatory myeloid context	45	GO:0006954; GO:0002274	14	31.1	GO:0006954: 11; GO:0002274: 4	3
uncategorized	16	–	–	–	–	0
All genes	167	–	–	–	–	17

The supporting terms were chosen by the authors as the closest ontology counterparts of each manual category; no supporting term was defined for the uncategorized genes.

Table S13C. Number of genes of each manual category annotated to each ontology term

Main-text linkage: Methods 2.2; functional-category Results.

Manual category	GO:0043277	GO:0006909	GO:0006911	GO:0090382	GO:0005764	GO:0006954	GO:0002274	KEGG hsa04148
engulfment processing	1	4	0	1	7	5	3	0
inflammatory myeloid context	2	2	0	0	5	11	4	1
post engulfment resolution	0	1	0	0	7	10	1	6
recognition tethering	2	9	4	0	2	14	8	6
uncategorized	0	0	0	0	0	0	0	0

Table S14A. Composition of the single-cell reference used for deconvolution (GSE274880)

Main-text linkage: Methods 2.11; Table S10A.

Library	Status	Cells	B/plasma	Endothelial	Fibroblast	'Keratinocyte' (see note)	Mono/Mac	NK	Sweat gland	T
GSM8459837_Control_12012020	control	342	8	87	17	1	25	90	2	112
GSM8459838_Control_121420S	control	128	0	20	4	0	3	34	18	49
GSM8459839_HS_BA	HS	1,599	7	80	79	2	94	775	9	553
GSM8459840_HS_1292021	HS	5,986	1,060	271	269	73	106	510	623	3,074
GSM8459841_HS_2521	HS	7,143	1,357	59	231	55	62	484	62	4,833
GSM8459842_HS_73120	HS	609	83	158	30	0	2	142	6	188
GSM8459843_HS_G	HS	2,940	5	962	250	4	92	160	9	1,458
GSM8459844_HS_P	HS	2,631	160	377	518	86	152	159	6	1,173
All libraries	–	21,378	2,680 (12.5%)	2,014 (9.4%)	1,398 (6.5%)	221 (1.0%)	536 (2.5%)	2,354 (11.0%)	735 (3.4%)	11,440 (53.5%)

Cells per class in each of the eight libraries of the reference. The class labels are those assigned by cluster-level marker scoring in the original analysis and were not changed; the class labelled keratinocyte is not keratinocyte-like (Table S14B).

Table S14B. Most specific genes and canonical markers of the reference classes

Main-text linkage: Methods 2.11.

Class label	Cells	Libraries with ≥10 cells	Fifteen most specific genes (log2 fold-change versus the mean of the other classes)	KRT14	KRT5	KRT1	PTPRC	CD3E	CD79A	LYZ	CD14	CD68	PECAM1	COL1A1	DCD	NKG7	CD207	CD1A
B/plasma	2,680	4	IGLC1 (10.36); IGHG1 (8.94); IGHGP (8.60); IGLC2 (8.46); IGHG2 (8.24); IGLC3 (8.14); IGKC (8.04); IGHV3-7 (7.86); IGHG3 (7.64); IGHG4 (7.43); IGLV2-8 (7.32); IGLV3-1 (6.56); IGKV4-1 (6.53); IGHD (6.46); CD79A (6.45)	0.1	0.2	0.1	75.1	4.6	89.6	1.0	0.2	5.5	12.2	3.2	0.6	4.2	0.0	0.3
Endothelial	2,014	8	CCL21 (8.98); CCL14 (8.41); CLDN5 (8.30); MMRN1 (8.19); ACKR1 (8.05); SELE (7.83); ECSCR (7.76); VWF (7.74); LYVE1 (7.72); PROX1 (6.98); SOX18 (6.93); TSPAN7 (6.90); MYCT1 (6.79); PCAT19 (6.79); CAVIN2 (6.77)	0.0	0.1	0.0	3.8	2.1	0.5	1.1	7.2	6.3	82.0	10.2	0.9	1.4	0.0	0.0
Fibroblast	1,398	7	SFRP2 (9.60); DCN (9.53); COL1A1 (9.43); COL1A2 (9.37); LUM (9.36); CCN5 (9.24); COL3A1 (9.23); MMP3 (9.23); CXCL14 (8.98); MFAP5 (8.95); FBLN1 (8.54); MMP1 (8.45); COL14A1 (8.41); PRRX1 (8.11); OGN (8.09)	0.1	0.4	0.0	6.8	3.9	1.3	2.1	15.3	27.3	2.1	98.2	1.1	2.8	0.0	0.0
'Keratinocyte' (see note)	221	3	SCT (7.52); LILRA4 (7.50); KRT5 (7.46); PTCRA (7.20); LAMP5 (7.17); SHD (6.96); SMPD3 (6.79); CLEC4C (6.78); PLD4 (6.60); SLC32A1 (6.33); PROC (6.26); PACSIN1 (6.25); LINC02812 (6.04); MYBL2 (5.96); DRD4 (5.92)	0.5	63.3	0.0	84.6	14.0	6.8	2.3	0.5	76.5	43.0	12.7	1.4	29.0	0.0	0.0
Mono/Mac	536	6	LYZ (9.60); FCN1 (8.34); IL1B (8.23); C5AR1 (8.18); C1QB (7.86); C1QA (7.69); EREG (7.64); CLEC10A (7.57); APOBEC3A (7.46); CCL17 (7.43); AC020656.1 (7.36); CLEC7A (7.18); LGALS2 (7.09); FPR1 (6.99); SERPINA1 (6.72)	0.0	0.2	0.4	87.9	2.2	0.9	88.2	45.0	63.2	37.5	10.3	1.5	11.2	2.2	4.5
NK	2,354	8	FGFBP2 (7.04); KLRF1 (6.85); KLRC1 (6.40); GNLY (6.16); GZMH (6.00); KLRD1 (5.98); NKG7 (5.77); S1PR5 (5.76); PRF1 (5.62); CTSW (5.61); XCL2 (5.52); XCL1 (5.33); FCRL6 (5.11); KRT86 (4.94); KLRC2 (4.92)	0.0	0.1	0.1	95.1	48.9	1.4	0.9	0.3	0.7	6.7	2.6	0.6	94.4	0.0	0.0
Sweat gland	735	3	DCD (10.89); HBA1 (9.59); SCGB2A2 (9.57); AZGP1 (9.51); HBA2 (9.49); MUCL1 (9.16); SCGB1B2P (9.00); S100A7 (8.95); KRT19 (8.77); AGR2 (8.51); KRT7 (8.29); ANKRD30A (8.23); PIP (8.23); CLDN4 (8.21); CYP4Z1 (7.92)	15.5	3.9	2.0	6.8	3.1	1.8	1.9	6.4	2.2	1.5	4.6	3.1	2.3	0.0	0.1

Class label	Cells	Libraries with ≥10 cells	Fifteen most specific genes (log2 fold-change versus the mean of the other classes)	KRT14	KRT5	KRT1	PTPRC	CD3E	CD79A	LYZ	CD14	CD68	PECAM1	COL1A1	DCD	NKG7	CD207	CD1A
T	11,440	8	IL17F (5.39); CD28 (4.89); CCR4 (4.86); RTKN2 (4.62); CTLA4 (4.54); IL17A (4.50); MAL (4.35); ICOS (4.28); CD40LG (4.28); TRAT1 (4.21); CAMK4 (4.19); IL2RA (4.06); CXCR6 (3.93); FOXP3 (3.88); SIRPG (3.82)	0.1	0.2	0.1	90.7	66.3	2.1	0.9	0.2	0.6	4.4	4.5	0.7	25.5	0.0	0.0

The gene columns give the percentage of the cells of the class in which the gene was detected (count above zero). In the class labelled keratinocyte (221 cells), PTPRC was detected in 84.6% of the cells and the most specific genes included plasmacytoid dendritic-cell genes (LILRA4, CLEC4C, PTCRA, PLD4); KRT5 was detected in 63.3% of its cells, but KRT14 and KRT1 in 0.5% and 0.0%. The class is therefore not keratinocyte-like, and the reference contains essentially no keratinocytes. CD207 and CD1A were detected almost exclusively in the monocyte/macrophage class (2.2% and 4.5% of its cells), which suggests that this class also contains Langerhans or other dendritic cells.

Table S14C. Validation of the composition estimators on pseudobulk mixtures of known composition

Main-text linkage: Methods 2.11; deconvolution Results.

Scheme	Method	Mixtures	Held-out libraries	Mono/Mac: Pearson r	Mono/Mac: RMSE (percentage points)	Mono/Mac: mean bias (percentage points)	Mono/Mac: r of per-library means	T cells: Pearson r	T cells: RMSE (percentage points)
A (leave one library out)	M1 (NNLS, 2000-gene signature)	160	8	0.58	4.2	2.4	0.59	0.18	25.2
A (leave one library out)	M2 (NNLS, signature without ER167 genes)	160	8	0.44	5.0	2.5	0.45	0.19	26.1
A (leave one library out)	M3 (NNLS, scaled marker genes)	160	8	0.92	4.0	3.0	0.92	0.87	14.2
A (leave one library out)	M4 (MuSiC)	160	8	0.80	1.6	−0.5	0.81	0.86	7.1
A (leave one library out)	M5 (BisqueRNA)	160	8	0.09	3.7	0.3	−0.41	−0.16	15.3
B (two folds of four libraries)	M1 (NNLS, 2000-gene signature)	160	8	0.50	4.1	1.3	0.51	0.33	29.7
B (two folds of four libraries)	M2 (NNLS, signature without ER167 genes)	160	8	0.47	3.6	0.7	0.46	0.33	29.8
B (two folds of four libraries)	M3 (NNLS, scaled marker genes)	160	8	0.91	3.6	2.3	0.92	0.87	13.4
B (two folds of four libraries)	M4 (MuSiC)	160	8	0.82	1.5	−0.5	0.82	0.83	8.1
B (two folds of four libraries)	M5 (BisqueRNA)	160	8	0.59	2.5	0.3	0.61	0.00	14.4

Scheme A: each library was left out in turn, the reference was built from the other seven libraries, and 20 bootstrap pseudobulk mixtures of the cells of the held-out library were deconvolved. Scheme B: the libraries were split into two folds of four, the reference was built from one fold, and the 80 bootstrap mixtures of the other fold were deconvolved together. BisqueRNA calibrates on between-sample variation in the bulk set, so scheme A is not informative for M5. The true monocyte/macrophage fraction of the mixtures ranged from 0.3% to 7.3%. Truth is the fraction of cells, whereas NNLS estimates fractions weighted by mRNA content, so the RMSE of M1-M3 includes a scale difference. M6 is a score and was not evaluated.

Table S14D. Estimated monocyte/macrophage content by method and tissue in the paired bulk cohorts

Main-text linkage: Methods 2.11; deconvolution Results; Figure 7a,b.

Cohort	Method	Tissue	n	Mean	Median	Interquartile range	Range	Samples with an estimate of exactly zero (paired rows: tied pairs)	Paired Wilcoxon P

Cohort	Method	Tissue	n	Mean	Median	Interquartile range	Range	Samples with an estimate of exactly zero (paired rows: tied pairs)	Paired Wilcoxon P
GSE249027	M1 (NNLS, 2000-gene signature)	non-lesional	15	0.00	0.00	0.00 to 0.00	0.00 to 0.00	15	–
GSE249027	M1 (NNLS, 2000-gene signature)	lesional	15	6.09	1.41	0.00 to 8.79	0.00 to 25.83	6	–
GSE249027	M1 (NNLS, 2000-gene signature)	lesional − non-lesional (paired)	15	6.09	1.41	–	–	6	0.00915
GSE249027	M2 (NNLS, signature without ER167 genes)	non-lesional	15	0.00	0.00	0.00 to 0.00	0.00 to 0.00	15	–
GSE249027	M2 (NNLS, signature without ER167 genes)	lesional	15	1.05	0.00	0.00 to 0.45	0.00 to 8.42	9	–
GSE249027	M2 (NNLS, signature without ER167 genes)	lesional − non-lesional (paired)	15	1.05	0.00	–	–	9	0.036
GSE249027	M3 (NNLS, scaled marker genes)	non-lesional	15	0.00	0.00	0.00 to 0.00	0.00 to 0.00	15	–
GSE249027	M3 (NNLS, scaled marker genes)	lesional	15	0.23	0.00	0.00 to 0.00	0.00 to 2.31	13	–
GSE249027	M3 (NNLS, scaled marker genes)	lesional − non-lesional (paired)	15	0.23	0.00	–	–	13	0.371
GSE249027	M4 (MuSiC)	non-lesional	15	3.67	3.41	2.91 to 4.14	1.61 to 6.87	0	–
GSE249027	M4 (MuSiC)	lesional	15	2.74	2.00	0.59 to 2.20	0.00 to 16.34	3	–
GSE249027	M4 (MuSiC)	lesional − non-lesional (paired)	15	−0.93	−1.83	–	–	0	0.0479
GSE249027	M5 (BisqueRNA)	non-lesional	15	4.59	4.38	4.09 to 5.37	2.98 to 5.66	0	–
GSE249027	M5 (BisqueRNA)	lesional	15	3.00	1.83	0.00 to 4.11	0.00 to 13.24	5	–
GSE249027	M5 (BisqueRNA)	lesional − non-lesional (paired)	15	−1.59	−2.23	–	–	0	0.0554
GSE249027	M6 (marker-gene score)	non-lesional	15	−0.67	−0.65	−0.75 to −0.52	−1.46 to −0.12	0	–
GSE249027	M6 (marker-gene score)	lesional	15	0.67	0.65	0.17 to 1.23	−0.84 to 2.03	0	–
GSE249027	M6 (marker-gene score)	lesional − non-lesional (paired)	15	1.34	1.31	–	–	0	8.54×10^{-4}
GSE151243	M1 (NNLS, 2000-gene signature)	perilesional	20	3.33	2.08	0.00 to 3.75	0.00 to 16.79	7	–
GSE151243	M1 (NNLS, 2000-gene signature)	lesional	20	13.93	10.74	6.55 to 22.04	0.00 to 32.11	2	–
GSE151243	M1 (NNLS, 2000-gene signature)	lesional − perilesional (paired)	20	10.60	9.59	–	–	1	9.00×10^{-4}
GSE151243	M2 (NNLS, signature without ER167 genes)	perilesional	20	1.71	0.00	0.00 to 2.37	0.00 to 9.11	11	–
GSE151243	M2 (NNLS, signature without ER167 genes)	lesional	20	5.94	4.91	0.00 to 12.14	0.00 to 16.67	7	–
GSE151243	M2 (NNLS, signature without ER167 genes)	lesional − perilesional (paired)	20	4.23	3.14	–	–	6	0.00574
GSE151243	M3 (NNLS, scaled marker genes)	perilesional	20	1.68	1.80	1.19 to 2.12	0.00 to 4.30	3	–
GSE151243	M3 (NNLS, scaled marker genes)	lesional	20	4.56	3.72	2.10 to 7.03	0.00 to 14.51	2	–
GSE151243	M3 (NNLS, scaled marker genes)	lesional − perilesional (paired)	20	2.88	2.67	–	–	0	0.0121
GSE151243	M4 (MuSiC)	perilesional	20	6.50	6.49	3.92 to 7.75	2.73 to 14.35	0	–
GSE151243	M4 (MuSiC)	lesional	20	5.86	5.20	3.15 to 8.66	0.00 to 12.12	1	–

Cohort	Method	Tissue	n	Mean	Median	Interquartile range	Range	Samples with an estimate of exactly zero (paired rows: tied pairs)	Paired Wilcoxon P
GSE151243	M4 (MuSiC)	lesional − perilesional (paired)	20	−0.64	−0.61	–	–	0	0.452
GSE151243	M5 (BisqueRNA)	perilesional	20	4.44	4.54	3.32 to 5.00	0.94 to 9.27	0	–
GSE151243	M5 (BisqueRNA)	lesional	20	2.52	1.95	1.03 to 3.86	0.00 to 6.67	4	–
GSE151243	M5 (BisqueRNA)	lesional − perilesional (paired)	20	−1.92	−2.10	–	–	0	0.00558
GSE151243	M6 (marker-gene score)	perilesional	20	−0.68	−0.74	−0.88 to −0.41	−1.61 to 0.30	0	–
GSE151243	M6 (marker-gene score)	lesional	20	0.68	0.72	0.30 to 1.11	−0.78 to 1.71	0	–
GSE151243	M6 (marker-gene score)	lesional − perilesional (paired)	20	1.37	1.52	–	–	0	1.91×10^{-6}

M1-M5: estimated fraction in percent; M6: mean z-standardised marker score. In the paired rows, mean and median refer to the within-patient difference between lesional and comparator tissue. The Wilcoxon signed-rank test was exact when there were no ties or zero differences and otherwise used the normal approximation with continuity correction.

Table S14E. Spearman correlation between the bulk ER167 score and the estimated monocyte/macrophage content

Main-text linkage: Methods 2.11; deconvolution Results.

Cohort	Method	All samples (n)	ρ, all samples	P	Lesional samples (n)	ρ, lesional samples	P
GSE249027	M1 (NNLS, 2000-gene signature)	30	0.65	9.07×10^{-5}	15	0.34	0.216
GSE249027	M2 (NNLS, signature without ER167 genes)	30	0.60	4.06×10^{-4}	15	0.62	0.0136
GSE249027	M3 (NNLS, scaled marker genes)	30	0.42	0.0224	15	0.54	0.0389
GSE249027	M4 (MuSiC)	30	−0.29	0.118	15	0.20	0.465
GSE249027	M5 (BisqueRNA)	30	−0.33	0.0794	15	0.05	0.847
GSE249027	M6 (marker-gene score)	30	0.92	6.67×10^{-13}	15	0.89	7.49×10^{-6}
GSE151243	M1 (NNLS, 2000-gene signature)	40	0.61	3.10×10^{-5}	20	0.59	0.00629
GSE151243	M2 (NNLS, signature without ER167 genes)	40	0.47	0.00206	20	0.53	0.0155
GSE151243	M3 (NNLS, scaled marker genes)	40	0.40	0.0102	20	0.47	0.0358
GSE151243	M4 (MuSiC)	40	0.23	0.149	20	0.63	0.00318
GSE151243	M5 (BisqueRNA)	40	−0.16	0.327	20	0.44	0.0513
GSE151243	M6 (marker-gene score)	40	0.95	1.39×10^{-21}	20	0.86	1.16×10^{-6}

Table S14F. Attenuation of the ER167 disease statistic after composition adjustment, by method

Main-text linkage: Methods 2.11; deconvolution Results; Figure 7c,d; Table S10B.

Cohort	Method	Covariates added to the paired model	ER167 genes	Unadjusted mean t^2	Adjusted mean t^2	Attenuation (%)	Unadjusted Fry mixed P (order-invariant)	Adjusted Fry mixed P (order-invariant)	Adjusted Fry directional P (direction)	Adjusted Fry mixed P (weighted test, original order)
GSE249027	M1 (NNLS, 2000-gene signature)	Monocyte/macrophage estimate	162	32.84	19.91	39.4	8.56×10^{-8}	1.22×10^{-5}	2.33×10^{-4} (up)	1.72×10^{-6}
GSE249027	M1 (NNLS, 2000-gene signature)	All immune classes: Mono/Mac, T, B/plasma (NK constant, dropped)	162	32.84	6.57	80.0	8.56×10^{-8}	0.0128	0.026 (up)	0.0023
GSE249027	M2 (NNLS, signature without ER167 genes)	Monocyte/macrophage estimate	162	32.84	24.09	26.7	8.56×10^{-8}	5.88×10^{-7}	6.63×10^{-5} (up)	6.42×10^{-8}
GSE249027	M2 (NNLS, signature without ER167 genes)	All immune classes: Mono/Mac, T, B/plasma (NK constant, dropped)	162	32.84	7.44	77.4	8.56×10^{-8}	0.00375	0.0188 (up)	2.21×10^{-4}
GSE249027	M3 (NNLS, scaled marker genes)	Monocyte/macrophage estimate	162	32.84	26.46	19.4	8.56×10^{-8}	4.35×10^{-7}	4.47×10^{-5} (up)	2.98×10^{-8}
GSE249027	M3 (NNLS, scaled marker genes)	All immune classes: Mono/Mac, T, B/plasma (NK constant, dropped)	162	32.84	11.41	65.3	8.56×10^{-8}	1.65×10^{-4}	0.00411 (up)	6.81×10^{-6}
GSE249027	M4 (MuSiC)	Monocyte/macrophage estimate	162	32.84	32.42	1.3	8.56×10^{-8}	2.08×10^{-7}	1.79×10^{-5} (up)	2.66×10^{-8}
GSE249027	M4 (MuSiC)	All immune classes: Mono/Mac, T, B/plasma, NK	162	32.84	7.48	77.2	8.56×10^{-8}	0.00494	0.0161 (up)	0.00109
GSE249027	M5 (BisqueRNA)	Monocyte/macrophage estimate	162	32.84	29.68	9.6	8.56×10^{-8}	5.59×10^{-7}	3.40×10^{-5} (up)	5.33×10^{-8}
GSE249027	M5 (BisqueRNA)	All immune classes: Mono/Mac, T, B/plasma, NK	162	32.84	6.03	81.6	8.56×10^{-8}	0.00496	0.02 (up)	4.29×10^{-4}
GSE249027	M6 (marker-gene score)	Monocyte/macrophage estimate	162	32.84	3.96	87.9	8.56×10^{-8}	3.94×10^{-4}	0.0414 (up)	5.30×10^{-5}
GSE151243	M1 (NNLS, 2000-gene signature)	Monocyte/macrophage estimate	164	43.77	13.66	68.8	9.75×10^{-11}	5.78×10^{-9}	3.61×10^{-5} (up)	1.05×10^{-10}
GSE151243	M1 (NNLS, 2000-gene signature)	All immune classes: Mono/Mac, T, B/plasma (NK constant, dropped)	164	43.77	15.80	63.9	9.75×10^{-11}	3.04×10^{-9}	5.09×10^{-6} (up)	3.59×10^{-10}
GSE151243	M2 (NNLS, signature without ER167 genes)	Monocyte/macrophage estimate	164	43.77	23.05	47.3	9.75×10^{-11}	4.85×10^{-11}	1.42×10^{-7} (up)	1.11×10^{-12}
GSE151243	M2 (NNLS, signature without ER167 genes)	All immune classes: Mono/Mac, T, B/plasma (NK constant, dropped)	164	43.77	16.46	62.4	9.75×10^{-11}	1.25×10^{-8}	2.57×10^{-6} (up)	7.36×10^{-11}
GSE151243	M3 (NNLS, scaled marker genes)	Monocyte/macrophage estimate	164	43.77	26.19	40.2	9.75×10^{-11}	1.15×10^{-9}	2.22×10^{-7} (up)	1.24×10^{-13}
GSE151243	M3 (NNLS, scaled marker genes)	All immune classes: Mono/Mac, T, B/plasma, NK	164	43.77	7.89	82.0	9.75×10^{-11}	1.01×10^{-5}	7.10×10^{-4} (up)	1.98×10^{-11}
GSE151243	M4 (MuSiC)	Monocyte/macrophage estimate	164	43.77	55.09	−25.9	9.75×10^{-11}	3.19×10^{-12}	8.81×10^{-10} (up)	2.07×10^{-14}
GSE151243	M4 (MuSiC)	All immune classes: Mono/Mac, T, B/plasma (NK constant, dropped)	164	43.77	16.19	63.0	9.75×10^{-11}	2.03×10^{-9}	8.33×10^{-6} (up)	1.03×10^{-9}
GSE151243	M5 (BisqueRNA)	Monocyte/macrophage estimate	164	43.77	38.49	12.1	9.75×10^{-11}	2.59×10^{-10}	3.68×10^{-8} (up)	4.03×10^{-14}
GSE151243	M5 (BisqueRNA)	All immune classes: Mono/Mac, T, B/plasma, NK	164	43.77	13.28	69.7	9.75×10^{-11}	7.75×10^{-8}	6.11×10^{-5} (up)	5.29×10^{-9}

Cohort	Method	Covariates added to the paired model	ER167 genes	Unadjusted mean t²	Adjusted mean t²	Attenuation (%)	Unadjusted Fry mixed P (order-invariant)	Adjusted Fry mixed P (order-invariant)	Adjusted Fry directional P (direction)	Adjusted Fry mixed P (weighted test, original order)
GSE151243	M6 (marker-gene score)	Monocyte/macrophage estimate	164	43.77	2.50	94.3	9.75×10^{-11}	0.0376	0.0277 (up)	5.20×10^{-5}

Attenuation is the proportional reduction in the mean squared moderated t statistic of ER167; a negative value means that the statistic increased after adjustment. Classes whose estimate was constant across the samples of a cohort were dropped from the model.

Table S14G. Agreement between the six estimates of monocyte/macrophage content (Spearman correlation across samples)

Main-text linkage: Methods 2.11; deconvolution Results.

Cohort	Method	M1	M2	M3	M4	M5	M6
GSE249027	M1 (NNLS, 2000-gene signature)	1.00	0.79	0.46	−0.08	−0.17	0.66
GSE249027	M2 (NNLS, signature without ER167 genes)	0.79	1.00	0.60	−0.01	−0.06	0.66
GSE249027	M3 (NNLS, scaled marker genes)	0.46	0.60	1.00	0.39	0.43	0.43
GSE249027	M4 (MuSiC)	−0.08	−0.01	0.39	1.00	0.87	−0.17
GSE249027	M5 (BisqueRNA)	−0.17	−0.06	0.43	0.87	1.00	−0.20
GSE249027	M6 (marker-gene score)	0.66	0.66	0.43	−0.17	−0.20	1.00
GSE151243	M1 (NNLS, 2000-gene signature)	1.00	0.87	0.48	0.34	−0.05	0.67
GSE151243	M2 (NNLS, signature without ER167 genes)	0.87	1.00	0.49	0.42	0.07	0.51
GSE151243	M3 (NNLS, scaled marker genes)	0.48	0.49	1.00	0.43	0.20	0.53
GSE151243	M4 (MuSiC)	0.34	0.42	0.43	1.00	0.82	0.25
GSE151243	M5 (BisqueRNA)	−0.05	0.07	0.20	0.82	1.00	−0.13
GSE151243	M6 (marker-gene score)	0.67	0.51	0.53	0.25	−0.13	1.00

Table S14H. Diagnostics of the non-negative least squares fit with the 2000-gene signature (method M1)

Main-text linkage: Methods 2.11; deconvolution Results (Section 3.9).

Cohort	Tissue	Samples	ER167 genes among the 2000 signature genes	Share of the sum of squares carried by the 20 most highly expressed signature genes (%)	Relative residual of the fit, mean (range)	Correlation of observed with fitted expression, linear scale (mean)	Correlation of observed with fitted expression, log2 scale (mean)	Estimated monocyte/macrophage fraction, mean (%)	Samples with a monocyte/macrophage estimate of exactly zero
GSE249027	Lesional	15	80	95.9	0.81 (0.61 to 1.00)	0.53	0.51	6.1	6 of 15
GSE249027	Non-lesional	15	80	95.9	0.98 (0.94 to 1.00)	0.15	0.44	0.0	15 of 15
GSE151243	Lesional	20	80	91.2	0.76 (0.58 to 0.92)	0.63	0.53	13.9	2 of 20
GSE151243	Perilesional	20	80	91.2	0.93 (0.77 to 0.99)	0.31	0.47	3.3	7 of 20

Each bulk sample was fitted by non-negative least squares to the eight class profiles of the signature on the linear counts-per-million scale. Relative residual: Euclidean norm of the difference between observed and fitted expression divided by the norm of the observed expression; a value of 1 means that the fit explains none of the sum of squares. The share of the sum of squares is that of the 20 signature genes with the highest mean expression in the cohort, across all samples of the cohort. Values per cohort (signature genes, share of the sum of squares) are repeated in both rows of the cohort.

Table S15A. Donor-level metadata available in GEO for the discovery and replication single-cell cohorts

Main-text linkage: Methods 2.8; donor-level Results; Limitations.

Cohort	Donor	GEO sample	Group	Age (years)	Sex	Tissue (GEO field)	Hurley stage or severity	Current treatment	Library or sequencing batch	Instrument	Mono/Mac cells	Pseudobulk library size
GSE249793	HS10x2	GSM7964413	HS	not reported	not reported	skin from HS lesion	not reported	not reported	not reported	Illumina NovaSeq 6000	397	2,026,107
GSE249793	HS10x4	GSM7964412	HS	not reported	not reported	skin from HS lesion	not reported	not reported	not reported	Illumina NovaSeq 6000	220	852,641
GSE249793	HS10x5	GSM7964410	HS	not reported	not reported	skin from HS lesion	not reported	not reported	not reported	Illumina NovaSeq 6000	967	2,825,240
GSE249793	HS10x6	GSM7964411	HS	not reported	not reported	skin from HS lesion	not reported	not reported	not reported	Illumina NovaSeq 6000	133	398,408
GSE249793	NST10	GSM7964424	HC	not reported	not reported	skin from healthy donor	not reported	not reported	not reported	Illumina NovaSeq 6000	3,066	28,109,901
GSE249793	NST5	GSM7964421	HC	not reported	not reported	skin from healthy donor	not reported	not reported	not reported	Illumina NovaSeq 6000	100	645,766
GSE249793	NST7	GSM7964420	HC	not reported	not reported	skin from healthy donor	not reported	not reported	not reported	Illumina NovaSeq 6000	490	1,787,945
GSE249793	NST8	GSM7964422	HC	not reported	not reported	skin from healthy donor	not reported	not reported	not reported	Illumina NovaSeq 6000	3,118	23,784,192
GSE249793	NST9	GSM7964423	HC	not reported	not reported	skin from healthy donor	not reported	not reported	not reported	Illumina NovaSeq 6000	1,137	8,209,445
GSE212721	HS1	GSM6544080	HS	22	not reported	excised surgical tissue from seton intervention	not reported	not reported	not reported	NextSeq 500	–	–
GSE212721	HS2	GSM6544081	HS	43	not reported	excised surgical tissue from seton intervention	not reported	not reported	not reported	NextSeq 500	–	–
GSE212721	HS3	GSM6544082	HS	62	not reported	excised surgical tissue from seton intervention	not reported	not reported	not reported	NextSeq 500	–	–
GSE212721	HS4	GSM6544083	HS	31	not reported	excised surgical tissue from seton intervention	not reported	not reported	not reported	NextSeq 500	–	–
GSE212721	Con1	GSM6544084	HC	58	not reported	Skin from mammoplasty surgery	not reported	not reported	not reported	NextSeq 500	–	–
GSE212721	Con2	GSM6544085	HC	41	not reported	Skin from mammoplasty surgery	not reported	not reported	not reported	NextSeq 500	–	–
GSE212721	Con3	GSM6544086	HC	24	not reported	Skin from mammoplasty surgery	not reported	not reported	not reported	NextSeq 500	–	–
GSE212721	HS5	GSM6544087	HS	23	not reported	excised surgical tissue from seton intervention	not reported	not reported	not reported	NextSeq 500	–	–
GSE212721	HS6	GSM6544088	HS	51	not reported	excised surgical tissue from seton intervention	not reported	not reported	not reported	NextSeq 500	–	–

Fields that are not given in the GEO sample records are shown as not reported and were not inferred. Cell numbers and library sizes refer to the discovery monocyte/macrophage pseudobulk profiles of the nine donors of the primary analysis; they were not available for GSE212721, for which the pseudobulk object was not archived. Discovery donor HS419, which contributed 14 monocyte/macrophage cells and failed the ≥30-cell gate, is not listed.

Table S15B. Leave-one-donor-out analysis of the primary ER167 endpoint
Main-text linkage: Methods 2.8; donor-level Results.

Donor removed	Group	HS donors	HC donors	Genes tested	ER167 genes detected	ER167 genes at FDR<0.05	Fry mixed P (order-invariant)	Fry directional P (order-invariant)	Fry mixed P (weighted test, original order)	Matched-null empirical P	Pearson r of ER167 log2 fold-changes with the full analysis	Sign concordance with the full analysis (%)
None (all nine donors)	–	4	5	7,155	141	62 (36 up, 26 down)	8.56×10^{-7}	0.479	5.96×10^{-9}	1/10,001	1.000	100.0
HS10x2	HS	3	5	7,853	148	49 (34 up, 15 down)	6.87×10^{-6}	0.173	1.13×10^{-7}	1/10,001	0.993	95.0
HS10x4	HS	3	5	8,004	147	65 (35 up, 30 down)	6.82×10^{-6}	0.946	3.37×10^{-7}	1/10,001	0.969	95.7
HS10x5	HS	3	5	7,100	143	49 (33 up, 16 down)	9.24×10^{-7}	0.508	4.87×10^{-8}	1/10,001	0.944	97.1
HS10x6	HS	3	5	7,926	147	55 (33 up, 22 down)	2.04×10^{-5}	0.643	3.28×10^{-8}	1/10,001	0.992	95.7
NST10	HC	4	4	6,828	136	51 (34 up, 17 down)	1.26×10^{-6}	0.133	1.26×10^{-7}	1/10,001	0.992	94.1
NST5	HC	4	4	7,316	143	71 (38 up, 33 down)	2.97×10^{-6}	0.915	1.16×10^{-7}	1/10,001	0.988	97.2
NST7	HC	4	4	7,193	139	65 (40 up, 25 down)	1.19×10^{-5}	0.383	5.60×10^{-7}	1/10,001	0.991	93.5
NST8	HC	4	4	6,795	137	48 (32 up, 16 down)	1.46×10^{-6}	0.232	6.11×10^{-8}	1/10,001	0.997	93.4
NST9	HC	4	4	6,769	136	58 (36 up, 22 down)	1.15×10^{-5}	0.435	1.11×10^{-7}	1/10,001	0.995	96.3

Each analysis was repeated from the raw pseudobulk counts (filtering, normalization, differential expression, Fry and 10,000-set matched null) after removal of one donor.

Table S15C. Association of the first four principal components with group, library size and cell number
Main-text linkage: Methods 2.8; donor-level Results; Figure S8c.

Principal component	Variance explained (%)	HS vs HC: exact Wilcoxon P	Library size: Spearman ρ	P	Cell number: Spearman ρ	P
PC1	38.1	0.0159	0.57	0.121	0.58	0.108
PC2	16.0	0.413	−0.10	0.81	−0.15	0.708
PC3	13.5	1	0.72	0.0369	0.73	0.0311
PC4	10.8	1	0.17	0.678	−0.13	0.744

Principal component analysis of the log2 counts per million of the 7155 tested genes (centred and scaled) in the nine donors. P values are nominal (four components and three tests; descriptive). With four and five donors, the smallest attainable two-sided exact Wilcoxon P value is 0.0159.

Table S15D. Covariates available for the discovery cohort and covariate-adjusted ER167 result
Main-text linkage: Methods 2.8; donor-level Results; Limitations.

Covariate	Availability and handling	Residual degrees of freedom	Fry mixed P with the covariate (order-invariant)	Fry mixed P with the covariate (weighted test, original order)
Age	Not reported in GEO; cannot be assessed	–	–	–
Sex	Not reported in GEO; cannot be assessed	–	–	–

Covariate	Availability and handling	Residual degrees of freedom	Fry mixed P with the covariate (order-invariant)	Fry mixed P with the covariate (weighted test, original order)
Tissue (GEO field)	Reported; completely aliased with group (HS lesion versus healthy skin); effect of group not estimable after adjustment	–	–	–
Hurley stage or severity	Not reported in GEO; cannot be assessed	–	–	–
Current treatment	Not reported in GEO; cannot be assessed	–	–	–
Library or sequencing batch	Not reported in GEO; cannot be assessed	–	–	–
Processing date	Not reported in GEO; cannot be assessed	–	–	–
GEO submission date	Reported; identical in all nine donors	–	–	–
Sequencing instrument	Reported; identical in all nine donors	–	–	–
Number of mono/mac cells	Computed in this study; added to the model as a linear covariate on the original scale	6	2.34×10^{-6}	7.72×10^{-7}
Pseudobulk library size	Computed in this study; added to the model as a linear covariate on the original scale	6	8.54×10^{-7}	6.59×10^{-7}

With nine donors, the covariate models are sensitivity checks only. Library size and cell number are technical variables computed from the data, not GEO fields.

Table S15E. Tagwise negative-binomial dispersion in the discovery pseudobulk analysis
Main-text linkage: Methods 2.8; donor-level Results.

Genes	n	Minimum	25th percentile	Median	75th percentile	Maximum	Biological coefficient of variation at the median
all 7155	7,155	0.053	0.096	0.131	0.194	3.362	0.363
ER167 (141)	141	0.067	0.149	0.207	0.335	2.688	0.455
non-ER167 (7014)	7,014	0.053	0.096	0.130	0.191	3.362	0.361

edgeR dispersion estimates of the nine-donor analysis. Common dispersion 0.173 (biological coefficient of variation 0.416); median trended dispersion 0.163 (0.403). The tagwise dispersion of the ER167 genes was higher than that of the other genes (Wilcoxon rank-sum $P=3.5\times10^{-21}$).

Table S15F. Leave-one-out projection of the ER167 profile of each discovery donor
Main-text linkage: Methods 2.8; donor-level Results.

Donor	Group	ER167 genes compared	Control donors in the reference	Pearson r between the donor's ER167 deviation and the log2 fold-changes estimated without that donor
HS10x2	HS	141	5	0.885
HS10x4	HS	141	5	0.744
HS10x5	HS	140	5	0.772
HS10x6	HS	141	5	0.827
NST10	HC	136	4	−0.004
NST5	HC	141	4	0.432

Donor	Group	ER167 genes compared	Control donors in the reference	Pearson r between the donor's ER167 deviation and the log2 fold-changes estimated without that donor
NST7	HC	138	4	0.019
NST8	HC	137	4	−0.234
NST9	HC	136	4	0.130

For each donor, the deviation is its log2 counts per million minus the mean of the control donors among the other eight donors, for each detected ER167 gene; the log2 fold-changes (HS versus control) were estimated with the full pipeline from the other eight donors. A high positive correlation means that the donor shows the disease-associated ER167 pattern estimated independently of it. P values are not given because the genes are not independent.

Table S16A. Monocyte/macrophage cells per donor and state in the discovery cohort

Main-text linkage: Methods 2.9; within-state Results; Table S7.

Donor	Group	Mono_FCN1_CCL3	Mono_FCN1_infl	MonoMac_hypoxic_lipid	MonoMac_ribo_hi	Mac_MMP9_SPP1	Mac_HLADR_APC	Mac_C1QC_MRC1	All mono/mac cells	In the primary analysis
HS10x2	HS	78	240	56	5	15	2	1	397	yes
HS10x4	HS	58	54	66	6	13	5	18	220	yes
HS10x5	HS	219	652	66	0	27	0	3	967	yes
HS10x6	HS	47	33	32	0	15	0	6	133	yes
HS419	HS	4	0	1	0	0	0	9	14	no
NST10	HC	0	12	619	765	758	618	294	3,066	yes
NST5	HC	1	10	29	4	39	1	16	100	yes
NST7	HC	4	16	74	6	73	27	290	490	yes
NST8	HC	1	10	585	880	425	579	638	3,118	yes
NST9	HC	0	24	228	21	580	32	252	1,137	yes

HS419 contributed 14 monocyte/macrophage cells and was excluded from the primary donor-level analysis by the ≥30-cell gate. NST8 and NST10 together contributed 6184 of the 9628 cells of the nine donors of the primary analysis (64%).

Table S16B. Within-state ER167 tests by minimum number of cells per donor

Main-text linkage: Methods 2.9; within-state Results; Figure S11; Table S7B.

Minimum cells per donor	State	Testable	HS donors	HC donors	Smallest number of cells in a retained donor	Genes tested	ER167 genes detected	Fry mixed P (order-invariant)	Fry directional P (direction)	Standardised order-invariant mixed P	Fry mixed P (weighted test, original order)	Matched-null empirical P	Fry P, BH across testable states	Fry P, Holm	Empirical P, BH across testable states	Empirical P, Holm
≥10	Mono_FCN1_CCL3	no	4	0	–	–	–	–	–	–	–	–	–	–	–	–
≥10	Mono_FCN1_infl	yes	4	5	10	925	52	2.99×10^{-6}	0.0135 (up)	3.04×10^{-8}	1.16×10^{-5}	5/10,001	2.99×10^{-6}	4.02×10^{-6}	0.0015	0.0015
≥10	MonoMac_hypoxic_lipid	yes	4	5	29	2,595	86	7.93×10^{-8}	0.568 (down)	1.71×10^{-9}	1.90×10^{-9}	0.0022	2.38×10^{-7}	2.38×10^{-7}	0.003	0.0044

Minimum cells per donor	State	Testable	HS donors	HC donors	Smallest number of cells in a retained donor	Genes tested	ER167 genes detected	Fry mixed P (order-invariant)	Fry directional P (direction)	Standardised order-invariant mixed P	Fry mixed P (weighted test, original order)	Matched-null empirical P	Fry P, BH across testable states	Fry P, Holm	Empirical P, BH across testable states	Empirical P, Holm
≥10	MonoMac_ribo_hi	no	0	3	–	–	–	–	–	–	–	–	–	–	–	–
≥10	Mac_MMP9_SPP1	yes	4	5	13	2,360	61	2.01×10^{-6}	0.0316 (down)	1.04×10^{-7}	5.61×10^{-8}	0.003	2.99×10^{-6}	4.02×10^{-6}	0.003	0.0044
≥10	Mac_HLADR_APC	no	0	4	–	–	–	–	–	–	–	–	–	–	–	–
≥10	Mac_C1QC_MRC1	no	1	5	–	–	–	–	–	–	–	–	–	–	–	–
≥20	Mono_FCN1_CCL3	no	4	0	–	–	–	–	–	–	–	–	–	–	–	–
≥20	Mono_FCN1_infl	no	4	1	–	–	–	–	–	–	–	–	–	–	–	–
≥20	MonoMac_hypoxic_lipid	yes	4	5	29	2,595	86	7.93×10^{-8}	0.568 (down)	1.71×10^{-9}	1.90×10^{-9}	0.0022	7.93×10^{-8}	7.93×10^{-8}	0.0022	0.0022
≥20	MonoMac_ribo_hi	no	0	3	–	–	–	–	–	–	–	–	–	–	–	–
≥20	Mac_MMP9_SPP1	no	1	5	–	–	–	–	–	–	–	–	–	–	–	–
≥20	Mac_HLADR_APC	no	0	4	–	–	–	–	–	–	–	–	–	–	–	–
≥20	Mac_C1QC_MRC1	no	0	4	–	–	–	–	–	–	–	–	–	–	–	–
≥30	Mono_FCN1_CCL3	no	4	0	–	–	–	–	–	–	–	–	–	–	–	–
≥30	Mono_FCN1_infl	no	4	0	–	–	–	–	–	–	–	–	–	–	–	–
≥30	MonoMac_hypoxic_lipid	yes	4	4	32	2,562	84	2.95×10^{-6}	0.395 (down)	1.91×10^{-7}	1.49×10^{-7}	0.0007	2.95×10^{-6}	2.95×10^{-6}	7.00×10^{-4}	7.00×10^{-4}
≥30	MonoMac_ribo_hi	no	0	2	–	–	–	–	–	–	–	–	–	–	–	–
≥30	Mac_MMP9_SPP1	no	0	5	–	–	–	–	–	–	–	–	–	–	–	–
≥30	Mac_HLADR_APC	no	0	3	–	–	–	–	–	–	–	–	–	–	–	–
≥30	Mac_C1QC_MRC1	no	0	4	–	–	–	–	–	–	–	–	–	–	–	–
≥50	Mono_FCN1_CCL3	no	3	0	–	–	–	–	–	–	–	–	–	–	–	–
≥50	Mono_FCN1_infl	no	3	0	–	–	–	–	–	–	–	–	–	–	–	–
≥50	MonoMac_hypoxic_lipid	yes	3	4	56	2,936	98	7.83×10^{-6}	0.322 (down)	5.07×10^{-7}	2.25×10^{-7}	0.0034	7.83×10^{-6}	7.83×10^{-6}	0.0034	0.0034
≥50	MonoMac_ribo_hi	no	0	2	–	–	–	–	–	–	–	–	–	–	–	–
≥50	Mac_MMP9_SPP1	no	0	4	–	–	–	–	–	–	–	–	–	–	–	–
≥50	Mac_HLADR_APC	no	0	2	–	–	–	–	–	–	–	–	–	–	–	–
≥50	Mac_C1QC_MRC1	no	0	4	–	–	–	–	–	–	–	–	–	–	–	–

A state was testable at a threshold when at least three donors in each group contributed at least that number of cells of the state; donor numbers are those meeting the threshold. Adjustment for multiplicity was applied across the states testable at the same threshold.

Table S16C. Leave-one-donor-out within-state ER167 tests (minimum of 10 cells per donor)

Main-text linkage: Methods 2.9; within-state Results.

Donor removed	State	HS donors	HC donors	Smallest number of cells in a retained donor	Genes tested	ER167 genes detected	Fry mixed P (order-invariant)	Fry directional P (direction)	Standardised order-invariant mixed P	Fry mixed P (weighted test, original order)
HS10x2	Mac_MMP9_SPP1	3	5	13	2,782	74	5.23×10^{-6}	0.0866 (down)	2.62×10^{-7}	5.80×10^{-6}
HS10x4	Mac_MMP9_SPP1	3	5	15	2,769	76	2.43×10^{-6}	0.149 (down)	2.29×10^{-7}	1.87×10^{-8}
HS10x5	Mac_MMP9_SPP1	3	5	13	2,746	74	2.03×10^{-5}	0.0472 (down)	1.91×10^{-6}	2.77×10^{-5}
HS10x6	Mac_MMP9_SPP1	3	5	13	2,715	71	1.15×10^{-6}	0.0144 (down)	5.67×10^{-8}	3.54×10^{-7}
NST10	Mac_MMP9_SPP1	4	4	13	1,974	57	1.42×10^{-5}	0.059 (down)	1.18×10^{-6}	1.16×10^{-6}
NST5	Mac_MMP9_SPP1	4	4	13	2,083	57	3.33×10^{-5}	0.0113 (down)	4.50×10^{-6}	3.15×10^{-6}
NST7	Mac_MMP9_SPP1	4	4	13	1,870	56	1.63×10^{-5}	0.0586 (down)	1.56×10^{-6}	5.22×10^{-7}
NST8	Mac_MMP9_SPP1	4	4	13	1,942	56	1.73×10^{-5}	0.0541 (down)	1.49×10^{-6}	1.10×10^{-6}
NST9	Mac_MMP9_SPP1	4	4	13	1,944	55	2.47×10^{-5}	0.0664 (down)	1.57×10^{-7}	3.74×10^{-7}
HS10x2	MonoMac_hypoxic_lipid	3	5	29	2,709	90	1.80×10^{-5}	0.606 (down)	2.89×10^{-12}	1.51×10^{-6}
HS10x4	MonoMac_hypoxic_lipid	3	5	29	2,839	88	1.33×10^{-5}	0.26 (down)	8.70×10^{-8}	6.99×10^{-7}
HS10x5	MonoMac_hypoxic_lipid	3	5	29	2,901	94	9.95×10^{-6}	0.738 (down)	7.04×10^{-8}	1.08×10^{-7}
HS10x6	MonoMac_hypoxic_lipid	3	5	29	3,008	96	1.26×10^{-6}	0.421 (down)	5.37×10^{-9}	4.20×10^{-9}
NST10	MonoMac_hypoxic_lipid	4	4	29	2,482	81	1.58×10^{-6}	0.705 (up)	2.90×10^{-7}	8.92×10^{-9}
NST5	MonoMac_hypoxic_lipid	4	4	32	2,562	84	2.95×10^{-6}	0.395 (down)	1.91×10^{-7}	1.49×10^{-7}
NST7	MonoMac_hypoxic_lipid	4	4	29	2,529	85	1.23×10^{-5}	0.944 (down)	1.69×10^{-7}	1.27×10^{-6}
NST8	MonoMac_hypoxic_lipid	4	4	29	2,467	81	1.85×10^{-6}	0.661 (down)	1.92×10^{-7}	2.70×10^{-8}
NST9	MonoMac_hypoxic_lipid	4	4	29	2,431	81	3.57×10^{-6}	0.699 (down)	1.78×10^{-8}	2.89×10^{-9}
HS10x2	Mono_FCN1_infl	3	5	10	914	55	0.26	0.091 (up)	1.67×10^{-5}	3.73×10^{-6}
HS10x4	Mono_FCN1_infl	3	5	10	888	52	0.122	0.565 (up)	9.34×10^{-6}	5.72×10^{-5}
HS10x5	Mono_FCN1_infl	3	5	10	908	54	0.00224	0.256 (up)	5.07×10^{-6}	2.84×10^{-6}
HS10x6	Mono_FCN1_infl	3	5	10	1,245	61	0.00426	0.202 (up)	1.33×10^{-6}	6.03×10^{-5}
NST10	Mono_FCN1_infl	4	4	10	1,176	55	1.17×10^{-5}	0.00247 (up)	4.23×10^{-7}	1.39×10^{-5}
NST5	Mono_FCN1_infl	4	4	10	1,125	54	1.07×10^{-4}	0.0135 (up)	9.62×10^{-7}	1.32×10^{-4}
NST7	Mono_FCN1_infl	4	4	10	1,115	52	8.50×10^{-5}	0.0287 (up)	7.49×10^{-8}	1.13×10^{-5}
NST8	Mono_FCN1_infl	4	4	10	1,129	57	2.23×10^{-5}	0.0201 (up)	1.54×10^{-7}	6.10×10^{-4}
NST9	Mono_FCN1_infl	4	4	10	769	45	0.00174	0.0842 (up)	1.43×10^{-6}	5.69×10^{-5}

Summary: Mac_MMP9_SPP1, order-invariant P from 1.15×10^{-6} to 3.33×10^{-5} (0 of 9 analyses with $P\geq0.05$); MonoMac_hypoxic_lipid, order-invariant P from 1.26×10^{-6} to 1.80×10^{-5} (0 of 9 analyses with $P\geq0.05$); Mono_FCN1_infl, order-invariant P from 1.17×10^{-5} to 0.26 (2 of 9 analyses with $P\geq0.05$). The matched null was not repeated in the leave-one-out analyses.

Table S17A. Simulated rejection rate of the mixed-direction Fry test for a gene set of the size of ER167
Main-text linkage: Methods 2.8; power Results; Figure S12b,c.

Design	Quantity	Genes of the set affected (%)	True absolute log2 fold-change	Replicates	Median number of set genes after filtering	Rejection rate at P<0.05, order-invariant test	Monte Carlo standard error	Rejection rate at P<0.05, weighted test
4 HS vs 5 HC (discovery; ER167, 141 genes)	type I error	–	0.0	200	135	0.075	0.019	0.125
4 HS vs 5 HC (discovery; ER167, 141 genes)	power	10	0.5	200	135	0.080	0.019	0.210
4 HS vs 5 HC (discovery; ER167, 141 genes)	power	10	1.0	200	135	0.280	0.032	0.790
4 HS vs 5 HC (discovery; ER167, 141 genes)	power	10	1.5	200	135	0.795	0.029	0.995
4 HS vs 5 HC (discovery; ER167, 141 genes)	power	10	2.0	200	135	0.975	0.011	1.000
4 HS vs 5 HC (discovery; ER167, 141 genes)	power	25	0.5	200	135	0.150	0.025	0.620
4 HS vs 5 HC (discovery; ER167, 141 genes)	power	25	1.0	200	135	0.785	0.029	1.000
4 HS vs 5 HC (discovery; ER167, 141 genes)	power	25	1.5	200	135	1.000	0.000	1.000
4 HS vs 5 HC (discovery; ER167, 141 genes)	power	25	2.0	200	135	1.000	0.000	1.000
4 HS vs 5 HC (discovery; ER167, 141 genes)	power	50	0.5	200	135	0.455	0.035	0.900
4 HS vs 5 HC (discovery; ER167, 141 genes)	power	50	1.0	200	135	1.000	0.000	1.000
4 HS vs 5 HC (discovery; ER167, 141 genes)	power	50	1.5	200	135	1.000	0.000	1.000
4 HS vs 5 HC (discovery; ER167, 141 genes)	power	50	2.0	200	135	1.000	0.000	1.000
3 vs 3 (within-state Mono_FCN1_infl; ER167, 52 genes)	type I error	–	0.0	200	41	0.070	0.018	0.060
3 vs 3 (within-state Mono_FCN1_infl; ER167, 52 genes)	power	10	0.5	200	41	0.050	0.015	0.125
3 vs 3 (within-state Mono_FCN1_infl; ER167, 52 genes)	power	10	1.0	200	41	0.125	0.023	0.265
3 vs 3 (within-state Mono_FCN1_infl; ER167, 52 genes)	power	10	1.5	200	41	0.305	0.033	0.530
3 vs 3 (within-state Mono_FCN1_infl; ER167, 52 genes)	power	10	2.0	200	41	0.600	0.035	0.775
3 vs 3 (within-state Mono_FCN1_infl; ER167, 52 genes)	power	25	0.5	200	41	0.095	0.021	0.140
3 vs 3 (within-state Mono_FCN1_infl; ER167, 52 genes)	power	25	1.0	200	41	0.455	0.035	0.600
3 vs 3 (within-state Mono_FCN1_infl; ER167, 52 genes)	power	25	1.5	200	41	0.870	0.024	0.970
3 vs 3 (within-state Mono_FCN1_infl; ER167, 52 genes)	power	25	2.0	200	41	0.980	0.010	1.000
3 vs 3 (within-state Mono_FCN1_infl; ER167, 52 genes)	power	50	0.5	200	41	0.155	0.026	0.305
3 vs 3 (within-state Mono_FCN1_infl; ER167, 52 genes)	power	50	1.0	200	41	0.775	0.030	0.960
3 vs 3 (within-state Mono_FCN1_infl; ER167, 52 genes)	power	50	1.5	200	41	0.990	0.007	1.000
3 vs 3 (within-state Mono_FCN1_infl; ER167, 52 genes)	power	50	2.0	200	40	1.000	0.000	1.000

Counts were simulated from negative-binomial distributions with the observed library sizes and normalization factors, the fitted control-group abundance of each gene as baseline, and the tagwise dispersion estimates treated as known; affected genes were assigned the stated absolute log2 fold-change with a random sign. Genes were simulated independently, which the real data violate, so the rejection rates are optimistic. The rejection rate at a fold-change of zero

estimates the type I error at a nominal level of 0.05. The three-versus-three design uses three HS and three control donors of the Mono_FCN1_infl state, the minimum number of donors per group required for a within-state test; the within-state tests of Table S7B used four HS and five control donors and were not simulated separately.

Table S17B. Simulated gene-level power of the discovery design (4 HS vs 5 HC; edgeR quasi-likelihood test; FDR<0.05)
Main-text linkage: Methods 2.8; power Results; Figure S12a.

True absolute log2 fold-change	Power, all affected genes (%)	Power, low-expression tertile (%)	Power, middle tertile (%)	Power, high-expression tertile (%)	Observed false-discovery proportion (%)	Replicates
0.5	0.0	0.0	0.0	0.0	9.8	100
1.0	0.6	0.3	0.6	0.7	7.2	100
1.5	55.0	36.9	61.0	66.7	4.3	100
2.0	81.7	64.7	88.7	91.7	4.2	100
3.0	92.7	81.9	97.7	98.6	4.1	100

Same simulation model as in Table S17A. Power is the proportion of truly affected genes detected at a Benjamini-Hochberg FDR<0.05.

Table S18A. Effect sizes for the change in ER167 score after 12 weeks of adalimumab (GSE213761)
Main-text linkage: Methods 2.12; treatment Results; Figure S13; Table S10C.

Quantity	n	Mean	SD	95% confidence interval (t)	Paired t-test P	95% confidence interval (bootstrap percentile)	Hedges g (paired)	95% confidence interval of g	Wilcoxon signed-rank P	Smallest change detectable with 80% power
Lesional ER167 score, week 12 minus baseline	14	0.143	0.557	−0.18 to 0.47	0.353	−0.14 to 0.43	0.24	−0.26 to 0.74	0.426	0.451
Lesional minus non-lesional score at baseline	13	0.084	0.599	−0.28 to 0.45	0.624	−0.22 to 0.40	0.13	−0.38 to 0.64	0.588	–
Lesional minus non-lesional score at week 12	13	0.343	0.418	0.09 to 0.60	0.0121	0.13 to 0.57	0.77	0.16 to 1.35	0.0061	–
Change in lesional minus non-lesional score, week 12 minus baseline	13	0.259	0.743	−0.19 to 0.71	0.233	−0.13 to 0.63	0.33	−0.21 to 0.85	0.191	–

ER167 score: mean z-standardised log2 counts per million across the detected ER167 genes, with z-scores calculated over all samples of the cohort. Fourteen patients had paired lesional samples; 13 had lesional and non-lesional samples at both visits. Bootstrap: 10,000 resamples. The smallest detectable change was calculated for a two-sided paired t-test at α=0.05 with the observed standard deviation of the differences.

Table S18B. Change in lesional ER167 score by responder status (GSE213761; exploratory)
Main-text linkage: Methods 2.12; treatment Results; Figure S13.

Quantity	n	Mean	SD	95% confidence interval	t-test P	Hedges g	95% confidence interval of g	Wilcoxon or Mann-Whitney P
Change in lesional score, responders	7	0.106	0.370	−0.24 to 0.45	0.479	0.25	−0.42 to 0.90	0.812
Change in lesional score, non-responders	7	0.181	0.730	−0.49 to 0.86	0.536	0.22	−0.45 to 0.86	0.578
Difference in mean change, responders minus non-responders	14	−0.076	–	−0.78 to 0.63	0.812	–	–	0.71

Responder status was taken from the sample characteristic 'responder' of the GEO record, which is not defined in the record. The associated publication (Hambly et al., Br. J. Dermatol. 2023, 188, 52–63; reference 25 of the main text) assessed response with the Hidradenitis Suppurativa Clinical Response and the International Hidradenitis Suppurativa Severity Score System; which criterion the GEO field encodes is not stated, and no definition was assumed. Within-group rows: paired t-test and Wilcoxon signed-rank test; difference row: Welch t-test and Mann-Whitney test.

Table S18C. Effect size for the difference in lesional ER167 score after 12 weeks of brodalumab (GSE189266)

Main-text linkage: Methods 2.12; treatment Results; Table S10C.

Quantity	n (baseline / week 12)	Baseline: mean (SD)	Week 12: mean (SD)	Difference	95% confidence interval (Welch)	Welch t-test P	Hedges g	95% confidence interval of g	Mann-Whitney P	Smallest difference detectable with 80% power
Lesional ER167 score, week 12 minus baseline (unpaired)	9 / 9	0.138 (0.370)	0.155 (0.462)	0.017	−0.40 to 0.44	0.931	0.04	−0.84 to 0.92	1	0.588

The GEO record does not link samples to patients, so the comparison is cross-sectional (nine lesional samples at each visit). ER167 score as in Table S18A. The smallest detectable difference was calculated for a two-sided two-sample t-test at α=0.05 with nine samples per group and the pooled standard deviation.

Table S18D. Disease-treatment ER167 vector correlations evaluated against permutation of sample labels and against random gene sets

Main-text linkage: Methods 2.12; treatment Results; Figure S6; Table S10F.

Disease reference	Treatment	ER167 genes	Observed Spearman ρ	Permutation of sample labels	Permutation P (two-sided)	Permutation P (one-sided, ρ<0)	Median of permuted ρ	2.5th–97.5th percentiles of permuted ρ	ρ across all shared genes (n)	Random gene sets: mean ρ	Random gene sets: 2.5th–97.5th percentiles	Percentile of ER167 among random gene sets	Empirical P against random gene sets (one-sided)
GSE154773	adalimumab	156	0.517	Baseline and week-12 labels exchanged within patients (14 patients)	0.110	0.945	0.001	−0.590 to 0.587	0.062 (14,646)	0.026	−0.147 to 0.196	100.00	1/10,001
GSE154773	brodalumab	152	0.219	Time-point labels permuted among the 18 lesional samples	0.702	0.653	−0.013	−0.594 to 0.595	0.059 (13,562)	0.056	−0.112 to 0.223	97.12	0.0289
GSE249027	adalimumab	157	0.394	Baseline and week-12 labels exchanged within patients (14 patients)	0.324	0.840	0.000	−0.590 to 0.594	0.242 (15,784)	0.240	0.076 to 0.397	97.24	0.0277
GSE249027	brodalumab	155	0.426	Time-point labels permuted among the 18 lesional samples	0.573	0.717	−0.015	−0.792 to 0.789	−0.004 (15,350)	0.018	−0.148 to 0.184	100.00	1/10,001

Disease vectors: ER167 gene-level log2 fold-changes of GSE154773 (HS versus healthy skin) and GSE249027 (lesional versus non-lesional skin); treatment vectors: week 12 versus baseline. Permutation of sample labels: 10,000 permutations; in each, the treatment model (limma-voom; patient blocking for adalimumab) was refitted and the correlation with the fixed disease vector recalculated; two-sided P=(1 + number of permutations with an absolute correlation at least as large as that observed)/10,001; the one-sided P for a negative correlation (the reversal hypothesis) is (1 + number of permutations with a correlation not larger than that observed)/10,001. Random gene sets: 10,000 sets of non-ER167 genes of the same size, matched for decile of mean expression; the empirical P value is (1 + number of random sets with a correlation at least as large as that of ER167)/10,001. The two reference distributions answer different questions: the first asks whether the correlation exceeds what arises from sampling variation between samples, the second whether ER167 genes are more concordant than other genes in the same samples. Reversal of the disease-associated pattern would give a negative correlation.
